# Dirhodium Complexes Heterochiral-at-the-Metal Centers: An Alternative Type of Paddlewheel Catalyst for Asymmetric Synthesis

**DOI:** 10.1021/jacs.5c03567

**Published:** 2025-04-04

**Authors:** Matthias Peeters, Lorenzo Baldinelli, Sofia Lerda, Giovanni Bistoni, Alois Fürstner

**Affiliations:** † Max-Planck-Institut für Kohlenforschung, D-45470 Mülheim/Ruhr, Germany; ‡ Department of Chemistry, Biology, and Biotechnology, University of Perugia, I-06123 Perugia, Italy

## Abstract

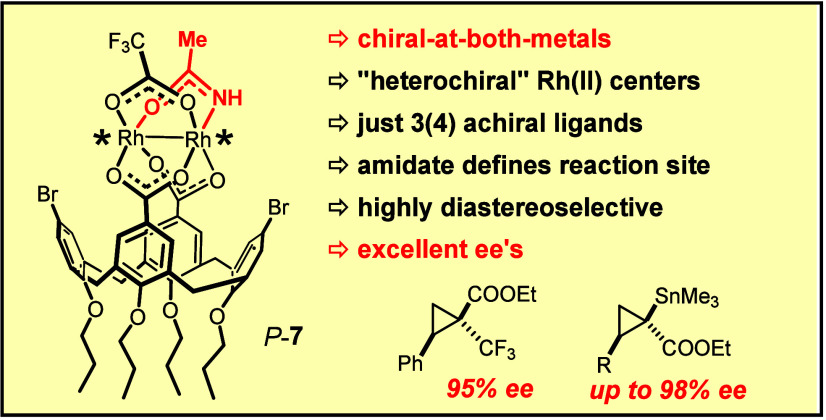

The prototype of an entirely new class of dirhodium paddlewheel complexes is disclosed, which is chiral at both inequivalent metal centers although it carries just three different achiral μ-bridging equatorial ligands. One of them is a carboxamidate, which ensures selective carbene formation at the Rh[O_3_N] face of the catalyst; moreover, the −NH group engages the ester carbonyl of the emerging rhodium carbene intermediate derived from α-stannylated α-diazoacetate in interligand hydrogen bonding, which is critically important for controlling the stereochemical course of the ensuing [2 + 1] cycloaddition. The new heterochiral catalyst furnished stannylated cyclopropane derivatives with excellent diastereo- and enantioselectivity; moreover, a cyclopropane with a quaternary trifluoromethylated stereocenter was obtained with an unprecedented level of asymmetric induction.

Countless chiral dirhodium “paddlewheel” complexes are known in the literature, but their constitution is strikingly uniform in conceptual terms (Scheme 1A):^1−3^ the overwhelming majority of them are homobimetallic and homoleptic entities of type **A** endowed with a set of stereogenic equatorial ligands. Although not limited to them, chiral carboxylates and carboxamidates are most common; catalysts of this type found innumerous applications in advanced organic synthesis and beyond.^4−16^ Despite this enormous success, experimental insights into the structure of the reactive intermediates generated in situ have been gained only recently, which stimulate new catalyst design.^17,18^ As a consequence, increasing attention is focused toward heteroleptic or heterobimetallic analogues; the results obtained with complexes such as **B** and **C** encourage further exploration of this still poorly charted chemical space.^19−22^

**Scheme 1:**
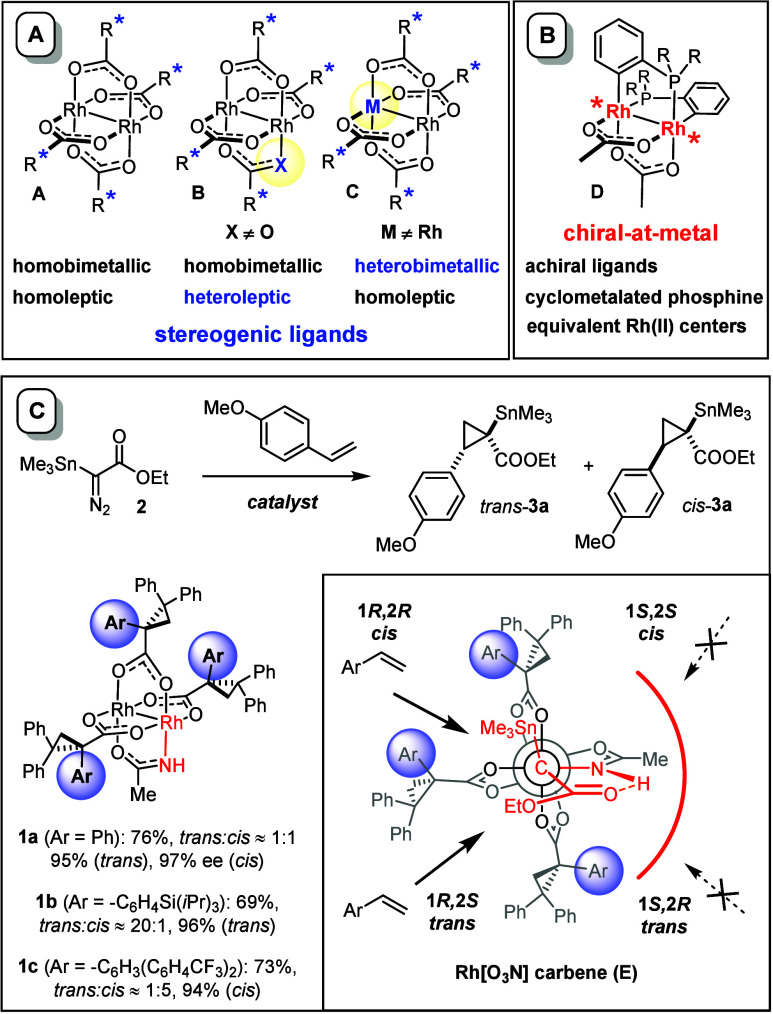
Top: Established Formats of Chiral Rhodium Paddlewheel Complexes; Bottom: Heteroleptic Catalysts for Asymmetric Cyclopropanation with Stannylated Diazoacetates

To the best of our knowledge, however, there is only a single class of chiral dirhodium “paddlewheel” complexes of fundamentally different format, in that the rhodium atoms themselves are stereogenic whereas the ligands are not.^23^ Specifically, the reaction of, e.g., [Rh_2_(OAc)_4_] with R_2_PAr in refluxing toluene/HOAc affords doubly *ortho*-metalated complexes of type **D** (Scheme 1B).^24^ Because the metalated phosphines ligands are in a *cisoid* head-to-tail arrangement, such complexes exhibit “backbone chirality” and the enantiomers are separable by resolution;^25,26^ they comprise two equivalent, “homochiral” rhodium centers. Applications to a small set of asymmetric transformations were met with respectable success, although the results were strongly case dependent.^25−27^

From the conceptual viewpoint, a cyclometalated backbone and phosphine ligands may not be ideal; they reduce the catalytic activity due to an enhanced electron density at the metal center,^28^ and the Rh–C bond itself is potentially reactive. Therefore, we considered an alternative concept that extrapolates the structural features of heteroleptic complexes of type **1** previously developed in our group (Scheme 1C). These catalysts were deemed a good starting point for their, so far, unique ability to effect the cyclopropanation of olefins with stannylated α-diazoacetate **2** with excellent levels of asymmetric induction.^29−31^ The C–Sn bond provides a valuable handle for functionalization of the resulting products **3**, which have already served natural product synthesis well.^32^ Key to success is the formation of an interligand hydrogen bond between the −NH group of the amidate and the ester carbonyl of the emerging rhodium carbene **E**. Computational and spectroscopic data indicated that this structural motif controls the downstream chemistry, in that attack of the incoming nucleophile from the sterically more open quadrants would perturb this stabilizing interaction and is hence disfavored.^30,33^ The fact that the hydrogen bond locks the reactive species in place within the chiral binding pocket fosters the level of asymmetric induction.

Formally, it suffices to replace the three identical chiral carboxylates of **1** by three different achiral carboxylates to generate an entirely new class of dirhodium paddlewheel complexes **F** that is chiral-at-both-metals (Scheme 2); in an even more minimalistic approach, two different achiral carboxylates are enough if the identical equatorial ligands are placed *cis* to each other, which can be ensured by tethering them together. Importantly, the two rhodium centers of **F** are inequivalent (in contrast to the situation in the “homochiral” complex **D**); yet, the presence of a carboxamidate in **F** should determine the site at which carbene formation will take place.^33^ In the case of carbene **G** derived from the stannylated diazoester **2**, the ensuing hydrogen-bonded array should again make front-side attack by the olefinic reaction partner unfavorable; at the same time, the tether between the R^3^ substituents blocks the backside trajectory leading to the *trans*-configured product. Should a highly *cis*-selective catalyst become available in this way, it would nicely complement the existing portfolio and close a certain gap in coverage.^30^

**Scheme 2:**
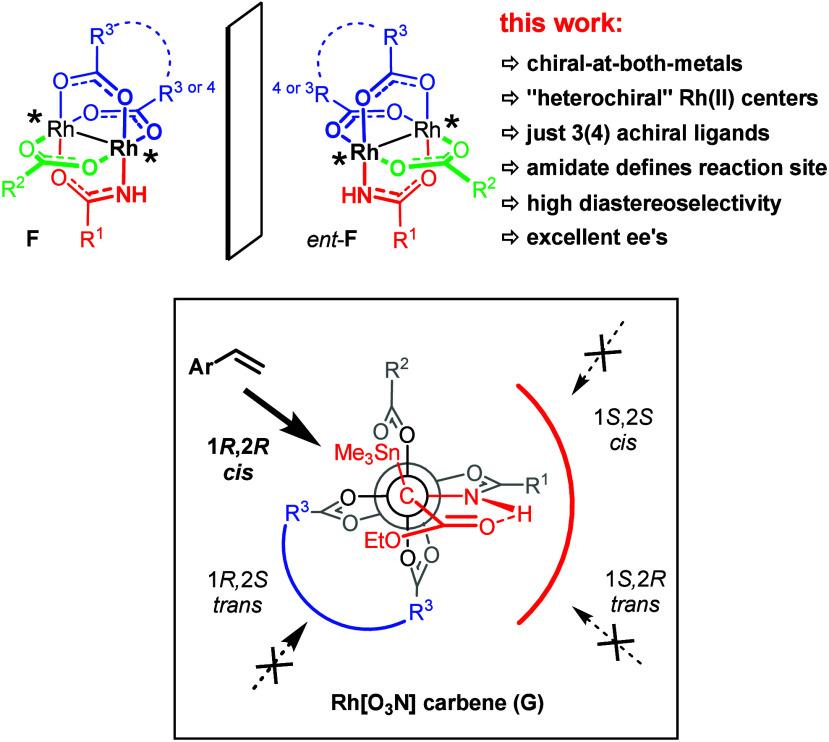
A New Type of Heteroleptic Chiral-at-Metal Dirhodium Complexes with Inequivalent Rh-Centers

Intrigued by this outlook, we ventured into the synthesis of a prototype catalyst of this new family (Scheme 3). The challenge of introducing different equatorial ligands into the coordination sphere of a dirhodium paddlewheel complex, however, must not be underestimated. We finally succeeded by first exposing [Rh_2_(acam)_4_] (**4**) to neat trifluoroacetic acid at 60 °C; it is imperative to stop the reaction after 20–25 min to secure a good yield of the heteroleptic product **5** on >400 mg scale. **5** was then treated with a slight excess of the known calix[4]arene dicarboxylic acid **6** to give the desired complex *rac*-**7**. Ligand **6** was chosen for a number of reasons: (i) this particular calix[4]arene derivative is known to be cone-shaped, thus ensuring that the two carboxylic acid moieties on the upper rim are accurately preoriented for complexation;^34−36^ (ii) the backbone is not overly stiff and should hence be able to adapt to the geometric constraints of the rigid dirhodium core which it has to ligate; actually, an achiral dirhodium catalyst carrying two such bidentate ligands is known;^37^ (iii) the diameter of the calix[4]arene backbone matches the size of the dimetallic core; as a result, the bromo-substituents on the two other alternating benzene rings are placed in proximity of the axial coordination sites of the rhodium atoms, where they can steer the catalytic transformation without preventing it from happening; even synergistic interactions with the catalyst core are conceivable;^38^ (iv) the propyl ethers on the lower rim ensure solubility of the resulting complex in nonpolar media; (v) diacid **6** is readily accessible and the synthesis scalable;^34,39^ (vi) finally, further ligand modifications can be envisaged at a later stage, should the overall design turn out to be promising.

**Scheme 3:**
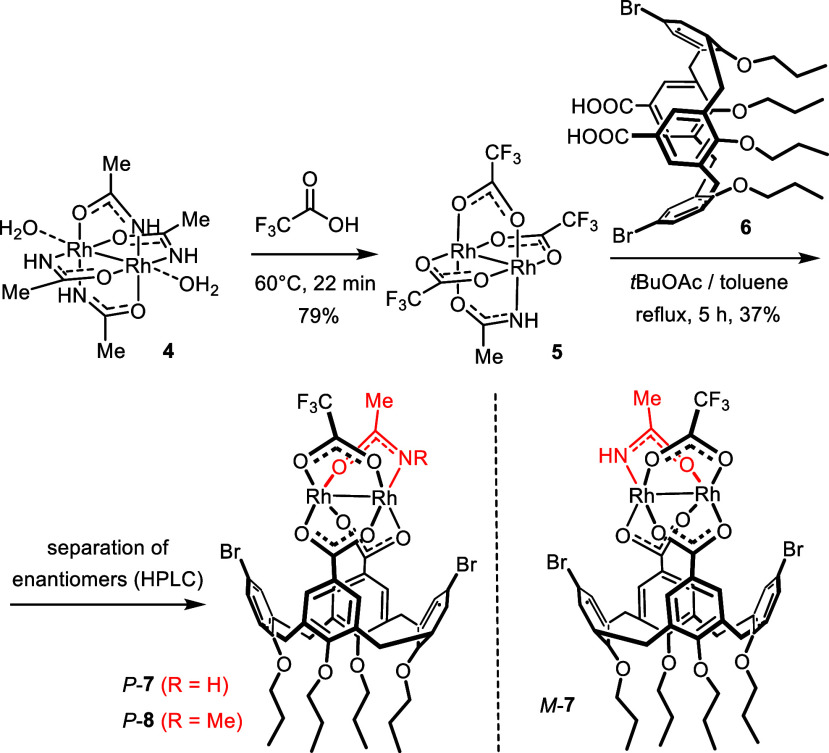
Preparation of a Prototype Catalyst

The enantiomers of **7** were separated by HPLC on a chiral stationary phase. Since the acquired XRD diffraction data were of insufficient quality, the configuration was assigned by comparison of the experimental with the computed electronic CD spectra (Figure 1). These complexes are helically chiral entities and hence designated *P*-**7** and *M*-**7**.^40−42^

**Figure 1:**
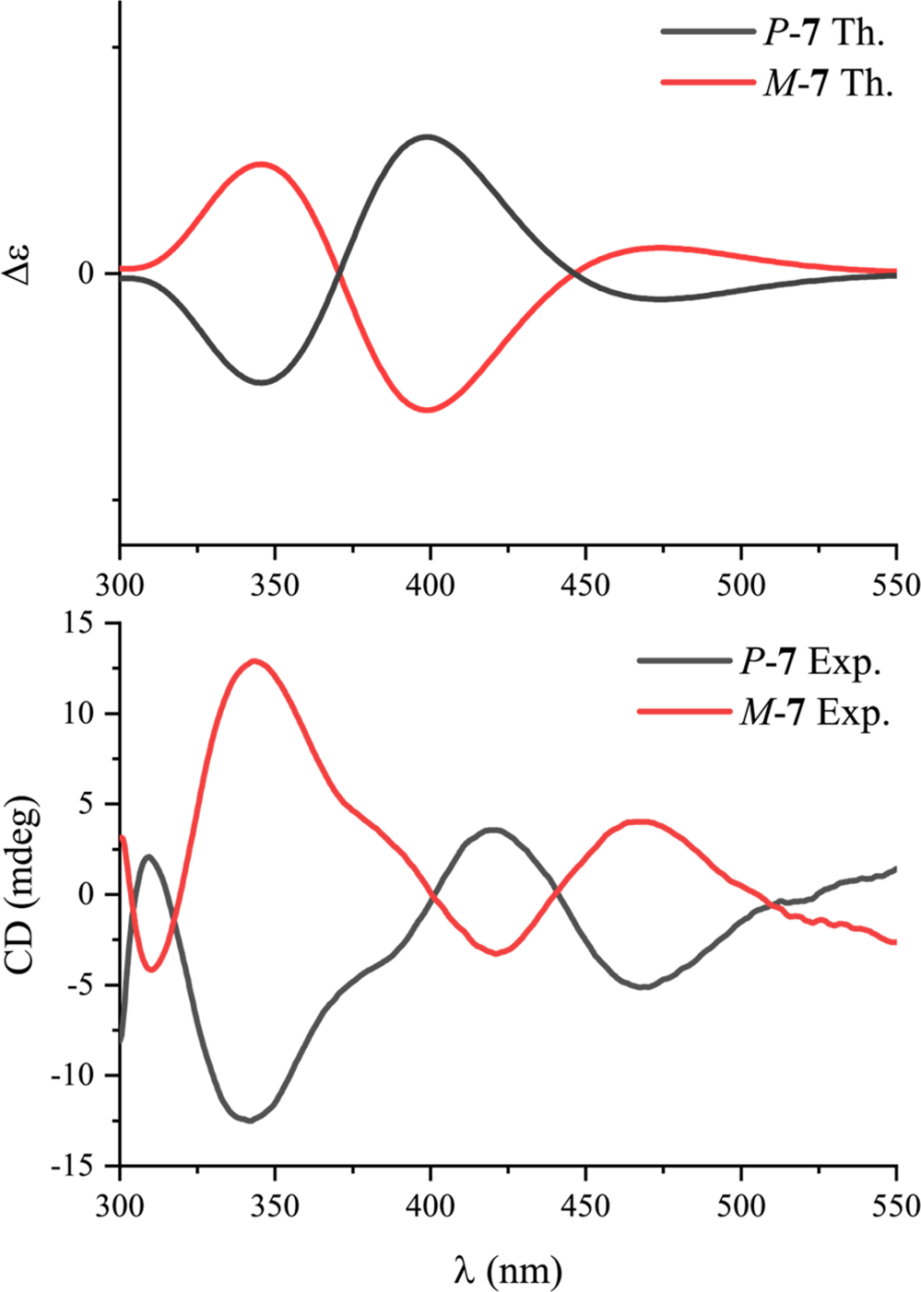
Boltzmann-weighted computed electronic CD spectra (top) and experimental CD data (bottom) of the catalyst enantiomers, *P***-7** and *M***-7**.

Upon slow addition of a solution of the stannylated diazoester **2**(30,43) to a solution of *p*-methoxystyrene and *P*-**7** (0.5 mol %) in CH_2_Cl_2_, the desired product **3a** was formed in modest yield (37%, NMR); however, the diastereoselectivity in favor of the *cis*-isomer and the optical purity were remarkably high (dr ≈ 10:1, 98% ee).^44^ The yield proved to be strongly correlated with the addition time; somewhat counterintuitively, fast injection (<1 min) of the diazo derivative resulted in much cleaner transformations without compromising the selectivity (Scheme 4A).^45^ Thus, **3a** was isolated by flash chromatography as the pure *cis*-isomer in 58% yield with 98% ee; the assignment as (1*R*,2*R*)-isomer is unambiguous by comparison with our earlier work.^29,30^ The new chiral-at-both-metal catalyst *P*-**7** hence compares favorably to **1c** as the best *cis*-selective catalyst previously known, which had furnished **3a** with a notably lower *cis:trans* = 5:1 ratio.^30,31^

**Scheme 4:**
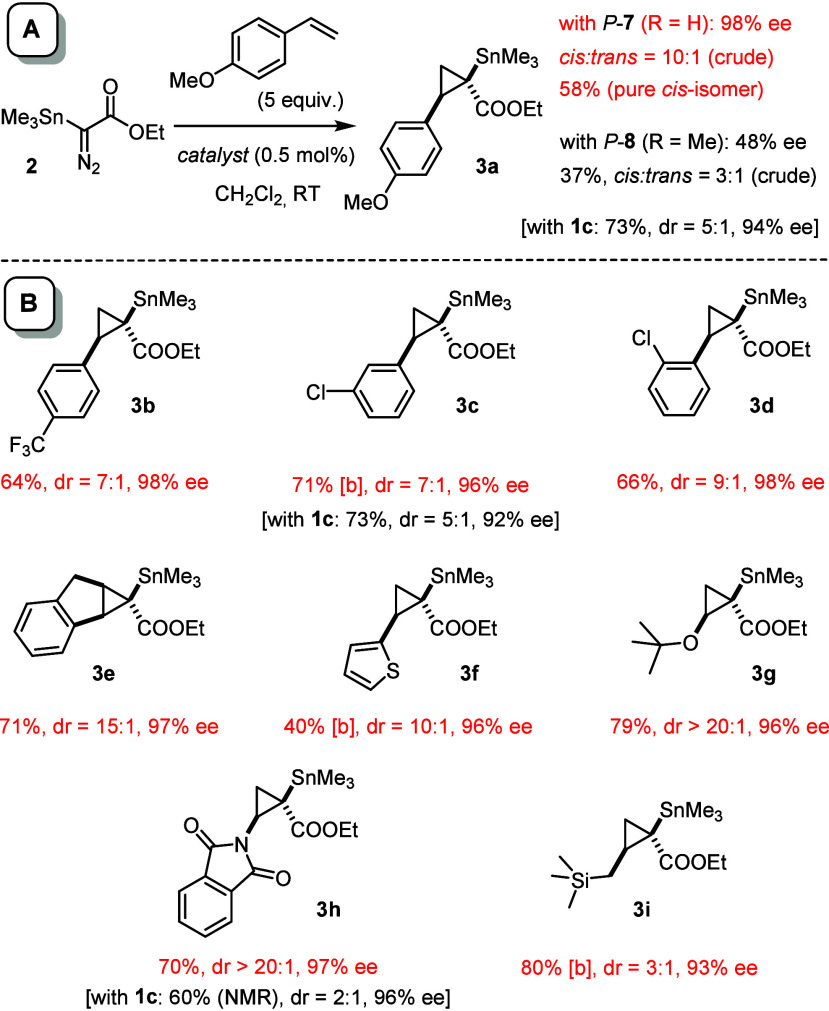
*cis*-Selective Stannylcyclopropanations Unless stated otherwise, the yields refer to pure *cis*-isomer after flash chromatography, the dr to the crude product (NMR). Mixture of the *cis*- and *trans*-isomers.

A control experiment with catalyst *P*-**8**^46^ featuring an *N*-methyl substituent on the acetamidate ligand was informative: the yield, dr and ee of **3a** all dropped significantly, which suggests that the protic −NH group in *P*-**7** is quintessential for success. This finding together with the highly *cis*-selective course of the reaction lends credence to the notion that the basic design features outlined above capture the essence of how the new chiral-at-metal catalyst operates; in any case, it supports the notion that the reaction proceeds at the chiral Rh-center ligated to the N atom.^33^

A preliminary exploration of the substrate scope confirmed the excellent performance of *P*-**7**. Scheme 4B summarizes the results; where available, the data obtained with **1c** as the catalyst giving the highest *cis*-selectivity so far are included for comparison.^30^ In all cases is the diastereoselectivity attained with *P*-**7** higher and the optical purity of the stannylated cyclopropanes outstanding. The advance over the prior art is particularly evident from product **3h** derived from N-vinylphthalimide;^30^ replacement of **1c** by *P*-**7** increased the yield and the dr in this case from 2:1 to >20:1, close to the limits of detection of the second isomer. *tert*-Butyl vinyl ether reacted similarly well to give **3g** (dr >20:1, 96% ee). Another rewarding finding is the fact that the electronic character of a styrene substrate exerts a negligible influence on the outcome as evident from a comparison of products **3a** and **3b**. Likewise, the tolerance of a hindering *ortho*-substituent, as manifested in **3d**, is noteworthy. In the past, allyltrimethylsilane had only been subjected to a *trans*-selective cyclopropanation with diazoester **2**, whereas a complementary *cis*-selective reaction had not been accomplished;^30^ this gap is now closed, although the dr of **3i** carrying two different metalloid handles for downstream functionalization is lower than in the other cases.

Given the novelty of the catalyst design, a more detailed analysis of the mode of action of *P*-**7** was deemed appropriate. For systems of this size, a reliable computational protocol must combine a thorough exploration of the chemical space with a careful treatment of all relevant electronic structure effects.^47^ The chosen approach follows the methodology successfully applied in previous studies on related systems,^30,33^ which reproduced or predicted key experimental observations with high accuracy. Specifically, the stannylated carbene intermediate generated at the Rh[O_3_N] face of *P*-**7** was computed. After a conformational sampling without any constraint via the CREST program^48^ using the GFN2-xTB semiempirical method,^49,50^ an in-house code was used to compare RMSDs across the computed conformational space. The geometries of the selected small subset of conformers were refined using the B3LYP-D3(BJ) functional and the def2-SVP basis set^51^ with the RIJCOSX approximation.^52−54^ Implicit solvation effects at the CPCM level for CH_2_Cl_2_ were included to reproduce experimental reaction conditions as closely as possible.^55,56^ Free energies were obtained by adding thermal corrections computed at the B3LYP-D3BJ/def2-SVP+RIJCOSX+CPCM(DCM) level of theory to electronic energies calculated at B3LYP-D3BJ/def2-TZVP(-f)+RIJCOSX+CPCM(DCM).^57^ These methods were also used to compute the transition states (TS) leading from the metal carbene to all four possible cyclopropane isomers in order to gain insights into the factors controlling the stereochemical outcome of the reaction with *p*-methoxystyrene as the model substrate.

The by far lowest-lying TS shows *p*-methoxystyrene attacking via the top-back quadrant (Figure 2); the bulky Me_3_Sn- group points “upward” to reside in fairly unencumbered space, which is another critical determinant of the diastereoselectivity.^58^ In full agreement with experiment, the *cis*-(1*R*,2*R*)-configured cyclopropane **3a** is hence computed to be the major product. Even though the front side of the reactive intermediate is wide open in geometric terms, high barriers were found, which originate from a massive distortion of the lateral H-bond between the −NH group on the catalyst and the ester carbonyl of the carbene if the olefin approaches from this face. Conversely, the lower left quadrant is evidently blocked by the bulky calix[4]arene; as a result, a very high barrier does ensue. The large differential of the TS-energies of the two *cis*-channels (ΔΔG^‡^ = 7.42 kcal·mol^–1^) is perfectly in line with the attained ee of 98%.

**Figure 2:**
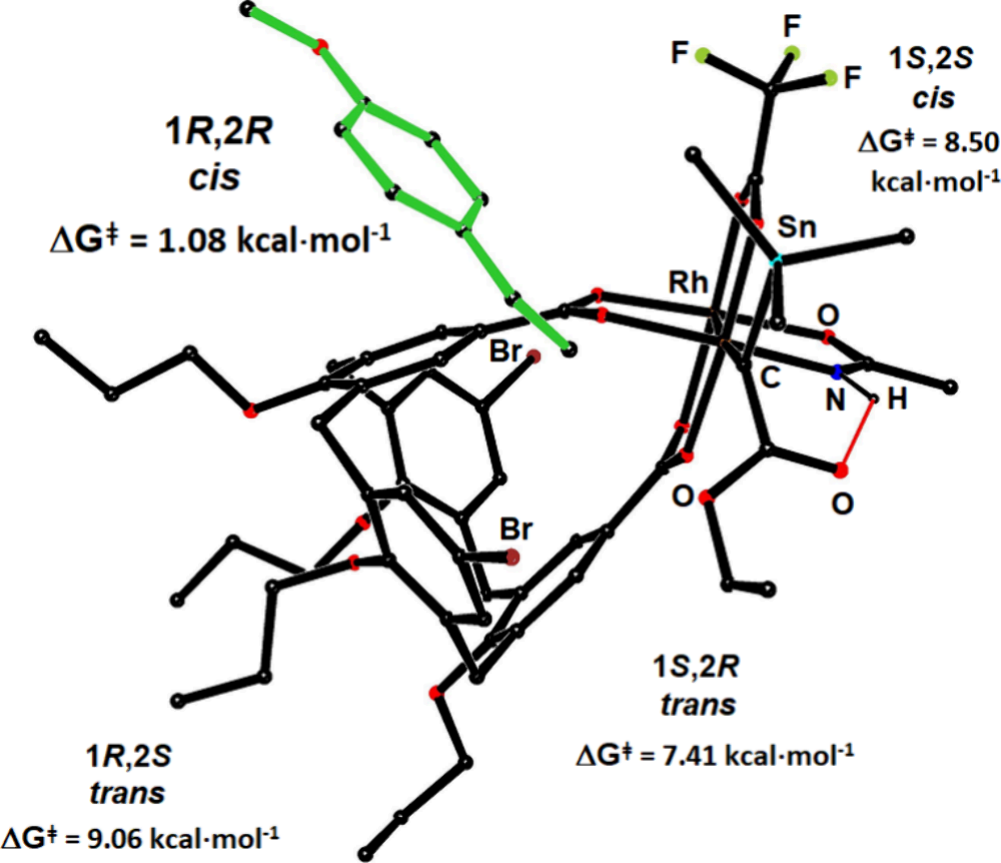
Computed structure of the transition state (TS) for the reaction of the stannylated carbene intermediate formed at the Rh[O_3_N] face of *P*-**7** with *p*-methoxystyrene (green) leading to (1*R*,2*R*)-**3a**; the lateral NH···O bond is shown as a red line. The TS-energies for approaches via the other quadrants are included for comparison.

In contrast, the computations predict that the minor *trans*-isomer should be of comparatively low optical purity (ΔΔG^‡^ = 1.65 kcal·mol^–1^). HPLC allowed this mechanistic implication to be verified in several cases (*trans*-**3b**: 57% ee; *trans*-**3c** 38% ee; *trans*-**3i**: 4% ee). This finding provides additional confirmation that the analysis outlined above is accurate.

More systematic studies into this new catalyst chemotype have to await further study, but the promise is high (Scheme 5). While a first application to the donor–acceptor diazo derivative **9** afforded a respectable but not yet optimal result and may make the development of a second catalyst generation necessary, the cyclopropanation of styrene with 3,3,3-trifluoro-2-diazopropionate **11** exceeded the known standards by far: although ten different chiral paddlewheel catalysts had been tested in the past, the best published result was disappointing (50% yield, dr = 44:56, 18% ee (*cis*), 50% (*trans*)).^59−61^*P*-**7**, in contrast, furnished **12** comprising an all-carbon quaternary center carrying a −CF_3_ substituent with 95% ee and a dr = 12:1. For this highly encouraging result, the scope of this and related transformations and further mechanistic studies are being actively pursued in our laboratory.

**Scheme 5:**
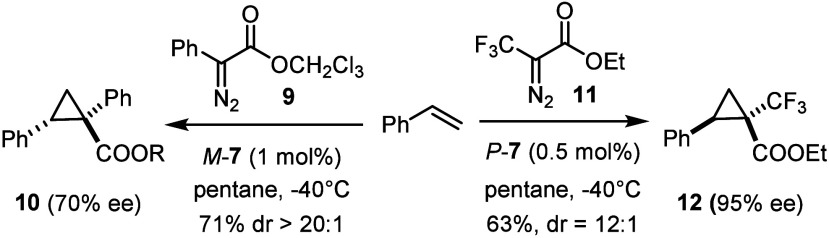
Outlook

## Supporting Information Available

Experimental section including characterization data, copies of spectra of new compounds, and computational details. (PDF)
