# Supplementary material for: Dirhodium Complexes Heterochiral-at-the-Metal Centers: An Alternative Type of Paddlewheel Catalyst for Asymmetric Synthesis
Source: J Am Chem Soc. 2025 Apr 4;147(15):12418–24. doi: 10.1021/jacs.5c03567 (PMC12006997; doi:10.1021/jacs.5c03567)
Supplement: Supplementary file 1 — ja5c03567_si_001.pdf [file ja5c03567_si_001.pdf]

SUPPORTING INFORMATION

Dirhodium Complexes Heterochiral-at-the-Metal Centers: An Alternative Type of Paddlewheel Catalyst for Asymmetric Synthesis

Matthias Peeters,[a] Lorenzo Baldinelli,[b] Sofia Lerda,[b] Giovanni Bistoni,[b]
and Alois Fürstner[a],*

[a] Max-Planck-Institut für Kohlenforschung, D-45470 Mülheim/Ruhr, Germany
[b] Department of Chemistry, Biology, and Biotechnology, University of Perugia, I-06123 Perugia, Italy

fuerstner@kofo.mpg.de

Table of Contents

Nomenclature S2
The Specific Problem S2
Generalization S3
Computational Details S5
Generation of the Structural Ensemble for the Catalyst S5
Protocol Validation for UV-Vis and Circular Dichroism Spectra S6
Electronic Circular Dichroism Spectra S9
Geometry of the Stannylated Carbene Intermediate S10
Stannylated Carbene Intermediate: Steric Maps S11
Transition States S12
Cartesian Coordinates S13
Experimental Section S31
General S31
Catalyst Screening and Reaction Optimization S32
Ligand Synthesis S33
New Dirhodium Complexes S35
cis-Selective Formation of Stannylated Cyclopropanes S40
Other Cyclopropanations S46
Spectra S49
References S87

Nomenclature

The Specific Problem

To the best of our knowledge, there is no precedent in the literature for related binuclear complexes that incorporate two inequivalent chiral-at-metal coordination centers. For their designation, the rules of inorganic stereonomenclature were adapted as described below.

The new dirhodium paddlewheel complex **7** can formally be seen as consisting of two mutually interpenetrating octahedra in which the axial coordination sites at the Rh-centers are occupied by the solvent (or the future carbene, a C-based ligand) and therefore given priority 5. The Rh-atoms mutually serve as metallo-ligands to each other as color-coded in Figure S1; as such, each Rh-atom is the ligand of highest priority within the coordination sphere of the other Rh-center ($Rh^{1'}$ is ligand of highest priority for Rh[O4] and vice versa); the X–Rh–Rh–X' unit hence represents the principle reference axis. According to the rules of inorganic stereonomenclature and the CIP priority rules,[1,2] the two chiral-at-metal coordination octahedra must therefore be designated: Rh[O4]: *OC*-6-53-A and Rh[O3N]: *OC*-6-5'3'-C. If one disregards the axial (unknown) ligands X, the analogous designation for the two interpenetrating square pyramides is: Rh[O4]: *SPY*-5-A and Rh[O3N]: *SPY*-5-C.

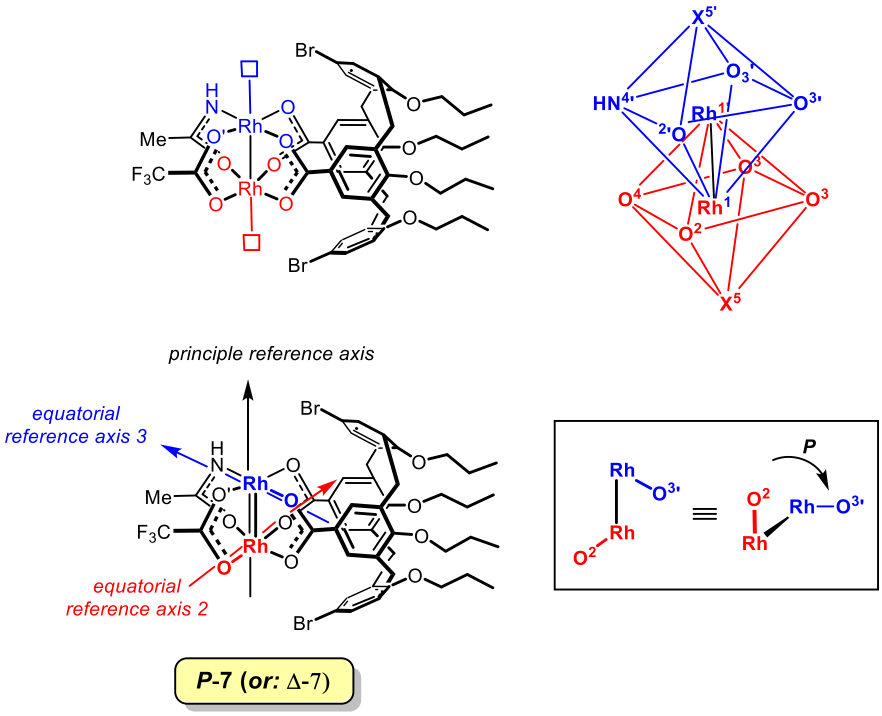

Figure S1. Analysis of the heterochiral dirhodium complex **7** used to define the stereonomenclature. The superscript to the element symbol refers to the ligands priorities within each of the interpenetrating octahedra: for example, Rh^1 means that this rhodium atom is the ligand of highest priority ([1]) in the octahedral coordination sphere of Rh[O3N]. The arrows in black, red and blue in the

representation at the bottom left show the reference axes used to define the helical chirality of the complex

This formalism is neither obvious nor intuitive; to facilitate discussion, complex **7** is therefore regarded as a helically chiral object. To this end, the diagonal axes within the equatorial plane of each Rh-center need to be defined, which contain the ligand of ***relative highest priority and, at the same time, are not collinear to each other***. In complex **7**, the relevant axis at Rh[O4] is passing through O^2 and one of the two $O^{3'}$ atoms (reference axis 2, red); at Rh[O3N], the axis is defined by $O^{3'}$ and $HN^{4'}$ (reference axis 3, blue). If one then looks along the principal Rh–Rh axis from Rh[O4] in the front as the center of higher priority toward Rh[O3N] in the back and spirals from O^2 as the equatorial ligand of highest priority in the front to $O^{3'}$ in the back, the O^2–Rh–Rh–$O^{3'}$ unit describes a clockwise turn (see the Insert in Figure S1); complex **7** is hence ***P***-configured.

In this context, we note that the chiral-at metal paddlewheel complexes of type **D** (see main text) bearing two cyclometallated phosphine ligands described by Lahuerta et al. were also designated using the ***P*** and ***M*** nomenclature.[3] However, the situation is simpler because both Rh-centers are equivalent.

Generalization

The presence of the $–CF_3$ group in **7** results in clear CIP-priorities amongst the four O-atoms in the equatorial plane of Rh[O4] as shown in Figure S1. An additional complication arises in complex **S8**, in which the trifluoroacetate was replaced by an ordinary acetate ligand (Figure S2).

The principle reference axis along the Rh–Rh bond remains the same. The two O-atoms of the calixarene-based chelate ligand then have highest priority in the equatorial plane of both chiral-at-metal rhodium centers but are, *a priori*, equivalent; therefore, it is not intuitive where to start.

In this case, the stereochemical rule of "***trans*-maximum-difference**" is applied:[1,2] that is why the axis O^2–O^4 gains priority over O^2–O^3 at the Rh[O4] center. The axis O2'–$NH^{4'}$ at Rh[O3N] is collinear and therefore irrelevant; for the designation, the non-collinear ligand of highest priority is the $O^{2'}$ substituent in the back and the pertinent axis hence O2'–O3'. On this basis, the chiral axis of complex **S8** is also ***P***-configured, because the O^2–Rh–Rh–$O^{2'}$ unit describes again a clockwise turn.

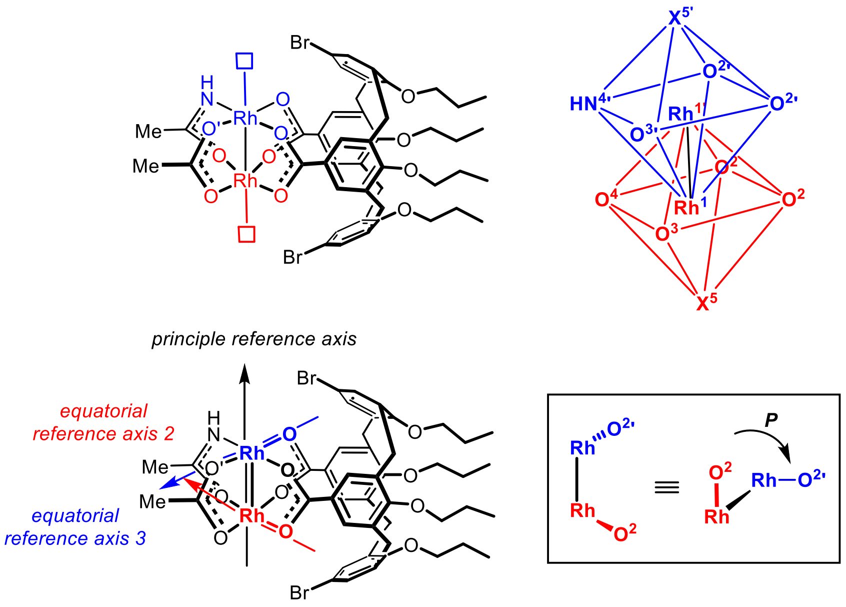

***P*-S8 (*or:* Δ-S8)**

Figure S2. Generalization of the helical stereonomenclature

Computational Details

Generation of the Structural Ensemble for the Catalyst

Conformational sampling was performed using CREST program.[4, 5] The simulations employed the GFN2-xTB//GFN-FF composite methodology:[6,7] sampling and optimizations were performed at the GFN-FF level, complemented by a GFN2-xTB single-point calculation.

Two distinct families of conformers were found for complex **7**, as illustrated in Figure S3. The primary difference between these conformer families lies in the relative orientations of the propoxy substituents of the cyclic ligand. In Family 2, all propoxy substituents are oriented on the same side, whereas in Family 1, their orientation is alternated.

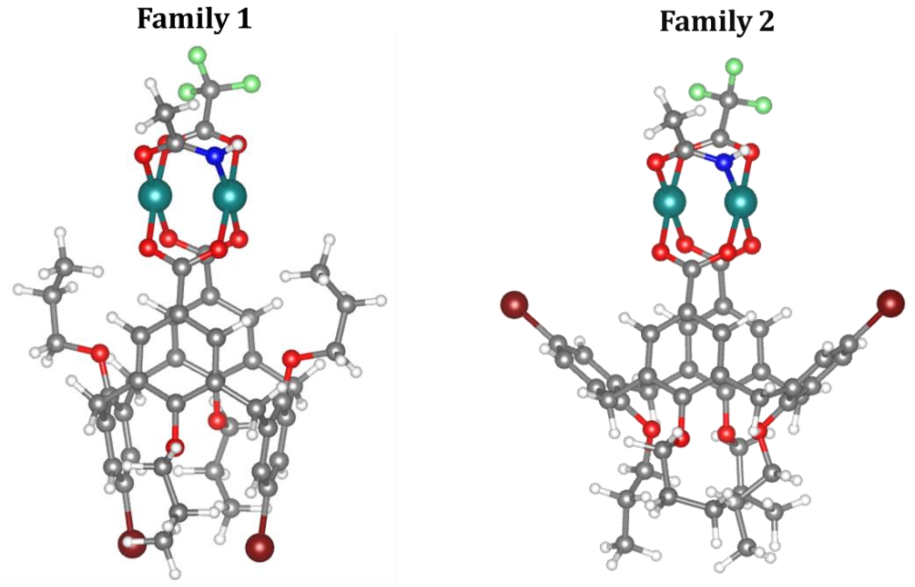

Figure S3. Representative conformers of complex **7** from the two main families identified with unconstrained metadynamics simulations. Family 1 features alternating orientations of the propoxy substituents, while Family 2 is cone-shaped with a uniform orientation.

The exploration of conformational space yielded a substantial number of distinct conformers (on the order of ~10^3). To enable a focused and representative investigation of the optical properties of the catalyst while accounting for conformational diversity, we selected in our initial structural ensemble only a subset of structures, using an in-house code that performs iterative screening of conformers by comparing RMSD values between each conformer and a reference conformer. In each iteration, the code selects the conformer with the lowest energy and a high RMSD compared to the previous reference conformer. This approach ensures a diverse and representative subset of conformers.

All the selected conformers in our initial structural ensamble were reoptimized at the DFT level. These calculations were carried out with a development version of the ORCA quantum package based on v. 5.0.3.[8] Specifically, the geometries of the selected conformers were reoptimized using the using B3LYP-D3(BJ) functional[5,9,10,11,12,13] and Ahlrichs def2-SVP basis set.[14] Implicit solvation effects were incorporated at the CPCM level using the parameters for *tert*-butyl methyl ether.[15,16] The resolution of identity (RI) approximation was used in the RIJCOSX variant together with the corresponding auxiliary basis set.[17,18,19] Subsequently, the electronic energies were further refined at the wB97X/def2-TZVP(-f) level, again incorporating RIJCOSX and CPCM.[20]

Protocol Validation for UV-Vis and Circular Dichroism Spectra

This refinement of both geometry and electronic energy for all conformers in our structural ensemble was essential for accurately modeling the optical properties of the catalyst (all computed at the wB97X/def2-TZVP(-f) + RIJCOSX + CPCM level for consistency). Notably, circular dichroism (CD) spectra are particularly sensitive to geometric changes. Therefore, to address this delicate aspect, the refined electronic energies based on accurate geometries were used to calculate both UV-Vis and CD spectra. These spectra were obtained as a convolution of the individual spectra of each selected conformer, where the contributions are weighted according to the Boltzmann distribution.

This approach significantly improves the representation of the catalyst optical properties under experimental conditions with a simple yet effective protocol, by accounting for the broader conformational landscape that characterizes the real conditions rather than solely relying on just the most stable catalyst structure.

For this analysis, we focused solely on Family 2, as this conformation corresponds to the experimentally observed structure. An energy scan of the dihedral angle responsible for the interconversion from Family 2 to Family 1 revealed that the computed energy barrier is prohibitively high, thus excluding such conversion under experimental conditions (Figure S4).

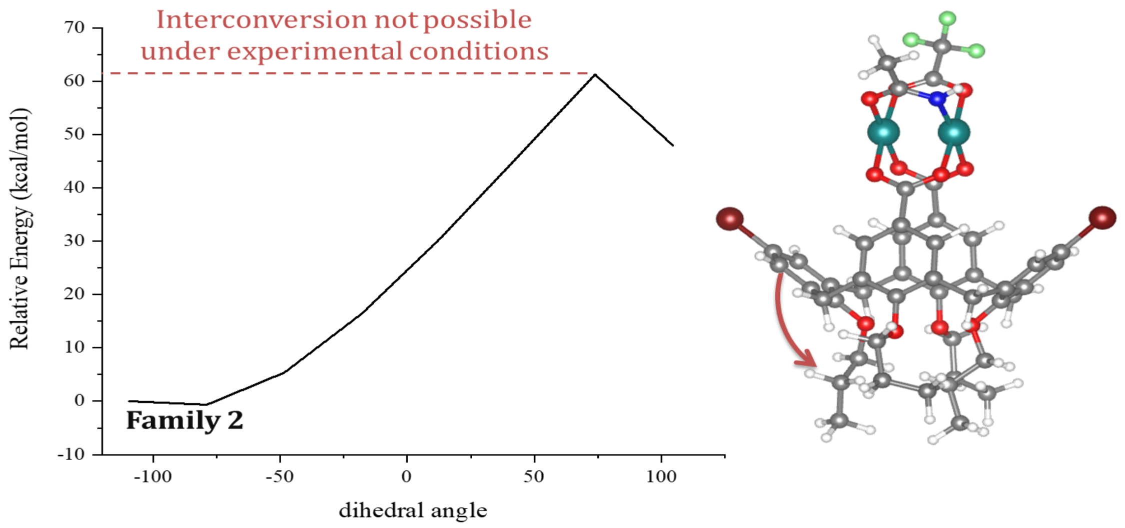

Figure S4. Energetics of the dihedral rotation converting a Family 2 conformer into a Family 1 conformer.

To validate the computational protocol, we first calculated the UV-visible absorption spectra of complex **7** using the chosen level of theory and compared the results with experimental data. An initial comparison, shown in Figure S5, revealed that the position of the main peak is accurately reproduced by our calculations.

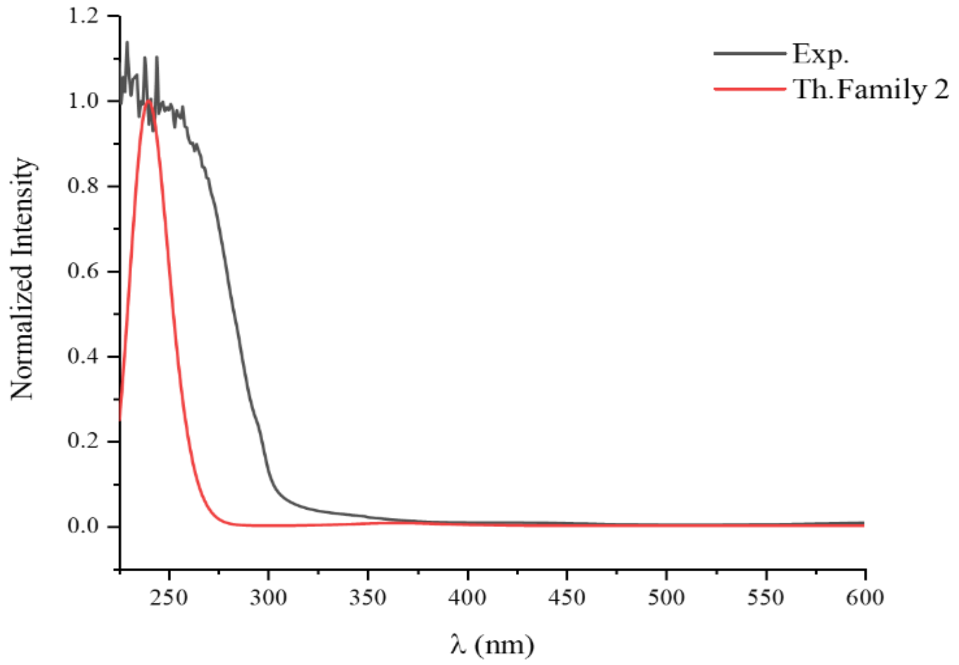

Figure S5. Recorded (black, *tert*-butyl methyl ether) versus computed (red) UV-Vis spectra of complex **7**

The excellent agreement between theory and experiment is further emphasized in Figure S6, where the experimental and calculated spectral features are compared using a different scale in which all significant spectral features can be identified. By analyzing the individual features, we observe that all three peaks observed in the experimental spectrum are accurately reproduced by our calculations.

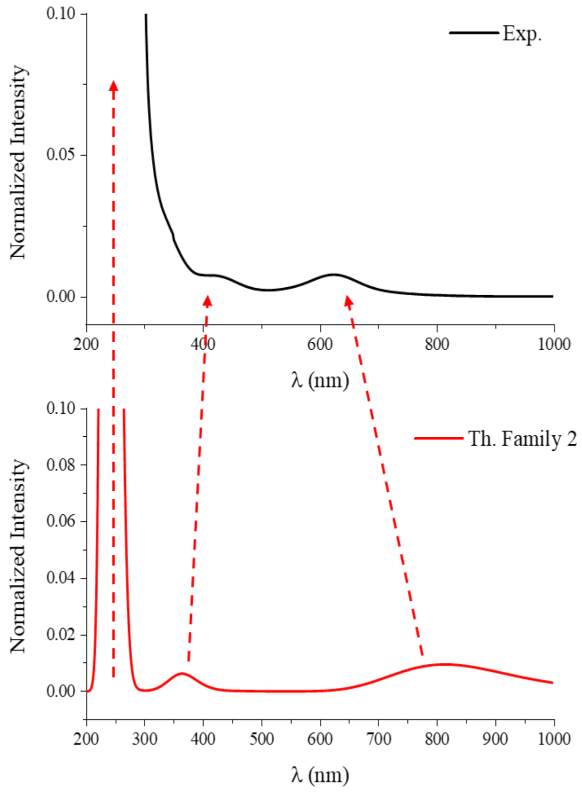

Figure S6. Comparison of experimental and calculated UV-visible absorption spectra.

To explore the nature of the electronic transitions responsible for the key spectral features, isodensity plots were generated and analyzed in detail (Figure S7). The analysis indicates that the two lower-energy peaks arise from metal-to-ligand charge-transfer excitations, reflecting transitions where electronic density shifts from metal-centered orbitals to ligand-centered orbitals. Conversely, the intense and highest-energy peak is attributed mainly to ligand-to-ligand charge-transfer excitations.

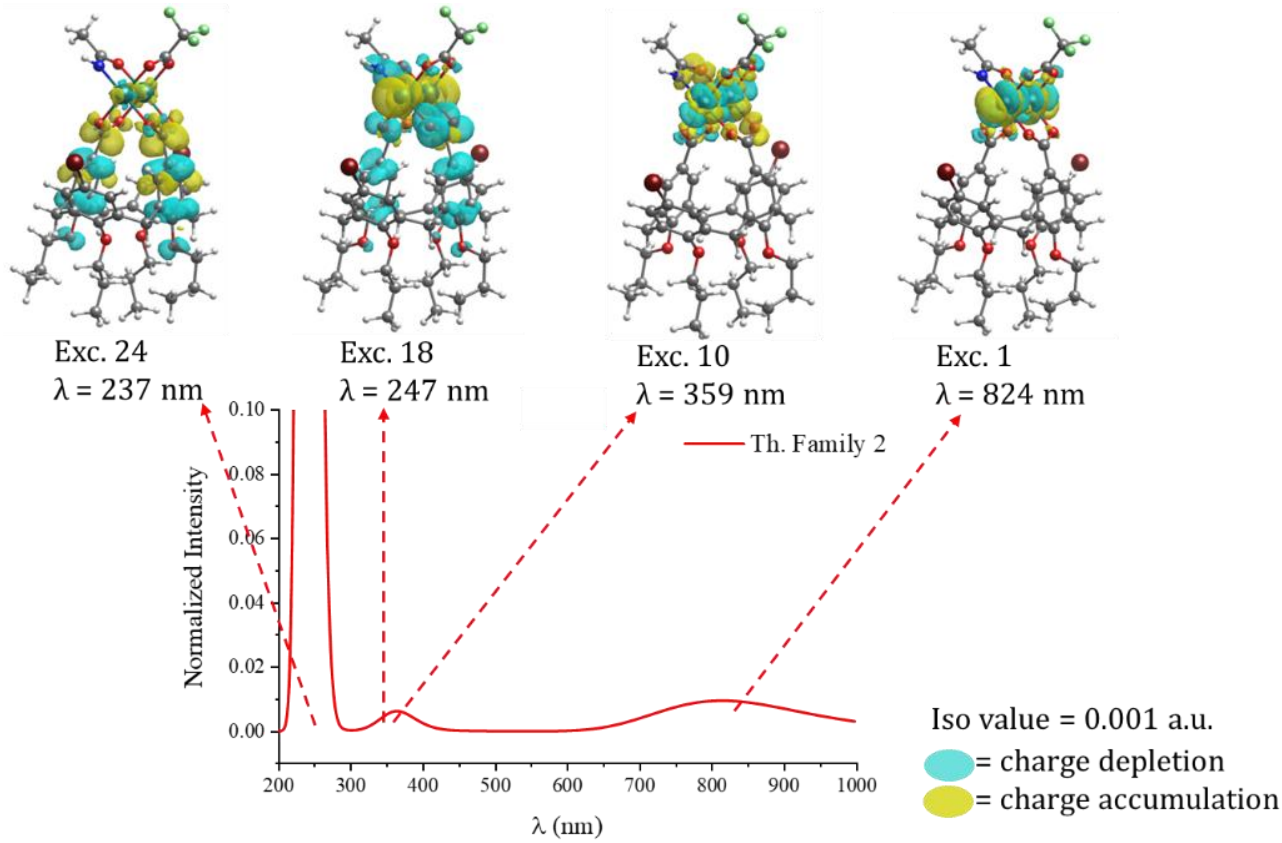

Figure S7. Characterization of the electronic transitions related to the features of the catalyst absorption spectrum.

Electronic Circular Dichroism Spectra

After having validated the computational setup, we proceeded in using the same approach to compute the CD spectra for the two enantiomers of the catalyst, namely *P*-**7** and *M*-**7**. The computed CD spectra were then compared with the experimental data, as shown in Figure S8. The agreement between theory and experiment further confirms the reliability and accuracy of the computational setup used.

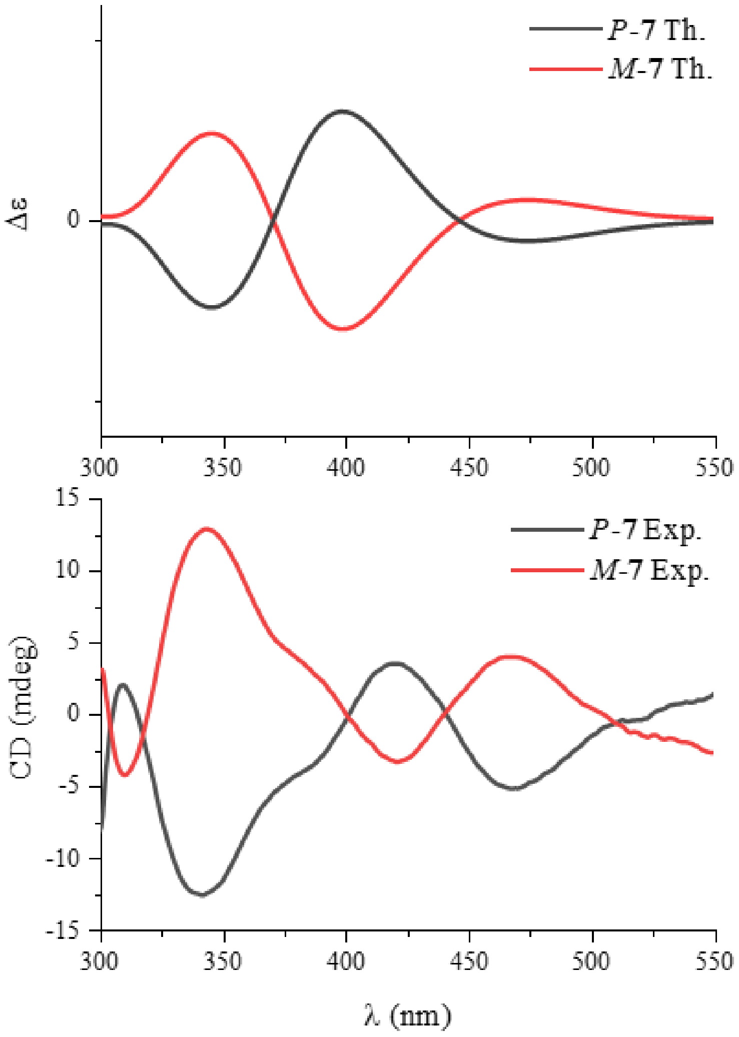

Figure S8. Computed Boltzmann-weighted electronic CD spectra (top) compared to experimental CD data (bottom) for the two catalyst enantiomers, *P*-**7** and *M*-**7**

Geometry of the Stannylated Carbene Intermediate

For the stannylated carbene intermediate derived from the stannylated diazoester **2** and the *P*-**7** catalyst at the Rh[O_3N] face of the complex, conformational sampling was performed using the CREST program. The simulations employed the GFN2-xTB semiempirical method and were carried out without any constraint.

As previously done for the conformational sampling of the catalyst itself, an in-house code was used to compare RMSDs across the conformational space obtained from the CREST calculation. This approach ultimately allowed us to select a small subset of diverse conformers of the stannylated carbene. The geometries of the selected conformers were refined using the B3LYP-D3(BJ) functional and Ahlrichs def2-SVP basis set with the RIJCOSX approximation. Implicit solvation effects at the CPCM

level were included by using the parameters for dichloromethane (DCM) to reproduce experimental reaction conditions as closely as possible. Free energies were obtained by adding thermal corrections computed at B3LYP-D3BJ/def2-SVP+RIJCOSX+CPCM(DCM) level of theory to electronic energies calculated at B3LYP-D3BJ/def2-TZVP(-f)+RIJCOSX+CPCM(DCM).

Stannylated Carbene Intermediate: Steric Maps

The most stable conformer was further analyzed using topographic steric maps, which allows more detailed characterization of the catalytic pocket (Fig.S7). These maps were generated using the web application SAMBVCA.[21]

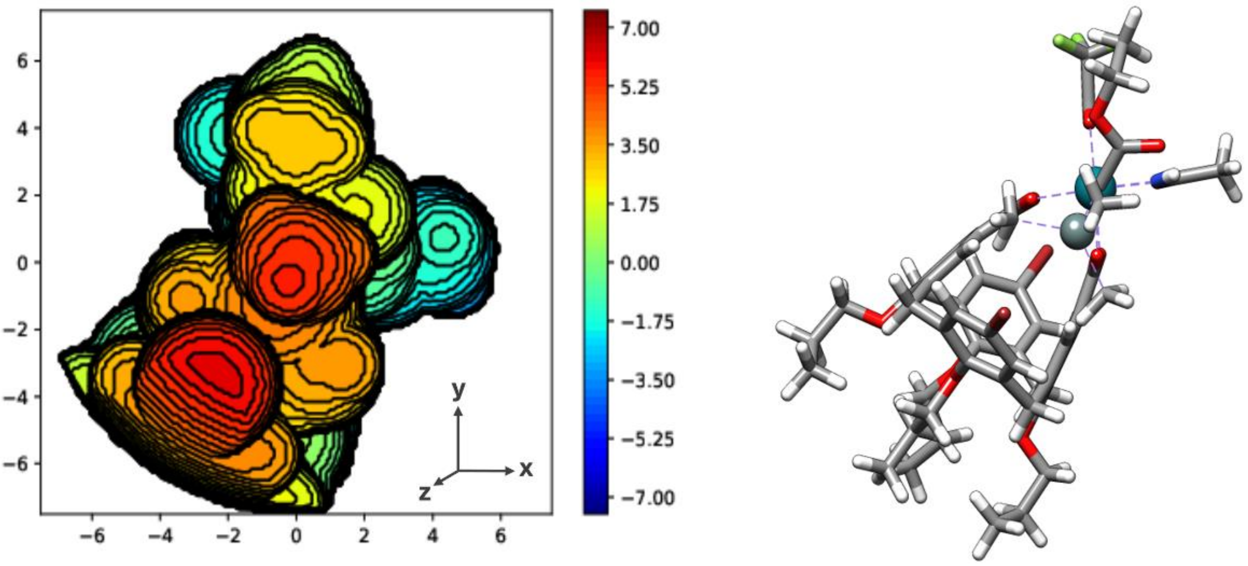

Figure S9. Topographic steric map of the stannylated carbene intermediate (left), whose structure is also reported (right).

Transition States

In order to gain insights into the factors controlling the stereochemical course of the reaction, the transition states leading to all four possible stereoisomeric stannylated cyclopropane derivatives were computed at B3LYP-D3BJ/def2-SVP+RIJCOSX+CPCM(DCM) level of theory. Free energies were obtained by adding thermal corrections computed at B3LYP-D3BJ/def2-SVP + RIJCOSX + CPCM(DCM) level of theory to the electronic energies computed at B3LYP-D3BJ/def2-TZVP(-f) + RIJCOSX + CPCM(DCM) level of theory.

The free energy barriers $\Delta G^{\#}$ relative to the isolated carbene and *p*-methoxystyrene reactants are reported in Table S1, while the corresponding relative activation free energies are shown in Table S2.

Table S1. Activation free energies associated with the pathways leading to each product configuration.

Product configuration	$\Delta G^{\#}$ (kcal/mol)
1*R*,2*R*	1.08
1*R*,2*S*	9.06
1*S*,2*R*	7.41
1*S*,2*S*	8.50

Table S2. Relative activation free energies for the pathways leading to the four diasteroisomers.

Product configuration	$\Delta\Delta G^{\#}$ (kcal/mol)
1*R*,2*R*	0.00
1*R*,2*S*	7.98
1*S*,2*R*	6.33
1*S*,2*S*	7.42

Cartesian Coordinates

***P*-7 lowest energy conformer structure**

Br	1.95122965655325	-6.02461999664171	-0.26584377639619
C	-0.10244512286453	-4.57346973605613	1.11537482146724
H	0.47070598404085	-4.81028685921093	2.01364347731091
C	0.33833391883216	-5.00123573466953	-0.13747382728343
C	-0.33671446671750	-4.64958911909136	-1.30806057494803
H	0.06213066152400	-4.93501614271795	-2.28330631524927
C	-2.60036602849041	3.77750127682940	-1.06922016114942
H	-2.43948052031431	4.22357574773890	-2.05239992979068
C	-2.17195613264049	4.43541091544610	0.08407005253001
C	-2.29433965345534	3.84745861327889	1.34418731147426
H	-1.89295517821916	4.34666979359788	2.22811117731007
Br	-1.32628163249786	6.14534382379732	-0.07347175427300
Rh	3.81757683474928	-0.32134995813066	-0.40540602213970
O	2.02550796443684	1.56323879468238	1.29432537378010
O	2.56951315674073	-0.61555868039813	1.24615559792414
O	1.56458305847128	1.52848372510879	-1.43732228838894
O	2.13326899532373	-0.64924342729454	-1.50395424450250
C	1.78110055268103	0.33522680243058	1.54401256170044
C	0.44463951162707	-0.00403671098692	2.10393436157043
C	0.05252859827399	-1.34576836892303	2.20407602341819
H	0.78066772554401	-2.12281101990180	1.97044385388676
C	-0.50064743056672	1.00602553185324	2.32374134710980
H	-0.20513710883102	2.04621993304267	2.18119272686705
C	-0.42904975066229	-1.39072856253937	-2.25471110916661
H	0.34371703010040	-2.15383253904350	-2.16390647801173
C	-0.07500974950576	-0.04775068567462	-2.08852540103708
C	1.30496586893430	0.30385831777895	-1.67423462103444
C	-1.06975069194333	0.93670207301333	-2.12026541533774
H	-0.79058798647940	1.97224866536764	-1.92759075698278
Rh	3.21374389899926	1.99191490079457	-0.33664676696337
N	4.89531221134376	0.13228622151002	-2.04587652486216
O	4.31358746820860	2.33392964094761	-2.00832550836204
O	5.45460223376678	0.12818348082713	0.77472726231071
O	4.90032896909591	2.32136084496624	0.81416360897546
C	5.77821446010824	1.66982007944305	-3.74866327531958
C	6.89101349668165	1.61802065583034	1.92424790498549
C	5.61620468397371	1.33122424277716	1.09880675796925
H	6.28943092954791	0.78437072843419	-4.14931223261966
H	5.12393392204508	2.09897336244368	-4.52313645883003
H	6.52276589757236	2.43596280494474	-3.48266246896106
C	4.95228901990080	1.34722075161663	-2.53273036653357
H	5.44736376922294	-0.57032566472211	-2.53306021071026
F	7.02459571258907	0.72723099866629	2.91149224994146
F	7.96626669138697	1.52308518185785	1.12694054800743
F	6.87101716688328	2.83904144869454	2.45519223310111
O	-3.48843555634273	-0.98063113049098	3.06047742490793
O	-3.14783502498767	-2.79243815317348	0.12555171112019
O	-4.05384518861156	-1.12381402431130	-2.65791545905687
O	-4.01079151775951	0.74446298706595	0.41728289621789

C	-1.26025425463356	-1.69409796556461	2.52945492008960
C	-2.19089614724904	-0.65954761440549	2.76835254449015
C	-1.82024448244129	0.69784968532061	2.65774163521687
C	-1.68348962297189	-3.15892305334605	2.52070077955245
H	-2.76905521482697	-3.21707367677840	2.65913193181274
H	-1.20693116472316	-3.69068742162633	3.35926302289117
C	-1.27400952098395	-3.80913091022803	1.21536699525563
C	-1.50456014032957	-3.88216035408801	-1.22775652714491
C	-1.98843652986842	-3.51683657737778	0.04194425272851
C	-2.11629817639764	-3.24707017774560	-2.46060775566518
H	-1.71479490692130	-3.73455480218014	-3.36252772415955
H	-3.20636132545794	-3.35484362547896	-2.48109098902285
C	-1.75809901917476	-1.76606726690740	-2.45434609827442
C	-2.41048331647556	0.60086324909135	-2.31624365658720
C	-2.74495736983842	-0.75894760222411	-2.49867031721076
C	-3.48083076811617	1.67927890277618	-2.19558603035742
H	-4.46370989854495	1.19992296126287	-2.13395802647255
H	-3.47480142832612	2.32319166026944	-3.08938930515178
C	-3.20958183653058	2.51988003489573	-0.96657695920912
C	-2.89807628330110	2.58969869646660	1.46709434540921
C	-3.40460426183908	1.96456271398214	0.31089662920814
C	-2.84150793686504	1.82145078356826	2.77409703817533
H	-2.54748221320004	2.50847264297403	3.58194254799460
H	-3.81796550072692	1.39999206472371	3.03757170986145
C	-3.79558305614683	-1.12491342344783	4.45328637386096
H	-3.10506543714534	-1.86129085805415	4.90552478548027
H	-3.63672596004716	-0.15824514132088	4.96512655879152
C	-5.23689712931571	-1.58067433483119	4.59211611897795
H	-5.89090265067100	-0.82712204423213	4.11957454150411
H	-5.48182356406241	-1.57616118721003	5.66815443223910
C	-5.50529455186890	-2.96214317485480	3.99578358342980
H	-4.86859733691665	-3.72823356600447	4.46891679373045
H	-6.55607460558578	-3.26033238626751	4.13770788211708
H	-5.29551013192541	-2.97429184598798	2.91697058486452
C	-4.33016337602301	-3.60489686108394	0.13384472962978
H	-4.34393992967724	-4.21858736281346	1.05447334647891
H	-4.30489463502221	-4.30342449382621	-0.72288131465644
C	-5.54453776729860	-2.70567334413310	0.05473550876003
H	-5.52074649636022	-2.02014194971033	0.91376607479618
H	-5.44443510239608	-2.08539967184659	-0.84770745410516
C	-6.85544102875671	-3.48742975034447	0.04009685809357
H	-6.97608970422538	-4.08875295560453	0.95677164360100
H	-7.71691739486429	-2.80491690553107	-0.02778846828935
H	-6.90488040254147	-4.17461746725296	-0.82074502788396
C	-4.57981629904016	-1.00380646187228	-3.98690207901565
H	-4.61375457955802	0.06190705351996	-4.27567284150860
H	-3.90274354510608	-1.52245449142120	-4.69108063946675
C	-5.96743624108049	-1.61821078825225	-4.02379746536046
H	-6.40529619276617	-1.38193320790334	-5.00889661413032
H	-6.59596268262450	-1.10945538343796	-3.27209213512735
C	-5.97946025452488	-3.12926613829854	-3.79396917834368
H	-5.57034581960440	-3.38492691291668	-2.80598934592819

H	-7.00298296832303	-3.53212634792937	-3.84853493434551
H	-5.36989444317973	-3.64830036722063	-4.55248112433925
C	-5.34445486936474	0.75020533444784	0.95146518277402
H	-5.42761975026866	-0.15510115040185	1.56699700130588
H	-5.47564085076756	1.61822558925102	1.61941399449828
C	-6.39974416198364	0.77062577053089	-0.14549124437423
H	-6.33570729731591	1.72644238666969	-0.69316577803290
H	-6.16508700940714	-0.02608491201086	-0.86804079878378
C	-7.80705432336971	0.57693833401652	0.41801996366656
H	-7.89269960447683	-0.38959861064964	0.94219956081621
H	-8.06670904258051	1.37076964805510	1.13850514291768
H	-8.56289847983315	0.59180929359341	-0.38267914544856

carbene lowest energy conformer structure

Br	0.95835759027937	-7.27067922954170	-0.28247435289984
C	-0.47259776536081	-5.20561345230753	1.11739177523384
H	0.08911980906809	-5.53675542073704	1.99203382653622
C	-0.27967759268404	-5.81554881391592	-0.12389332439548
C	-0.92506137386558	-5.35816996137435	-1.27361234005167
H	-0.71915325388907	-5.81186497691532	-2.24427992597397
C	-2.32669355768871	3.38409101757405	-1.44398275134557
H	-2.13770177629101	3.74777854878860	-2.45566326135772
C	-1.77965689320098	4.04239977444960	-0.34270671776488
C	-1.93238481223197	3.54556917118330	0.95301119829072
H	-1.42906623473577	4.02715278636991	1.79351373446053
Br	-0.71444501913860	5.60907518728161	-0.62353198108608
Rh	3.85805177219569	-0.66150499815974	-1.10019474875919
O	1.97221270878087	1.25395001526624	0.53751477787769
O	2.60437767637657	-0.90412221652407	0.59350345484786
O	1.50205050637170	1.04859062396902	-2.19198922252902
O	2.11150798965763	-1.12195352881102	-2.11461948932589
C	1.80475571289219	0.04273872034517	0.88271990744557
C	0.53393368413985	-0.28255985726572	1.59459482784729
C	0.14645406348175	-1.60793387035795	1.83506653815003
H	0.83620705626516	-2.41240353565913	1.58539671698214
C	-0.37125238115895	0.75186503430285	1.85702109551582
H	-0.07927637798934	1.77177417248086	1.61051630284783
C	-0.61679637840398	-1.89449684793522	-2.46691715644994
H	0.11596695853779	-2.69518423302734	-2.38181115024716
C	-0.18065562453655	-0.56311331927252	-2.48014878002692
C	1.24775006743109	-0.19111177121419	-2.27053200383981
C	-1.12976113098921	0.46739959245487	-2.51548797952824
H	-0.77969007804312	1.49692545650941	-2.46317829318803
Rh	3.10512644890529	1.70079904185824	-1.11782812636346
N	4.88104630495694	-0.12586592320681	-2.75670602924894
O	4.19044749079335	2.04808923484918	-2.80970480368723
O	5.46525628526067	-0.00749915417331	0.02452258501263
O	4.78306576462040	2.15337106096395	0.02047282206532
C	5.76425857600738	1.38926679673588	-4.46017949046369
C	6.84811541646383	1.59016287991381	1.07164974482199
C	5.55978954825366	1.21882626310084	0.30173350715970
H	6.31852236970545	0.50833027095557	-4.80955118539269

H	5.13214295556523	1.77357401307247	-5.27537832995977
H	6.47371704294944	2.18542358432668	-4.18603661609619
C	4.89286649960958	1.08360947311150	-3.26879307664966
H	5.53258814183877	-0.80377561565395	-3.14947782065326
F	7.07056540974532	0.72714745257272	2.07159331235758
F	7.89907851628813	1.53879070029046	0.23695058206667
F	6.78148785452838	2.81714179378576	1.58332095531890
O	-3.26321221888414	-1.12424638689967	3.08646954123545
O	-3.10265707081443	-2.83025014497948	0.21889348640743
O	-4.25098521198825	-1.43842453164634	-2.56595680672034
O	-4.13486763799004	0.69986571175108	0.27373403591936
C	-1.12152002494991	-1.90873407838987	2.34243783656782
C	-2.00999060966449	-0.84359352077647	2.61411445287607
C	-1.64814896592599	0.49571107507855	2.35712306735446
C	-1.55285717876722	-3.36016453873485	2.51758423929617
H	-2.59966316855347	-3.37827123153792	2.84602152162552
H	-0.95116087200017	-3.83494903739601	3.30801359936985
C	-1.38674515151870	-4.14901627714878	1.23272040065425
C	-1.83783777863791	-4.30131604543558	-1.17825503223216
C	-2.11223229481151	-3.76499181455694	0.09172914955120
C	-2.43972848182005	-3.65009907525802	-2.40623447880456
H	-2.12883606704735	-4.21343648627803	-3.29954998013391
H	-3.53596461448936	-3.67302980711213	-2.36795813006782
C	-1.98035126024659	-2.20302226682918	-2.51540317044445
C	-2.49827315462577	0.20101450925958	-2.54243377452476
C	-2.91523879460918	-1.14616181513483	-2.56427668858756
C	-3.49322379439995	1.35179676910161	-2.42257286612479
H	-4.49891052588455	0.94273356662833	-2.28309806549451
H	-3.49284378551352	1.94933483980093	-3.34766594470526
C	-3.09705236223804	2.22797392279772	-1.25202092883786
C	-2.69214058651107	2.38831405299015	1.16375119972674
C	-3.33215686690178	1.78758951079635	0.06233943772960
C	-2.64047844008397	1.64871646967064	2.48741186160422
H	-2.31361164797702	2.33831710744321	3.28103754908753
H	-3.62179665119898	1.25360545096618	2.77196867746312
C	-3.42007711844979	-1.05561477794939	4.51087783324128
H	-2.67401870256203	-1.71738447774884	4.98925024374542
H	-3.22266086510148	-0.02377140349641	4.85274717215859
C	-4.83126536944077	-1.48459470114900	4.86767150880171
H	-5.54201692349682	-0.80740095257133	4.36213024149515
H	-4.96048979257937	-1.32190063042906	5.95147674700236
C	-5.13956819513457	-2.93854778376076	4.51129327425696
H	-4.44711679825333	-3.62686411141341	5.02420514767925
H	-6.16620515672557	-3.21084389267300	4.80281582659847
H	-5.04044115363067	-3.10939488343983	3.42989579846746
C	-4.39074779973339	-3.42959619585387	0.44182116084095
H	-4.34104362278457	-4.07055978325537	1.34148566785740
H	-4.64214595570871	-4.08396665665097	-0.41282392675236
C	-5.41703163084794	-2.33437515864235	0.61359941593279
H	-5.09033288168501	-1.70903733733649	1.45603663076615
H	-5.39263676071969	-1.70387393010388	-0.28674883753391
C	-6.82474988042392	-2.87252229479779	0.85694477736013

H	-6.87128598647303	-3.48622298663493	1.77187367357083
H	-7.54210576084895	-2.04470160724939	0.97363036772812
H	-7.17077942254955	-3.49660413737883	0.01656829130972
C	-4.89089993546843	-1.44022682781088	-3.84984710403082
H	-4.81010871193705	-0.43672580018394	-4.30445696932558
H	-4.36687708057706	-2.15406661553916	-4.51276051338778
C	-6.34382109806898	-1.83770265956232	-3.66702228274700
H	-6.83930989798068	-1.73733161110024	-4.64791296373647
H	-6.82553423848029	-1.10576106525344	-2.99532210506344
C	-6.52607942765508	-3.25439919387171	-3.12445306920436
H	-6.06239383681122	-3.35900913664987	-2.13329180422212
H	-7.59297899734693	-3.50967422436356	-3.02640345917996
H	-6.05878367692367	-3.99692006961009	-3.79282091206980
C	-5.43967092723156	1.00911828733487	0.79820140656121
H	-5.70351277726947	0.19349869579388	1.48449736879905
H	-5.38620322413520	1.93856613606842	1.39019052395518
C	-6.48522458392158	1.14437303709959	-0.29953899089815
H	-6.21151502941618	1.98488543755940	-0.95959545552259
H	-6.46603541356619	0.23316064580362	-0.91975476761638
C	-7.88458765548523	1.36001731728535	0.27567640173553
H	-8.18307311248824	0.51479858375166	0.91851105073352
H	-7.93158752077272	2.27684289660749	0.88704338136335
H	-8.63386025080962	1.45288575492731	-0.52584169756272
C	4.58556293537766	-2.45737317409990	-1.16864864562839
C	6.03551819994667	-2.59892450187180	-1.21748266902208
H	5.64904671737404	-5.66843511752892	-2.34104162121193
O	6.59909926033835	-2.93550530598573	-0.06426244295113
C	8.04536945723241	-3.08706528656746	-0.02498054577905
C	8.72368627789697	-1.75289334308424	0.21093682997049
H	8.22037578259655	-3.78779001379538	0.80198962969801
H	8.37132888182641	-3.55242679320441	-0.96560587416549
H	9.81277458933367	-1.90418756873596	0.27911496050590
H	8.52135330299325	-1.05528299870216	-0.61456081146222
H	8.37532856276284	-1.29491193137832	1.14762041157693
Sn	3.51530635877072	-4.39995957104443	-1.18602201434932
C	2.80220072040146	-4.31955431503327	0.85194360155822
C	2.18987910265192	-4.39374505664826	-2.88671197916677
C	5.09576768475415	-5.86535942086895	-1.41071411711806
H	3.44290831815188	-3.62590887270101	1.41222159843565
H	1.77009787858716	-3.95862729356142	0.85736273087145
H	2.85057810405729	-5.32712711873326	1.28474242269203
H	2.54770061275386	-5.14343427934675	-3.60588477421059
H	2.21014061679521	-3.39005703798840	-3.32844753407286
H	1.17071794712911	-4.63820457219432	-2.56847297454211
H	4.65678606189742	-6.87309769167848	-1.45372506303649
H	5.78388043894611	-5.80048362977889	-0.55552744814638
O	6.61923160821105	-2.45028385984952	-2.28490262089783

***p*-methoxystyrene**

C	3.42945909557290	-2.15098800454920	2.43739420764631
C	3.61401739883049	-1.78271893547814	1.15890216606564

C	3.02881348615266	-1.29738988644144	3.56618365384212
C	2.78005253178750	0.08794254774288	3.43929518359295
C	2.40261448635100	0.85511851231411	4.53209217909931
C	2.25738596454378	0.26624252979114	5.80462724101108
C	2.50028979383772	-1.10720259262195	5.95469264697938
C	2.87975753850693	-1.86536359166132	4.84243117395452
O	1.88484578416980	1.09487174484645	6.80919548894375
C	1.71822065197865	0.56155205371494	8.11187204526572
H	0.93482210145594	-0.21573062984551	8.13298247926013
H	2.65922889752586	0.13075821590134	8.49582576422234
H	1.41493205875380	1.39808263524195	8.75489889978891
H	2.39844005414764	-1.59441040527882	6.92430079046954
H	2.21063922698175	1.92574851209922	4.43037708661727
H	2.88420717027788	0.57414561725771	2.46657729526151
H	3.06624124322143	-2.93530189478750	4.97260586289033
H	3.58829655233667	-3.20390007656570	2.70082999013806
H	3.47497244044974	-0.75237683031255	0.81744860350789
H	3.91424342953700	-2.51418764316399	0.40389990297160

transition state leading to the 1R,2R product

Br	2.32346916692544	-5.18535522260369	-0.08687248338884
C	0.39709316793068	-3.39478188189743	1.06002501024740
H	0.95291952550324	-3.54342081014894	1.98422103654149
C	0.81462960011681	-4.00468723704371	-0.12313380517910
C	0.17885670007937	-3.74756378181353	-1.33766872891874
H	0.54803401546600	-4.19269652900119	-2.26342527313796
C	-3.19025634826445	4.47562293861506	-2.40505607357810
H	-3.07808614628179	4.86204003923197	-3.41979593731803
C	-2.97405210303728	5.30496899110448	-1.30381773547212
C	-3.02825813640677	4.81095184583577	-0.00027938719768
H	-2.79916943404279	5.46004675159769	0.84687545979144
Br	-2.52448338603913	7.14419327296571	-1.59115557770069
Rh	3.87526513639766	1.51649371106724	-1.27097468658670
O	1.59630227692337	3.29590920133808	0.02447635291926
O	2.55706683225725	1.29070430013519	0.33951049286464
O	1.46032740902048	2.82984881400722	-2.69099066209807
O	2.34068421447134	0.77340355396720	-2.38835956039535
C	1.58685340915234	2.10923695669888	0.47128052486148
C	0.34664315167311	1.61046199721769	1.11878668294479
C	0.22955010429783	0.26297321970689	1.48583434251989
H	1.07601507122900	-0.40657044695782	1.34692166826049
C	-0.77164971432489	2.44890597578368	1.20791938175968
H	-0.68786094587718	3.47624411217949	0.85273890770951
C	-0.12149058775774	-0.44075252025924	-2.82506771365441
H	0.73897592473115	-1.06742219314004	-2.58613699321396
C	0.05678840193542	0.94377060996469	-2.95864706476011
C	1.37938172637530	1.56568063923544	-2.68632581537219
C	-1.05862385006600	1.76422133041982	-3.17373665456300
H	-0.92202964200704	2.84578443705775	-3.19907921673805
Rh	2.87297933402937	3.77109124061997	-1.52244711987459
N	4.91174577868952	2.01461772751979	-2.93173877817306
O	4.10297889558321	4.14016180992961	-3.10624281911033

O	5.29147452826823	2.41266198375568	-0.06246819626863
O	4.34816834824996	4.46538473561447	-0.22688071504158
C	5.65684438572568	3.45123482285666	-4.76396217906232
C	6.21022334264960	4.15776150350872	1.23044248358038
C	5.18468189041146	3.64077538020306	0.19584265744384
H	6.28592006176568	4.33785428973462	-4.59102079709995
H	6.29207877804058	2.59834938306333	-5.03661071059160
H	4.97547086574977	3.68431632539914	-5.59690991397246
C	4.84160234222331	3.18366253369561	-3.52496118534346
H	5.47766465315214	1.29553751634109	-3.37862408305899
F	5.80636183912547	3.80687015412646	2.46265454955610
F	7.41909081349135	3.61913062041495	1.02588200466297
F	6.32863111820885	5.48339649391938	1.19021689276414
O	-3.27331571744472	0.13234882647894	2.53437594294844
O	-2.54133999000446	-1.59242088478094	-0.18261019495854
O	-3.73081433119136	-0.73593788348979	-3.35784095888366
O	-3.95187854858448	1.33354550055801	-0.67572407375553
C	-0.97259242668879	-0.24535493976719	1.97801136264587
C	-2.07697121210809	0.62469720400181	2.08816448370318
C	-1.99678161006005	1.97020084616554	1.67327501356696
C	-1.08938131735998	-1.73219929747150	2.27598528648616
H	-2.10909512208026	-1.95397820752961	2.61469481280652
H	-0.40671482395046	-1.99971679996643	3.09722859842799
C	-0.72054241350528	-2.55164576816263	1.05042976366773
C	-0.94536499580994	-2.91174720697612	-1.37181541135297
C	-1.42456875352005	-2.37627450391811	-0.15990147295435
C	-1.57595887600914	-2.50445560106200	-2.68877624324318
H	-1.11072220999613	-3.08577995189107	-3.50029524599264
H	-2.64820709203218	-2.73124785385976	-2.69677103454985
C	-1.38825311561912	-1.01678211534536	-2.94429912328917
C	-2.33943379472709	1.22382574846771	-3.29123148566566
C	-2.48879432437270	-0.17676491843555	-3.21505295866378
C	-3.54014754573351	2.15654700044793	-3.37050690284776
H	-4.46163816119972	1.56357431756511	-3.37451835201238
H	-3.51468221812231	2.72658719650104	-4.31179555015839
C	-3.51135045207098	3.12720478535807	-2.20523835264604
C	-3.35264283545364	3.46567411097541	0.22219869325601
C	-3.64558782121182	2.64804251124185	-0.88601899509760
C	-3.23187287740750	2.85782039165803	1.60488180078352
H	-3.15939183917794	3.66649074368322	2.34873711918653
H	-4.11478908069822	2.25768677025914	1.85079258637936
C	-3.51536796488690	0.26108089869875	3.93906105731154
H	-2.69824005053803	-0.23002065594893	4.49999515546350
H	-3.51423575064685	1.33102284812978	4.21960001138909
C	-4.85222948767722	-0.38199575970439	4.25731358181693
H	-4.82566831263990	-1.43056584578101	3.91414321239179
H	-5.63554899100287	0.12345377322479	3.66682826348054
C	-5.18507499931649	-0.31639362681971	5.74709534102138
H	-5.23067665768637	0.72695055185503	6.10159555490574
H	-6.15990429249327	-0.78376631953892	5.95580445711871
H	-4.42513532739042	-0.84054073849246	6.35045468088676
C	-3.73757590806065	-2.15026043093179	0.38498192273477

H	-4.29871174865124	-1.30018657980127	0.79152948268073
H	-3.48115843577646	-2.81050276578512	1.23019771728567
C	-4.56228972390336	-2.90680243804665	-0.64366631840275
H	-4.74097267879545	-2.23733277427551	-1.49862280200932
H	-3.97620650578373	-3.75968299615212	-1.02716552555905
C	-5.88498949113136	-3.40051641699939	-0.06080160443228
H	-6.48973268776834	-2.56007038152327	0.31853424218820
H	-6.48194091771804	-3.92704007682349	-0.82189516144492
H	-5.72048046878348	-4.09661464940072	0.77877170371583
C	-4.17027171469762	-0.87898091522754	-4.71920077726876
H	-4.24779244667344	0.11968670558155	-5.18381320801998
H	-3.41112852451401	-1.45265566589052	-5.28260284760450
C	-5.51230668362415	-1.58662558133200	-4.74693483536924
H	-5.91228302873639	-1.48323564688599	-5.77034655987673
H	-6.20727281979800	-1.04089187536524	-4.08474657450947
C	-5.45105718970341	-3.06456009459441	-4.36247553343152
H	-4.77127249751724	-3.61649566504751	-5.03298266626154
H	-5.08690279975742	-3.19825427649093	-3.33423638150202
H	-6.44569561313027	-3.53319058127070	-4.42996455595549
C	-5.31419231846426	0.93337212413382	-0.88714042263459
H	-5.76269834636768	1.53908192279532	-1.69263266017891
H	-5.26944891704523	-0.10543886729946	-1.24109868933328
C	-6.15533210082647	1.04359290742292	0.37663991587952
H	-5.65687992767789	0.48153166802509	1.18279523606848
H	-6.18947083637320	2.09814266946813	0.69976231730009
C	-7.57327184456924	0.51754997857212	0.15649164963654
H	-7.55986549689518	-0.54154665079332	-0.15068007842223
H	-8.17315228466105	0.59213315303719	1.07693916026884
H	-8.09541176086867	1.08553483263018	-0.63175668822589
C	4.81435559943012	-0.16636929116036	-1.15466434800743
C	4.66713252735079	-1.11168283933635	-2.25296533144066
H	6.21538514723220	-0.54384869460199	2.84901075004076
O	3.81379918171686	-2.10789873983008	-2.06910831744298
C	3.67643565621372	-3.09558097599477	-3.12709909113003
C	4.77594797369619	-4.13551383805529	-3.05066609006871
H	2.68830210235061	-3.53627760817787	-2.94874889922198
H	3.66963033237800	-2.57257025407403	-4.09355199898417
H	4.78285610139411	-4.61944717556309	-2.06252516534386
H	4.60043941644948	-4.90934116990253	-3.81480453037689
H	5.76154926875068	-3.68338971015245	-3.23600779904828
Sn	6.59583259428511	-0.43657291255267	0.14635919765877
C	7.01869354931848	-2.56251938161339	0.05077289937686
C	8.06276364037086	0.69304214920047	-0.98562528188599
C	6.26725051684403	0.29904823114562	2.14714856768580
H	8.01211073208615	-2.75210523793125	0.48455930974821
H	6.25819685364829	-3.12051034719540	0.61486333478840
H	7.01342562106604	-2.89330736151844	-0.99818314664596
H	7.90800273138099	1.76048359865513	-0.78362811264274
H	7.92416642604840	0.48900283810262	-2.05684782369477
H	9.07440074691214	0.38969361844854	-0.67835232489313
H	5.32668605273022	0.85904204103081	2.16944071144912
H	7.09274892626033	0.97319495374249	2.41586465009148

O	5.33427493861336	-0.92139014489397	-3.26506628398683
C	3.49849214671440	-2.13363785160535	2.44980168996842
C	3.47811197200427	-1.79937077275275	1.14757036882113
C	3.11871562187181	-1.28393929963498	3.58444477786253
C	2.89692340522826	0.10585360695582	3.46155072370913
C	2.49513999736149	0.86948047365104	4.54753835054377
C	2.29338634157131	0.27204701696609	5.80686496662496
C	2.52378976329849	-1.10438036010844	5.95750577355376
C	2.93751490769781	-1.85875922395647	4.85506784035271
O	1.88549383312716	1.09457534126620	6.80320870878552
C	1.64953908787863	0.54844954874139	8.08929547352847
H	0.86383446540225	-0.22628618429547	8.05952031419878
H	2.56727620985925	0.11020215854518	8.51876986570979
H	1.31541578600487	1.37906977947076	8.72471749162128
H	2.38509520094622	-1.59525919937254	6.92064600550737
H	2.31571062127392	1.94194293736926	4.44371572020184
H	3.03374049824563	0.59167054236467	2.49650185575889
H	3.11632775294292	-2.92987793428774	4.98601846608810
H	3.77881693855902	-3.16034143663405	2.71533350551400
H	3.15496304193785	-0.81521996508956	0.80617965438223
H	3.73319231449359	-2.53132729938943	0.38218110879569

transition state leading to the 1*R*,2*S* product

Br	18.35049799012523	-31.92642766898958	-3.36580664897407
C	17.29813791577862	-29.69169406414791	-1.91754155568237
H	17.79739518344985	-30.13907427770488	-1.05636917586844
C	17.42380262439591	-30.26552288287088	-3.18189993333166
C	16.89977150658644	-29.64303240652807	-4.31505154684572
H	17.08357130949329	-30.05833921205241	-5.30679660228812
C	14.90520258365694	-20.82122567899202	-3.95512358027410
H	15.02368332326304	-20.30722315230820	-4.91075955426406
C	15.34233788973998	-20.22502863387397	-2.77147956905433
C	15.28390273085024	-20.90108237553885	-1.55309555188020
H	15.68495074275060	-20.44293806563407	-0.64709999255675
Br	16.11397595931859	-18.47342107780728	-2.83462257992814
Rh	21.58401605238647	-24.30220808151898	-3.85337569952656
O	19.54252022910148	-22.79899244424485	-2.01173826876148
O	20.31624402315050	-24.89668333916768	-2.25416964395961
O	19.08331558613344	-22.74631735306283	-4.75773579403218
O	19.93314219949705	-24.82334773563934	-4.97574857391381
C	19.45959930207178	-24.05414974602197	-1.83242163545857
C	18.22660917326898	-24.55113781904413	-1.16070843297871
C	17.98060796035807	-25.91930511630937	-0.97647177318266
H	18.73839164282436	-26.64845643340871	-1.26226784118373
C	17.21429834700800	-23.62546514024374	-0.87242357646726
H	17.39185719948332	-22.57401029085906	-1.09401407424284
C	17.27154326029613	-25.84669091371854	-5.31559743274942
H	18.07357963901861	-26.58071789871794	-5.24520419960853
C	17.58438233316037	-24.47983896302581	-5.25912233807909
C	18.96565738561506	-23.98459428627480	-5.00361510885054
C	16.54579948758231	-23.53890047723156	-5.26687581771573
H	16.79468777862728	-22.48587552124428	-5.14173397424223

Rh	20.59893912726358	-22.03797336046695	-3.59453365820038
N	22.53544248741578	-23.44429312745929	-5.41621672007641
O	21.62857567149542	-21.35918130190180	-5.21176981784685
O	23.06083694556754	-23.59177457657250	-2.57073939717186
O	22.18808383349587	-21.51320146376658	-2.37656792930320
C	23.19777615591313	-21.67564504703133	-6.96464772138890
C	24.25918248729499	-21.99821124951693	-1.30148718680453
C	23.03700619579514	-22.40089659422327	-2.16035789724185
H	23.81114178134665	-20.82361702193070	-6.63315041588689
H	23.84576910428710	-22.44589231668133	-7.40302378914329
H	22.49915574740259	-21.30432267513681	-7.73031980987261
C	22.40580186928179	-22.19346363983739	-5.79109472160039
H	23.21602805965984	-24.00967253320118	-5.92392460805787
F	24.46433873074787	-22.87924072619499	-0.31346180238728
F	25.36047882941428	-21.98083620103064	-2.07187701397497
F	24.10778106699278	-20.79190158355892	-0.76282009649768
O	14.56557008199020	-25.83090411255582	0.34042455014355
O	14.99607242778155	-26.96042739902324	-2.72714112291662
O	13.62431416944578	-25.70635902212135	-5.63853223438269
O	13.67386646428608	-24.00996878031317	-2.62711101461063
C	16.75404016352324	-26.36267984650102	-0.47489922064892
C	15.77249351360359	-25.40491348653779	-0.14242384479446
C	15.97508521418569	-24.03124662318528	-0.37969164827111
C	16.45272049008438	-27.84917370673430	-0.39479531888456
H	15.46063866593303	-27.98505755860692	0.05716064389065
H	17.17683847880015	-28.34016609971141	0.27317395730265
C	16.53065147240233	-28.53112991774282	-1.75214763448094
C	16.13155657228451	-28.48022776635191	-4.17802403303322
C	15.87190206997925	-27.99640943494482	-2.87968304814907
C	15.59502538655998	-27.75506022218649	-5.39930245588923
H	16.00216849847203	-28.24664972560754	-6.29684785811722
H	14.50319618097927	-27.85132883235411	-5.44982032832733
C	15.94416713669803	-26.27551321138289	-5.42337678271145
C	15.21200445429283	-23.92740704733649	-5.36832199204056
C	14.92417807991099	-25.30089519261939	-5.49315963824649
C	14.11720043297814	-22.87941851081161	-5.20754337158068
H	13.13835808542639	-23.37121794606098	-5.21021826094533
H	14.13600430342531	-22.17777969772734	-6.05552127322427
C	14.35819123140905	-22.10992860347100	-3.92157999654614
C	14.73333844991535	-22.18909788439605	-1.49541653724717
C	14.22023354943244	-22.75795309481628	-2.67663710001006
C	14.84304455089918	-23.02098669931099	-0.23411601054153
H	15.03299630369608	-22.35765735528571	0.62425206254112
H	13.90922198475246	-23.56002405399326	-0.04083204466768
C	14.39338672848682	-25.75449597092028	1.75915811912776
H	15.14911189928192	-26.39288729331673	2.25404684978074
H	14.55908439558855	-24.71629862663768	2.10121344723762
C	12.98739233165255	-26.21626329708903	2.09434515094697
H	12.85279263090744	-27.23954145250811	1.70344188320087
H	12.27050398609110	-25.57556470097022	1.55262991659923
C	12.70600223977979	-26.17804967412701	3.59555615656225
H	12.81428999000473	-25.15670177383621	3.99736031531416

H	11.68151922949662	-26.51708173763097	3.81420706625832
H	13.40088430788209	-26.83040771254182	4.15040455582886
C	13.71951317783548	-27.30696598623244	-2.16267714460571
H	13.33440931495555	-26.37650157275142	-1.73462630096012
H	13.85870327330012	-28.01599279811622	-1.33084352649439
C	12.75504668453443	-27.88008971060246	-3.18685124237466
H	12.66800840006244	-27.16548064392267	-4.01769558308407
H	13.17399729103880	-28.81001324945587	-3.60914345956170
C	11.38407637785194	-28.15804557943137	-2.57184071530862
H	10.94607002840714	-27.23873395922848	-2.14886403896316
H	10.68195581325556	-28.54650639246743	-3.32633684328084
H	11.44779237428249	-28.89999881139388	-1.75809953550646
C	13.10064525396712	-25.57991816002676	-6.97114323521974
H	13.08068166969675	-24.51329081385538	-7.25614640055646
H	13.77722043479026	-26.10200703143689	-7.67284138622701
C	11.70567628309993	-26.17482618017438	-7.02529187908947
H	11.27153198647800	-25.89114624967887	-7.99951215526396
H	11.08552393738740	-25.68486586243903	-6.25413164167129
C	11.66474143227337	-27.69414969010289	-6.86148371412212
H	12.27844106801339	-28.18904634007948	-7.63269458491028
H	12.04978279403310	-28.00316251133051	-5.87942011381436
H	10.63506648725683	-28.07449079308051	-6.95358852399311
C	12.25051654980992	-24.09900549807330	-2.80169050217674
H	11.90346860336941	-23.28664028189868	-3.46191832649574
H	12.06691423901494	-25.05038942814094	-3.32055074259302
C	11.50410352400195	-24.04399080875218	-1.47625632675557
H	11.88191182454931	-24.84454256596214	-0.81900353442873
H	11.73219801766146	-23.08814383000200	-0.97432672779679
C	9.99476840038420	-24.18664665496333	-1.66889208745855
H	9.74355962975521	-25.14576627269724	-2.15186045151877
H	9.46578885815235	-24.14914227060721	-0.70383728805913
H	9.59332627499765	-23.37908505185833	-2.30377264345721
C	22.66206036969397	-25.91515965476367	-4.06535372298998
C	23.08762470825835	-26.40937207447352	-5.36989018228293
H	23.15557006062820	-26.87479934245359	0.16589614673934
O	22.48492463150666	-27.49483232391722	-5.81685653581663
C	22.94105860455689	-28.06749843825486	-7.07239592919331
C	24.10075963477568	-29.01537058023239	-6.84224935608616
H	22.06123903996932	-28.59440299331168	-7.45992580847064
H	23.20855998145637	-27.24893323350657	-7.75423573120594
H	23.81410507400311	-29.81129633423231	-6.13913657231789
H	24.38759070977076	-29.48173224321011	-7.79812510947754
H	24.97559660941440	-28.48117083814672	-6.44162848508348
Sn	24.01553943197449	-26.53060683508184	-2.39992252108445
C	24.43726587695095	-28.64090454849296	-2.66773293645184
C	25.82035680297239	-25.44850666293959	-2.93652389721588
C	23.11680040394768	-26.01021109901103	-0.51048138012679
H	25.50492492210750	-28.80739395140592	-2.46067743830387
H	23.82497238131036	-29.23030270089953	-1.97342366202399
H	24.21260229538440	-28.94283034671343	-3.69946478464541
H	25.71847453578903	-24.38869281647379	-2.67638452130750
H	25.96920778001779	-25.55217817419784	-4.02075060941484

H	26.67229430216310	-25.89325427558412	-2.40085465307010
H	22.07402946175259	-25.72607161932946	-0.70108701643630
H	23.65867777498481	-25.15900395558003	-0.08063468734261
O	23.97975927952039	-25.80353588095148	-5.96276743104816
O	21.00654290444614	-31.48143015563128	-7.17573241355432
C	21.86627411415116	-32.60311565359646	-7.27605674346876
C	20.95023819100143	-30.80110912199115	-6.00234076969069
C	20.10482103896794	-29.67987420654791	-5.97283046488921
C	20.03145442755426	-28.89672325971475	-4.83051576884067
C	20.76059105909557	-29.21222381673535	-3.66955542360412
C	21.58118187012369	-30.35044686971241	-3.70879245201637
C	21.69297736034318	-31.13426621056274	-4.85770397037642
H	22.91957505214802	-32.32102627988464	-7.10498812489298
H	21.58680122847752	-33.39366882305254	-6.55824069942031
H	19.52915899850009	-29.43233041002278	-6.86733758257966
H	22.35742733690979	-31.99794955336883	-4.85188182391204
H	21.75775314546310	-32.98936256792446	-8.29807008782120
H	22.16542268025442	-30.62872878919373	-2.83038424140903
H	19.39172295697593	-28.01454829790931	-4.82435205477449
C	20.62980228509444	-28.34787312253830	-2.49005194592765
C	20.96903219713793	-28.64807542528477	-1.22531638901147
H	21.38571472274321	-29.62096945634727	-0.94764639468156
H	20.82855330568497	-27.92118865421117	-0.42249874481677
H	20.19691584302487	-27.36102985774585	-2.68776826700219

transition state leading to the 1*S*,2*R* product

Br	2.75887455054807	-12.87851112704804	-2.90073641340719
C	1.10771810734311	-10.88728430306685	-1.65755689520179
H	1.72632360883532	-11.06308205896400	-0.77718540874205
C	1.35343086246147	-11.57827785971927	-2.84397665973914
C	0.63302277931113	-11.30518590274919	-4.00693007777987
H	0.87106760426240	-11.81735852524541	-4.94084094618451
C	-2.29071490744904	-2.89563535264064	-5.06815995946567
H	-2.25251774382184	-2.55278476473803	-6.10382645159974
C	-1.88433787335757	-2.05668276899339	-4.02943245239694
C	-1.83838554544500	-2.50499578095066	-2.70954494273583
H	-1.46378521463582	-1.85392655546687	-1.91759221257585
Br	-1.30552135219200	-0.27687051718479	-4.43527153229280
Rh	4.69119160857021	-6.04896081975475	-4.42249178857118
O	2.66702256649251	-4.17992274686888	-2.90822380319905
O	3.61332200045153	-6.19751910513266	-2.58909044875809
O	2.11085194509780	-4.74244623799497	-5.56832512101911
O	2.96883639785283	-6.80475257942053	-5.24771867664032
C	2.67533768281114	-5.35329248388639	-2.42031560590750
C	1.45086837345943	-5.78967594231039	-1.68607207222943
C	1.29411864023253	-7.12388215917447	-1.28506130631197
H	2.10821502441240	-7.82512910077742	-1.45390194345177
C	0.37197277439970	-4.90463922762772	-1.55864892277129
H	0.47797654455356	-3.88928827916766	-1.94090405960858
C	0.42110597020937	-7.95982559444415	-5.51568380317133
H	1.26473754505239	-8.62153564314918	-5.31329815585230
C	0.65121047666414	-6.58799333402393	-5.68627617082684

C	2.00786163208060	-6.00345735031730	-5.51086477027754
C	-0.43755795099908	-5.72380091319427	-5.85763197330025
H	-0.25250697530432	-4.65129564750792	-5.91366392807603
Rh	3.68498111033206	-3.79542641719908	-4.66667136301030
N	5.42489351970381	-5.72620386304322	-6.25979715642915
O	4.63995888723849	-3.59268268994701	-6.46399425716552
O	6.27908368900693	-5.06692203936796	-3.51920249939107
O	5.34220410458386	-3.01214308058150	-3.69818796958723
C	5.73145310160150	-4.51398866040282	-8.36009939525315
C	7.51619521951064	-3.21916391921047	-2.74011338069830
C	6.24074505105204	-3.81839691778011	-3.37729602331350
H	6.37200851705898	-3.62596191846787	-8.45957802892769
H	6.29212995881764	-5.40264544451563	-8.67416407848636
H	4.86017628176757	-4.37611110737748	-9.01983603714392
C	5.24220303637138	-4.61957045081065	-6.93904380589582
H	5.88277587552835	-6.50108805147243	-6.73559965409884
F	7.84884624056418	-3.87669524939027	-1.62280593153688
F	8.54526760421149	-3.33140518964364	-3.60339567228643
F	7.36757592647181	-1.93106053677055	-2.44159448875926
O	-2.13799894246710	-7.07279127185462	-0.01277148472926
O	-1.74231666306738	-8.84311913096673	-2.71607218355179
O	-3.22813205207071	-8.09555099641558	-5.80383586985071
O	-3.13394211893276	-5.90179372625686	-3.14497786213116
C	0.09673535400450	-7.57274601925460	-0.72223102748053
C	-0.95668033659254	-6.64900742824109	-0.55941594144759
C	-0.84321604508619	-5.31824093753054	-1.01045589175491
C	-0.08595094658921	-9.04534633999419	-0.38329448720850
H	-1.07487128530799	-9.18561779237761	0.07158701440052
H	0.66020687022525	-9.34718078582931	0.36720778962361
C	0.08591014403507	-9.93010215950308	-1.60718898034151
C	-0.40363103752245	-10.36395608348909	-3.97715221553465
C	-0.70998928593340	-9.73325317262802	-2.75461508523662
C	-1.11558773010100	-9.94872344562335	-5.24893386588257
H	-0.74411949241871	-10.56816201239394	-6.08040295335967
H	-2.19511313429717	-10.12198344290593	-5.17183239146943
C	-0.87633143671954	-8.47762882844814	-5.54913245356084
C	-1.74660142988958	-6.20388717246047	-5.88545171116796
C	-1.95399283073288	-7.59299953812830	-5.76650872710249
C	-2.90302880327690	-5.21246036052186	-5.89780062226215
H	-3.84849986623537	-5.75522488205383	-5.78749779486103
H	-2.94067616351238	-4.68156769673204	-6.86148182090564
C	-2.70105154060999	-4.20349338479143	-4.78259982839290
C	-2.25258767997433	-3.80813491157966	-2.40024472786111
C	-2.73513849943297	-4.62821201300245	-3.43805435195575
C	-2.04121807448879	-4.37779072512223	-1.01252414450118
H	-1.87285172600308	-3.55076424088497	-0.30522080067378
H	-2.92658483955291	-4.92950129466475	-0.67859745673798
C	-2.27883468496323	-6.86945455485728	1.39669377606919
H	-1.46629922636780	-7.40016557639491	1.92762078290809
H	-2.17901052070856	-5.79283096707964	1.62969459790900
C	-3.63682259580725	-7.39319131708100	1.82602635217756
H	-3.71204068610497	-8.45361179171985	1.52945639934790

H	-4.41673922751044	-6.85284400474214	1.26282798033739
C	-3.86698197517683	-7.24268830242769	3.32898533587564
H	-3.81433140164435	-6.18539300260909	3.63827240869811
H	-4.85747248068082	-7.62734523179635	3.61751583654154
H	-3.10964486506193	-7.79766620575390	3.90751922562180
C	-2.93794907251455	-9.25924329257773	-2.03747500573478
H	-3.40313939373011	-8.33551940455588	-1.67583075563514
H	-2.67763404829686	-9.86408977456713	-1.15285805170759
C	-3.88377804530315	-10.02793563665157	-2.94518010563671
H	-4.06124363025126	-9.42199661231306	-3.84649152312465
H	-3.39794806074305	-10.96103903737828	-3.27928080822809
C	-5.20335992496736	-10.34753884718283	-2.24450928449548
H	-5.71425094499276	-9.42493248319684	-1.92243873087828
H	-5.88699023416623	-10.89306884324393	-2.91387984447437
H	-5.04407812934111	-10.96974540344176	-1.34776394214400
C	-3.77700211535394	-8.24683693624080	-7.12338818826994
H	-3.85141626962290	-7.25523076545491	-7.60365195098145
H	-3.08723050073727	-8.86256007808299	-7.72997528489328
C	-5.14368874184624	-8.89959018075227	-7.03122897211392
H	-5.60793652296060	-8.82114590622772	-8.02943164206316
H	-5.77167220104577	-8.30086050767530	-6.34823019384224
C	-5.11234242931902	-10.36208243944037	-6.58809528924798
H	-4.49309328593831	-10.96644538540057	-7.27194035376186
H	-4.69353417669621	-10.46741369892412	-5.57734869968430
H	-6.12531649016922	-10.79473418215509	-6.57850830382547
C	-4.53899464618136	-6.18316846707318	-3.23483233487336
H	-4.99842247584964	-5.55244853222387	-4.01421179798918
H	-4.61839133926073	-7.22851305002576	-3.56428134382591
C	-5.25513782985269	-5.97256699031046	-1.90827718420180
H	-4.75285038317190	-6.57535758112383	-1.13452423202740
H	-5.15024465982413	-4.91665449024731	-1.60535608210118
C	-6.73362998197810	-6.34808032141662	-1.99825078516323
H	-6.85765112236069	-7.40558506663794	-2.28565146757917
H	-7.24131778141600	-6.20155449488468	-1.03211330801051
H	-7.25875542926953	-5.73553578372578	-2.75024973614504
C	5.54907522230437	-7.81566159136038	-4.23434844479295
C	5.31791533293714	-8.73327802962233	-5.35800641100025
H	4.26008239734274	-10.57820938268772	-1.35487245255673
O	4.40039073701153	-9.64535845084997	-4.99316764137552
C	3.84978381887197	-10.51195225474932	-6.01441687543033
C	4.71938716274367	-11.73691755145935	-6.21897845553834
H	2.85587736940690	-10.77793726694858	-5.63393911937445
H	3.73768095307996	-9.93173412451563	-6.94145371421214
H	4.85001694844970	-12.28163267972283	-5.27158428486603
H	4.23631784776211	-12.41369724264647	-6.94195539764139
H	5.70745361570216	-11.45607822031252	-6.61258208928961
Sn	5.97317208133538	-8.65867147027936	-2.23513492148662
C	7.39952687159872	-10.27212666200588	-2.51067209881335
C	6.66653392767066	-7.06124491708609	-0.96251120145864
C	4.10782966193473	-9.50881240450111	-1.55405914983358
H	7.34387977572121	-10.94359732839411	-1.64036045958325
H	7.12711381155513	-10.83664875885097	-3.41533924353617

H	8.42525240101250	-9.89163612034915	-2.61216805801208
H	5.86382300970198	-6.31476969796737	-0.89476002397427
H	7.56367605800533	-6.58603786378634	-1.37917365749657
H	6.88770089236147	-7.46639603677255	0.03604377283262
H	3.35030365471220	-9.39558285003384	-2.33685576176359
H	3.78021665106890	-8.99748951081694	-0.63930384043255
O	5.80710158736993	-8.66950379771934	-6.47415027952886
H	9.49747664393376	-5.93742870742636	-12.65476883582506
H	9.95131639484957	-7.26923524626899	-11.54003945506602
C	8.59551864136909	-6.28008931232748	-6.67856343668300
C	8.96165813333071	-4.99000273328091	-7.12966152083931
H	8.22222505516139	-6.83100149385456	-11.76133610051300
H	8.63524624391050	-7.88352096970893	-9.70182849581749
C	9.22456358155297	-4.74008317127576	-8.46703363880150
C	9.11046821605468	-5.77655893723831	-9.41403307642486
C	8.73560324352961	-7.06656975079673	-8.98771929482846
C	8.49103208574559	-7.30743646215558	-7.63837873315366
O	9.36992288911350	-5.44622157407646	-10.69498489405200
C	9.25019852294329	-6.43432412439858	-11.70802216031917
H	8.18695343806274	-8.30746163434398	-7.32786255164873
H	9.03383298229163	-4.17649189143939	-6.40314877949439
H	9.50676666830249	-3.74419744700977	-8.81461958996800
C	8.37307875974828	-6.47921366931646	-5.25801269306696
C	8.11863268431274	-7.63593795265768	-4.59260480761604
H	8.17141208152999	-7.61502037946920	-3.50624293205599
H	8.10266683868447	-8.61078173761590	-5.08047167857490
H	8.44708997393028	-5.57053943314026	-4.65755388889223

transition state leading to the 1*S*,2*S* product

Br	3.37940183030981	-12.61095965072515	-3.09924177017729
C	1.65226750992923	-10.74220072773290	-1.77045653633465
H	2.29982019258901	-10.90390653374037	-0.90821805247011
C	1.90619003852912	-11.39224979229956	-2.97820655946227
C	1.14487026773552	-11.13106519275308	-4.11754790976976
H	1.38220203994484	-11.61411749555629	-5.06677329642655
C	-2.12621810136615	-2.83010634161645	-4.93295838573865
H	-2.12828437667288	-2.46713486599245	-5.96242087606819
C	-1.72817206866822	-1.99481860576457	-3.88813427360195
C	-1.63221335133690	-2.46491753501313	-2.57843264782857
H	-1.26490953387054	-1.81392077242032	-1.78300556452771
Br	-1.23075473987023	-0.18576774233157	-4.27307047835504
Rh	4.97715358073169	-5.74754562245900	-4.54686459981216
O	2.89882526576583	-4.00687795201581	-2.94764597407250
O	3.93552951743553	-5.99105748955436	-2.70093859232519
O	2.34490536283015	-4.49652049634804	-5.61883766855895
O	3.27386236687702	-6.53720207643290	-5.36589748371401
C	2.97143011184290	-5.18825259157723	-2.48465962085595
C	1.78858077980107	-5.67647432360603	-1.71669338620628
C	1.68433138997073	-7.02018560355921	-1.33356946571768
H	2.51197561057324	-7.69535813763990	-1.54003057243720
C	0.68879056789179	-4.82601875867206	-1.53926917847069

H	0.75273351915589	-3.80195013048697	-1.90686229082675
C	0.76851865361884	-7.77436117055639	-5.56899738933057
H	1.64323024959797	-8.40558268377326	-5.40602015310122
C	0.94065427800740	-6.39147188532755	-5.72348876923558
C	2.28087484045168	-5.76095709736192	-5.58261354879530
C	-0.18514758597521	-5.56689397378263	-5.84100173374897
H	-0.04321857952223	-4.48703327035301	-5.88161938617962
Rh	3.88880432477780	-3.51865281533456	-4.68191507958066
N	5.65597491783389	-5.31222890852011	-6.38888458938583
O	4.85379445365829	-3.17712623191942	-6.45522630748406
O	6.53833273187954	-4.73936801958521	-3.61915836777101
O	5.50710665380175	-2.72204951812490	-3.66519658803729
C	5.97748875201941	-3.95575022481563	-8.39791232953143
C	7.67278552460308	-2.89676845272542	-2.68527369881124
C	6.43853379800294	-3.50645601418491	-3.39032316045695
H	6.61260355680073	-3.05688337268422	-8.41708158192262
H	6.55155509642009	-4.81800899659117	-8.76188253217942
H	5.12296547991037	-3.77299272148627	-9.06815978809135
C	5.46532560099623	-4.16257103597237	-6.99499184042083
H	6.03622062987381	-6.07308273568586	-6.94983659586694
F	7.97443893281229	-3.58566854061752	-1.57507883468706
F	8.73987355861693	-2.95692686357842	-3.50500436712186
F	7.48079956342511	-1.62464649790993	-2.34955183701751
O	-1.71225226101007	-7.09555683598253	0.03329179337828
O	-1.31739332959800	-8.81609047653875	-2.72182934547178
O	-2.87919345878969	-8.04639847390066	-5.75681338072381
O	-2.79183758031590	-5.90471544943442	-3.04902235018610
C	0.51819570726739	-7.51241410504809	-0.74198406587609
C	-0.55835777085112	-6.62537932449273	-0.53399047487208
C	-0.49644397978908	-5.28390105922280	-0.96258641023974
C	0.39218754387151	-8.99587762451910	-0.42850751456095
H	-0.58584333064881	-9.18149463598062	0.03304008064290
H	1.15726127823533	-9.28626245018811	0.30733131733737
C	0.58287281394555	-9.84239583574671	-1.67562457104322
C	0.06698341514592	-10.23956694570605	-4.04584974985266
C	-0.24444866002938	-9.65459604228744	-2.80251501884033
C	-0.68619005905976	-9.82697108184681	-5.29440352934554
H	-0.30951126899083	-10.41616410115094	-6.14522172477719
H	-1.75702757352671	-10.04075289439414	-5.19880585845152
C	-0.50842591008907	-8.34221447013217	-5.57181613075174
C	-1.47488899812322	-6.09751185347437	-5.83658536889573
C	-1.62477303195913	-7.49601651569281	-5.74120150485536
C	-2.66949018066136	-5.15327000091157	-5.79306549358757
H	-3.58837544495813	-5.73652621153002	-5.66554101353397
H	-2.75774131791568	-4.60535411167170	-6.74380644174015
C	-2.47559669280905	-4.15902298425267	-4.66335965359544
C	-1.98489448846262	-3.78956718551455	-2.28497814407706
C	-2.45744275244164	-4.60987869010516	-3.32718237409967
C	-1.71864132089324	-4.37675680608214	-0.91395396203231
H	-1.55177507634620	-3.55711887274723	-0.19774136761991
H	-2.57907976568764	-4.95738597335946	-0.56395288780502
C	-1.81825930032929	-6.93494928450453	1.45121964386293

H	-0.97130057094578	-7.44839170428964	1.94392339504939
H	-1.75124999124305	-5.86169640010833	1.71036044870066
C	-3.14252117883754	-7.52060056982507	1.90512475454413
H	-3.18614153942838	-8.57555307565957	1.58379559961246
H	-3.95833358143281	-6.99699744006630	1.37821842587162
C	-3.33403418917976	-7.41541498697045	3.41731641540251
H	-3.31256393895686	-6.36459414460326	3.75139862960521
H	-4.30030580100377	-7.84481385429425	3.72426597102146
H	-2.53948315703451	-7.95442038721911	3.95990298483810
C	-2.47160473909401	-9.30412776288273	-2.01987406692957
H	-2.96840005284967	-8.41343482680428	-1.61894850093355
H	-2.15674997634640	-9.91978582613854	-1.16092454170741
C	-3.40721863849597	-10.09357201139052	-2.92001034326748
H	-3.65128882235921	-9.47043469145252	-3.79348814966743
H	-2.88329879013334	-10.98634805229627	-3.30297748052461
C	-4.68003130302275	-10.50984905823460	-2.18478676970992
H	-5.22212459511949	-9.62940407975119	-1.80130518934171
H	-5.36262648933637	-11.05975605913701	-2.85158637157002
H	-4.45426266248270	-11.16129144328507	-1.32379793619671
C	-3.45792321006690	-8.18679275311504	-7.06476771689610
H	-3.58114926034982	-7.18773727978337	-7.51875572643818
H	-2.76324996390142	-8.76247290975701	-7.70420117636774
C	-4.79705532386861	-8.89132657551587	-6.95234244465491
H	-5.29167506586650	-8.80566489145456	-7.93522319656932
H	-5.42656799847422	-8.33274324099026	-6.23750759674868
C	-4.70002980899485	-10.36199844976556	-6.54741158635622
H	-4.08117374806467	-10.92670678901067	-7.26468107434899
H	-4.24635665557624	-10.47608789792549	-5.55282393572519
H	-5.69616598065027	-10.83130903763516	-6.51813693628739
C	-4.18439517663520	-6.24917550295724	-3.10810153600378
H	-4.69549776906353	-5.61933716721675	-3.85542560985444
H	-4.22469989568678	-7.28733327138592	-3.46610986616195
C	-4.86939690763601	-6.10975118116638	-1.75601664474544
H	-4.31088139950673	-6.70241286925643	-1.01378838744907
H	-4.81140152073939	-5.05727583639319	-1.42937838170711
C	-6.32806849657538	-6.56139991852045	-1.81176076030392
H	-6.40562625831848	-7.61702308213990	-2.12146033154239
H	-6.81307068827454	-6.46440107210680	-0.82785400863332
H	-6.90758887563628	-5.96043944923036	-2.53255169291781
C	5.89287538417817	-7.49938720036555	-4.47374386915184
C	5.57758472662848	-8.37877391176424	-5.61614216010710
H	4.77802888754427	-10.25150565173163	-1.44034008999202
O	4.74978973604228	-9.35292781285963	-5.20309073840811
C	4.17256124182338	-10.22697639588138	-6.20308677504853
C	5.10053782115096	-11.38499160605197	-6.51363865077127
H	3.23397948301228	-10.56786336830014	-5.75151001456147
H	3.94593304827458	-9.63217783578126	-7.09916540947868
H	5.34232609437532	-11.94364450752930	-5.59684445114115
H	4.60579168401237	-12.07347641516208	-7.21734445652019
H	6.03342519669296	-11.02835191205590	-6.97482883805421
Sn	6.50079333535284	-8.49585032809103	-2.60077175522138
C	7.63621055551300	-10.25919756506636	-3.17132025743420

C	7.43379096789574	-7.09962045590145	-1.24526922534382
C	4.63932490202448	-9.20702416732864	-1.75286284242581
H	8.50635551936320	-10.39010361065126	-2.51304592829789
H	6.96632089871308	-11.12655183431572	-3.07607606897277
H	7.97617157970733	-10.18246318702707	-4.21349730576708
H	7.44118993005035	-6.10374009085026	-1.70269818249314
H	8.46220208129869	-7.40961349847142	-1.01952751986592
H	6.83755674137320	-7.07937251160810	-0.32112569985044
H	3.85010650113612	-9.16355888634952	-2.51145265481223
H	4.36337991816766	-8.58913912817991	-0.88866216787579
O	5.94063878778561	-8.24396697290918	-6.77305259338047
C	8.31114811965027	-7.14039660587663	-5.32415562945007
C	8.99767216539585	-6.39321731151861	-4.41924746473769
C	9.95681167962261	-6.85373641157242	-3.43122393768223
C	10.47620776257502	-5.93516101364730	-2.49718378962935
C	11.35994275299251	-6.32587606757735	-1.49361264412072
C	11.75443974747189	-7.67292330104715	-1.40446691389565
C	11.26444470295977	-8.60302153052041	-2.34727244543771
C	10.39022695708065	-8.19778571977088	-3.34093646908357
O	12.59427570517509	-8.15999012057650	-0.47061389894768
C	13.12597408707015	-7.28254402472650	0.51213842439224
H	7.73392970746101	-6.63813809657480	-6.09369405940515
H	8.46947238083450	-8.21341173177901	-5.44482655471569
H	8.80199985976141	-5.31932073521358	-4.40866353640419
H	10.16220095649436	-4.89124367038127	-2.54928525168227
H	11.72903504414451	-5.58425196907674	-0.78592066434238
H	11.59500350816308	-9.64118850716766	-2.27332544186397
H	10.03494549154598	-8.93346369215016	-4.06278239018133
H	12.32442374961836	-6.82750976605289	1.11812036363028
H	13.72874203800562	-6.48238492694676	0.05063171212145
H	13.76779516612666	-7.89485362823617	1.15824674546078

Experimental Section

General

Unless stated otherwise, all reactions were carried out under argon atmosphere in flame-dried Schlenk glassware, ensuring inert conditions. The solvents were purified by distillation over the indicated drying agents and were transferred under argon: THF, Et_2O (Mg/anthracene); pentane, toluene (Na/K); CH_2Cl_2 (CaH_2). MeCN and 1,4-dioxane were dried by an absorption solvent purification system based on molecular sieves. tert-Butyl acetate and chlorobenzene were bought from TCI and Sigma-Aldrich, respectively, and used as received.

Flash chromatography: Merck Geduran silica gel 60 (40 – 63 µm). Thin layer chromatography (TLC): Macherey-Nagel precoated plates (POLYGRAM®SIL/UV254); visualization by UV light (254 nm) and by staining with solutions of phosphomolybdic acid (PMA), $KMnO_4$ or cerium ammonium nitrate (CAN).

NMR spectra were recorded on a Bruker Avance III HD nanobay 300, Avance III HD 400, Avance III 500 or Avance Neo 600 MHz NMR spectrometer. ^{1}H and ^{13}C NMR chemical shifts are given in ppm relative to Me_4Si (δ = 0 ppm), coupling constants (J) in Hz. ^{1}H and ^{13}C NMR chemical shifts were referenced using the solvent signals internal reference;[22] For ^{1}H NMR the following residual proton peaks of the deuterated solvents were used: $CDCl_3$: δ_H($CHCl_3$) 7.26 ppm; CD_2Cl_2: δ_H ($CHDCl_2$) 5.32 ppm; [D_8]-THF: δ_H(($CD_2)_3CHDO$) 3.58 ppm; CD_3CN: δ_H(CHD_2CN) 1.94 ppm; for ^{13}C NMR: $CDCl_3$: δ 77.16 ppm; CD_2Cl_2: δ 54.0 ppm; [D_8]-THF: δ 67.57 ppm; CD_3CN: δ 1.32 ppm. ^{19}F NMR chemical shifts are reported relative to CCl_3F. ^{119}Sn NMR spectra were recorded using Me_4Sn as an external standard. Unless stated otherwise, all ^{13}C, ^{19}F and ^{119}Sn NMR spectra were recorded in proton-decoupled manner. IR: Alpha Platinum ATR (Bruker), wavenumbers ($\tilde{\nu}$) in cm^{-1}.

MS (EI): Finnigan MAT 8200 (70 eV), ESI-MS: ESQ 3000 (Bruker) or Thermo Scientific LTQ-FT or Thermo Scientific Exactive Spectrometer. HRMS: Bruker APEX III FT-MS (7 T magnet), MAT 95 (Finnigan), Thermo Scientific LTQ-FT or Thermo Scientific Exactive Spectrometer.

Optical rotations were measured with an A-Krüss Otronic Model P8000-t polarimeter at a wavelength of 589 nm. The values are given as specific optical rotation with exact temperature, concentration (c in g/100 mL) and solvent.

HPLC: analytical LC analyses were conducted on a Shimadzu LC 2020 instrument equipped with a Shimadzu SPD-M20A UV/VIS detector or on an Agilent system equipped with a G4212A 1290 DAD. Preparative separations were performed on a Shimadzu Prominence prep HPLC system equipped with two LC-20 AP preparative pumps, a SIL-20 AC HT sample injector, CTO-20AC oven, SPD-20A UV-

detector with variable flow cell, a FRC-10 A fraction collector and a CBM 20A controller module. The specific column employed and respective solvent mixture are indicated for each experiment.

UV-VIS measurements have been performed from 1200 nm to 200 nm at room temperature on an Agilent Cary6000i double beam spectrometer. The circular dichroism spectra were measured on a J-1100 CD UV-Visible/NIR spectrometer. A high precision cell made of quartz (Suprasil Hellma Analytics cuvettes) were used for the measurements.

[Rh_2(acam)(Otfa)$_3$] (**5**),[23] calix[4]arene dicarboxylic acid **6**,[24] the α-stannylated α-diazoester derivative **2**,[25] the donor/acceptor diazoester derivative **9**,[26] and ethyl 3,3,3-trifluoro-2-diazopropionate (**11**)[27] were prepared according to literature procedures.

Unless stated otherwise, all commercially available compounds (Alfa Aesar, Sigma Aldrich, TCI, BLD Pharm, ABCR and SCBT) were used as received.

Catalyst Screening and Reaction Optimization

Table S1. Preliminary Screening and Reaction Optimization

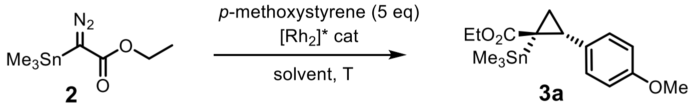

Entry	T [°C]	solvent	addition time diazo	[Rh_2]* cat	mol% cat	Yield % [^{1}H NMR]^a	*cis:trans*	ee %
1	rt	CH_2Cl_2	5 h	*P*-**7**	0.5	15 [37]	10:1	98
2	rt	CH_2Cl_2	5h	*P*-**7**	1.0	[30]	10:1	98
3	rt	CH_2Cl_2	5 h	*rac*-**S8**	0.5	[22]	7:1	n.d.
4	rt	pentane	<1 min	*P*-**7**	0.5	[65]	9:1	n.d.
5	-20	CH_2Cl_2	<1 min	*P*-**7**	0.5	[15]	10:1	n.d.
6	rt	CH_2Cl_2	<1 min	*P*-**S7**	0.5	[61]	12:1	89
7[b]	rt	CH_2Cl_2	<1 min	*P*-**7**	0.5	40 [75]	10:1	n.d.
8	rt	CH_2Cl_2	<1 min	*P*-**8**[d]	0.5	37 [64]	3:1	48
9[c]	rt	CH_2Cl_2	<1 min	*P*-**7**	0.5	58 [85]	10:1	98

[a] For the screening, the reactions were performed on a 0.1 mmol scale. Yields in brackets were determined by ^{1}H NMR analysis of the crude material using mesitylene as internal standard. All other yields are isolated yields. [b] Molecular sieves (4Å) were added to the reaction. [c] The reaction was performed using freshly sublimed diazo ester **2** (<48 h prior to use). [d] The chosen catalyst was tentatively assigned as *P*-**8**

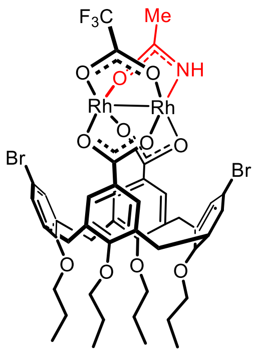

P-**7**

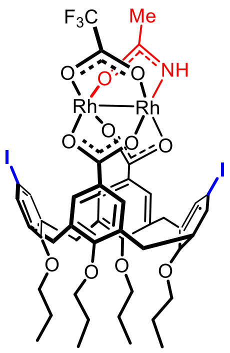

P-**S7**

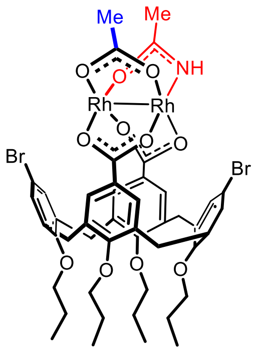

rac-**S8**

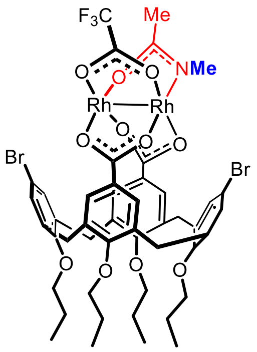

P-**8**

The results of the preliminary screening showed that

- very rapid addition of the diazoester **2** to the mixture of the olefin and the catalyst is mandatory for a clean conversion and hence good yields of the cyclopropane product **3a**
- replacement of the trifluoroacetate ligand in *P*-**7** by an ordinary acetate in *rac*-**S8** resulted in a lower dr (entry 3); as the preparation of *rac*-**S8** required extra steps, this catalyst was not investigated any further at this point
- formal replacement of the bromo substituents in *P*-**7** by iodo substituents (*P*-**S7**) furnished cyclopropane **3a** with slightly better dr but a lower ee (entry 6); therefore more systematic studies on how different substituents at the alternating arene rings of the calixarene scaffold affect the outcome of the reaction were postponed to a later stage of the project
- N-methylation of the acetamidate ligand as manifested in complex *P*-**8** is detrimental (entry 8); the protic site in *P*-**7** is hence critically important for success.

Ligand Synthesis

Compound S1. DMAP (17.5 mg, 0.14 mmol) and MeOH (87 µL, 2.15 mmol) were added to a solution of the calix[4]arene dicarboxylic acid derivative **6** (600 mg, 0.72 mmol) in DMF/CH_2Cl_2 (1:2 *v/v*, 9 mL). The mixture was cooled to 0°C and stirred for 15 min prior to the portionwise addition of EDC (412 mg, 2.15 mmol). After stirring for 4 h, the mixture was diluted with water and extracted with CH_2Cl_2 (3 x 40 mL). The organic phase was washed with brine (2 x 40 mL), dried over $MgSO_4$, filtered and concentrated under reduced pressure. The residue was purified by flash chromatography (silica, hexane/EtOAc 10:1 to 7:1) to give the title compound as a white solid (542 mg, 87%). ^{1}H NMR (400 MHz, $CDCl_3$) δ 7.70 (s, 4H), 6.45 (s, 4H), 4.40 (d, *J* = 13.5 Hz, 4H), 4.08 – 4.00 (m, 4H), 4.02 (s, 6H), 3.69 (t, *J* = 7.0 Hz, 4H), 3.20 (d, *J* = 13.5 Hz, 4H), 1.98 – 1.80 (m, 8H), 1.06 (t, *J* = 7.4 Hz, 6H), 0.90 (t, *J* = 7.5 Hz, 6H). ^{13}C NMR (101 MHz, $CDCl_3$) δ 167.1, 161.7, 154.8, 136.0, 135.4, 130.8, 130.7,

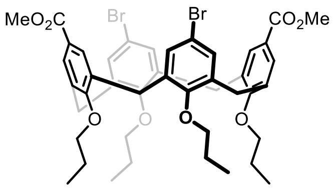

124.4, 115.5, 77.5 (overlap $CDCl_3$), 77.0, 52.1, 31.0, 23.5, 23.2, 10.7, 10.0. IR (ATR): ṽ = 2961, 2934, 2876, 1720, 1456, 1433, 1316, 1294, 1199, 1003, 963 cm^{-1}. HRMS (ESI +): *m/z calcd.* for $C_{44}H_{50}O_8Na$ $[M+Na]^+$: 887.17646; *found:* 887.17588.

Compound S2. A flame dried pressure Schlenk flask was charged with CuI (7 mg, 0.035 mmol, 10 mol %), calix[4]arene derivative **S1** (300 mg, 0.35 mmol), NaI (208 mg, 1.4 mmol), racemic *trans*-N,N'-dimethyl-1,2-cyclohexanediamine (11 µL, 0.07 mmol, 20 mol%) and 1,4-dioxane (1.5 mL). The resulting mixture was stirred at 110°C (bath temperature) for 22 h. The suspension was cooled to room temperature and diluted with a 30% aqueous ammonia solution (5 mL). The mixture was poured into water (20 mL), and extracted with dichloromethane (3 × 15 mL). The combined organic phases were dried over $MgSO_4$, filtered and concentrated under reduced pressure. The residue was purified by flash chromatography (silica, cyclohexane/EtOAc 14:1) to give the title compound as a white solid (304 mg, 91 %). ^1H NMR (400 MHz, $CDCl_3$) δ 7.61 (s, 4H), 6.75 (s, 4H), 4.37 (d, J = 13.4 Hz, 4H), 4.07 – 3.97 (m, 4H), 3.91 (s, 6H), 3.72 (t, J = 7.1 Hz, 4H), 3.18 (d, J = 13.5 Hz, 4H), 1.98 – 1.80 (m, 8H), 1.04 (t, J = 7.4 Hz, 6H), 0.92 (t, J = 7.5 Hz, 6H). ^{13}C NMR (101 MHz, $CDCl_3$) δ 167.0, 161.4, 155.9, 137.0, 136.1, 135.6, 130.5, 124.4, 86.3, 77.5 (overlap $CDCl_3$), 77.0, 52.1, 30.8, 23.4, 23.2, 10.6, 10.1. IR (ATR): ṽ = 2961, 2933, 2875, 1719, 1456, 1433, 1316, 1290, 1231, 1198, 1163, 1003, 962, 769 cm^{-1}. HRMS (ESI +): *m/z calcd.* for $C_{44}H_{50}O_8I_2Na$ $[M+Na]^+$: 983.14874; *found:* 983.15002.

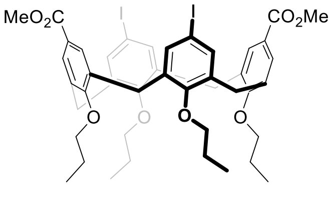

Compound S3. KOTMS (267 mg, 2.1 mmol) was added to a solution of calix[4]arene derivative **S2** (200 mg, 0.21 mmol) in THF (7mL) and the resulting mixture stirred at room temperature until full consumption of the starting material was detected by TLC (2-3 d). An aq. solution of citric acid (3 M, 2 mL) was carefully added and stirring continued for 1 h at ambient temperature. The mixture was diluted with water and extracted with CH_2Cl_2 (3 x 20 mL). The combined organic phases were washed with brine (50 mL), dried over $MgSO_4$, filtered and concentrated under reduced pressure. The crude product was washed with cold *n*-pentane (2 x 3mL) to afford the title compound as a white solid (158 mg, 83 %). ^1H NMR (400 MHz, $CDCl_3$) δ 12.82 (br s, 2H), 7.50 (s, 4H), 6.83 (s, 4H), 4.35 (d, J = 13.8 Hz, 4H), 4.01 – 3.92 (m, 4H), 3.64 (t, J = 6.7 Hz, 4H), 3.10 (d, J = 13.9 Hz, 4H), 1.93 – 1.75 (m, 8H), 1.08 (t, J = 7.4 Hz, 6H), 0.83 (t, J = 7.5 Hz, 6H). ^{13}C NMR (101 MHz, $CDCl_3$) δ 171.8, 159.9, 157.9, 139.1, 138.0, 133.3, 129.9, 123.8, 86.2, 77.4, 76.7, 30.8, 23.6, 22.8, 10.9, 9.8. IR (ATR): ṽ = 2963, 2932, 2876, 1700, 1461, 1423, 1385, 1305, 1284, 1202, 1110, 1001, 964 cm^{-1}. HRMS (ESI -): *m/z calcd.* for $C_{42}H_{45}O_8I_2$ $[M-H]^-$: 931.12094; *found:* 931.12153.

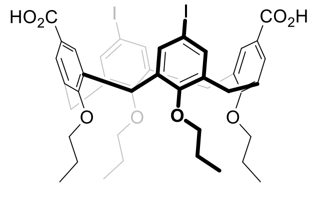

New Dirhodium Complexes

Rh$_2$(*N*-Me-acam)$_4$ (S4). A 50 mL two-neck flask was equipped with a returning-arm frit (or Soxhlet extractor) topped by a reflux condenser. The Soxhlet extractor was filled with an oven-dried mixture of K_2CO_3 and sand (1:1, ca. 3 g). The flask was charged with [$Rh_2(OAc)_4\cdot 2H_2O$] (250 mg, 1.05 mmol), *N*-methylacetamide (2.30 g, 31.4 mmol) and chlorobenzene (40 mL). Argon was bubbled through the reaction mixture for 15 min, before the flask was immersed into a pre-heated oil bath (165 °C bath temp). The mixture was stirred at reflux temperature such that a gentle flow of condensing solvent passed through the Soxhlet extractor. After stirring for 48 h, the mixture was cooled to room temperature and chlorobenzene was removed by distillation under reduced pressure (30-40°C, $1\cdot 10^{-3}$ mbar). The residual brown solid was purified by sublimation under reduced pressure (65-70°C, 1·10-3 mbar) to remove any remaining *N*-methylacetamide; this purification afforded the title complex as blue solid, which is essentially insoluble in deuterated DMSO, acetonitrile, water and chloroform; therefore, no NMR spectra could be recorded. IR (ATR): $\tilde{\nu}$ = 2920, 1433, 1373, 1350, 1112, 1021, 849, 658, 636, 618, 595 cm^{-1}. HRMS (ESI +): *m/z calcd.* for $C_{12}H_{24}O_4N_4Rh_2Na$ [M+Na]$^+$: 516.97998; *found:* 516.98001.

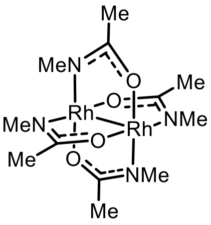

Rh$_2$(*N*-Me-acam)(Otfa)$_3$ (S5). Rh$_2$(*N*-Me-acam)$_4$ (**S4**) (159 mg, 0.32 mmol) was filled into a flame dried two neck round bottom flask connected to a reflux condenser. Trifluoroacetic acid (6.2 mL, 80.4 mmol) was added and the flask was immersed into a preheated oil bath at 60°C. The mixture was stirred for 42 min and then quickly cooled below ambient temperature in an ice bath. All volatile materials were distilled off under reduced pressure. The remaining crude material was purified by flash chromatography (silica, toluene/MeCN 99:1 to 1:1) to give the title compound as a purple solid (88 mg, 44%). ^{1}H NMR (400 MHz, [D$_8$]-THF) δ 3.02 (s, 3H), 1.96 (s, 3H). ^{13}C NMR (101 MHz, [D$_8$]-THF) δ 186.4, 174.7, 38.6, 20.7. ^{19}F NMR (282 MHz, [D$_8$]-THF) δ –76.06 (6F), –76.17 (3F). IR (solid, ATR) $\tilde{\nu}$ = 1660, 1392, 1192, 1156, 858, 784, 738, 530 cm^{-1}. HRMS (ESI +): *m/z calcd.* for $C_9H_6F_9NO_7Rh_2$ [M+H]$^+$: 617.81834; *found:* 617.81823.

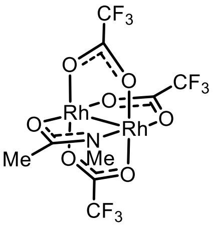

Complex 7. A two-neck flask was equipped with a Soxhlet extractor (returning-arm frit) topped by a reflux condenser. The Soxhlet extractor was filled with an oven-dried mixture of K_2CO_3 and sand (1:1, ca. 5 g). The flask was charged with the calix[4]arene derivative **6** (334 mg, 0.40 mmol) and Rh$_2$(acam)(Otfa)$_3$ (**5**) (200 mg, 0.33 mmol), followed by addition of *tert*-butyl acetate (40 mL) and toluene (12 mL). Argon was bubbled through the mixture for 15 min, before the flask was immersed into a pre-heated oil bath (130 °C bath

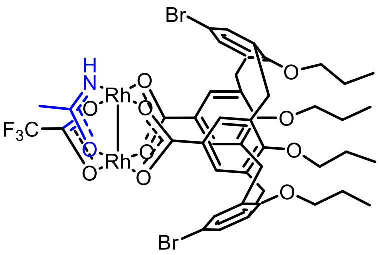

temperature). The mixture was stirred at reflux temperature such that a gentle flow of condensing solvent passed through the Soxhlet extractor. (*Note*: initially the reaction mixture is a suspension, but as the reaction progresses, the suspension slowly becomes a transparent green solution). After 5 h, the mixture was cooled to room temperature and concentrated under reduced pressure. The green residue was purified by flash chromatography (silica, toluene/acetonitrile 12:1 to 7:1) to afford the title compound as a green solid (147 mg, 37%). ^{1}H NMR (600 MHz, [D$_8$]-THF) δ 7.39 (d, *J* = 2.5 Hz, 1H), 7.37 (d, *J* = 2.7 Hz, 2H), 7.35 (d, *J* = 2.5 Hz, 1H), 6.74 – 6.71 (m, 2H), 6.66 (d, *J* = 2.2 Hz, 1H), 6.59 (d, *J* = 2.2 Hz, 1H), 5.27 (s, 1H), 4.41 – 4.34 (m, 4H), 4.00 – 3.95 (m, 4H), 3.68 – 3.63 (m, 4H), 3.19 – 3.11 (m, 4H), 1.88 – 1.81 (m, 8H), 1.80 (s, 3H), 1.08 (t, *J* = 7.4 Hz, 3H), 1.08 (t, *J* = 7.4 Hz, 3H), 0.82 (t, *J* = 7.5 Hz, 3H), 0.82 (t, *J* = 7.5 Hz, 3H). ^{13}C NMR (151 MHz, [D$_8$]-THF) δ 186.46, 185.71, 184.26, 171.99 (q, *J* = 37.9 Hz), 159.43, 159.28, 158.23, 158.19, 140.34, 140.23, 140.18, 140.09, 132.97, 132.90, 132.88, 132.73, 132.57 (d, *J* = 3.5 Hz), 130.30, 129.97, 129.80, 129.40, 127.92, 127.03, 115.19, 115.05, 111.87 (q, *J* = 286.3 Hz), 77.76, 77.46, 77.38, 31.58 (t, *J* = 5.6 Hz), 24.33, 23.68, 23.62, 23.35, 11.14, 11.12, 9.95, 9.93. ^{19}F NMR (565 MHz, THF) δ -75.83. IR (ATR): ṽ = 2964, 2934, 2877, 1637, 1570, 1403, 1384, 1200, 1159, 959, 740 cm^{-1}. HRMS (ESI +): *m/z calcd.* for $C_{46}H_{48}Br_2F_3NO_{11}Rh_2Na$ [M+Na]$^+$: 1233.95485; *found:* 1233.95486.

HPLC analysis: 150 mm Chiralcel IB-N3, 4.6 mm Ø, acetonitrile/water = 70:30, v = 1.0 mL / min, λ = 220 nm, t(enantiomer 1) = 12.79 min, t(enantiomer 2) = 14.06 min. The enantiomers were separated by preparative HPLC using the following conditions: 250 mm YMC Chiral Art Cellulose-SB, 5µm, 20.0 mm ∅, acetonitrile / water= 70:30 , *v* = 15.0 mL / min, λ = 220 nm. The overall yield of each enantiomer ranged between 9-13%.

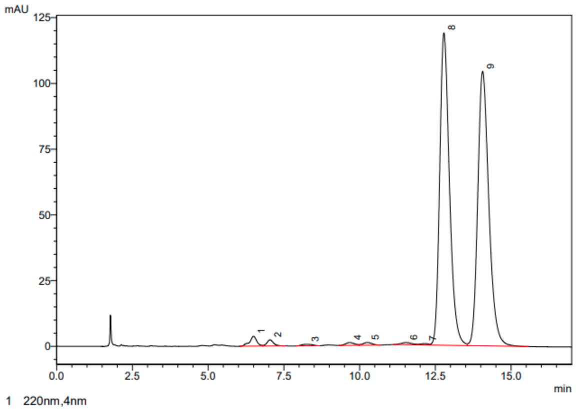

1 220nm,4nm

PDA Ch1 220nm

Peak #	Ret. Time	Area %	Name
1	6.50	1.13	
2	7.04	0.65	
3	8.28	0.26	
4	9.68	0.41	
5	10.26	0.39	
6	11.53	0.38	
7	12.17	0.19	
8	12.79	48.21	1. enantiomer
9	14.06	48.38	2. enantiomer
Total		100.00	

Separation of the enantiomers of complex **7** by HPLC under the conditions described above

CD spectra of complex **7** were measured in 10 mm Quartz cuvettes with a concentration of 1 mg/mL in *tert*-butyl methyl ether, see Figure S8 and Figure 1 (main text).

The UV-VIS spectrum of complex **7** was measured in a 2 mm Suprasil Quartz cuvette at a concentration of 1.8 mM in *tert*-butyl methyl ether (see Figures S5 and S6). A cuvette with pure solvent (*tert*-butyl methyl ether) was placed in the reference beam. The measured absorption was subsequently converted to molar absorption coefficient using the Lambert-Beer law. Two relatively weak bands at 624 nm and 425 nm are present with molar absorption coefficients of 115 $M^{-1}cm^{-1}$ and 120 $M^{-1}cm^{-1}$, respectively.

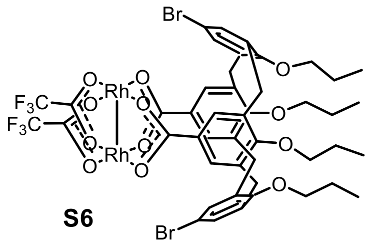

S6

During flash chromatography, a second fraction was isolated. Subsequent trituration of the remaining solid with *tert*-butyl methyl ether afforded complex **S6** as a green solid (70 mg, 16%). ^{1}H NMR (600 MHz, [D$_8$]-THF) δ 7.41 (s, 4H), 6.68 (s, 4H), 4.37 (d, *J* = 13.9 Hz, 4H), 4.01 – 3.95 (m, 4H), 3.68 (t, *J* = 6.7 Hz, 4H), 3.17 (d, *J* = 13.7 Hz, 4H), 1.89 – 1.79 (m, 8H), 1.08 (t, *J* = 7.4 Hz, 6H), 0.82 (t, *J* = 7.5 Hz, 6H). ^{13}C NMR (151 MHz, [D$_8$]-THF) δ 188.21, 174.30, 174.05, 173.79, 173.53, 160.20, 158.09, 140.10, 133.44, 132.75, 130.03, 126.14, 115.26, 114,25, 112.36, 110.47, 108.58, 77.89, 77.47, 31.47, 24.31, 23.69, 11.09, 9.92. ^{19}F NMR (565 MHz, [D$_8$]-THF) δ –75.91. IR (ATR): ṽ = 2963, 2926, 1647, 1462, 1403, 1383, 1196, 1159, 1111, 1001, 958, 866, 740 cm^{-1}. HRMS (ESI +): *m/z calcd.* for $C_{46}H_{44}Br_2F_6O_{12}Rh_2Na$ [M+Na]$^+$: 1288.91060; *found:* 1288.91081.

Complex S7. A two-neck flask was equipped with a Soxhlet extractor (returning-arm frit) topped by a

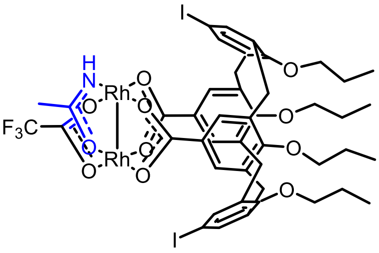

reflux condenser. The Soxhlet extractor was filled with an oven-dried mixture of K_2CO_3 and sand (1:1, ca. 3 g). The flask was charged with the calix[4]arene diacid derivative **S3** (57 mg, 0.06 mmol) and $Rh_2(acam)(Otfa)_3$ (**5**) (30 mg, 0.05 mmol), followed by addition of *tert*-butyl acetate (8 mL) and toluene (2 mL). Argon was bubbled through the mixture for 15 min, before the flask was immersed into a pre-heated oil bath (130 °C bath temperature). The mixture was stirred at reflux temperature such that a gentle flow of condensing solvent passed through the Soxhlet extractor. (*Note*: initially the reaction mixture is a suspension, but as the reaction progresses, the suspension slowly becomes a transparent green solution). After 5 h, the mixture was cooled to room temperature and concentrated under reduced pressure. The green residue was purified by flash chromatography (silica, toluene/acetonitrile 12:1 to 7:1) to afford the title compound as a green solid (35 mg, 54 %). ^{1}H NMR (600 MHz, [D$_8$]-THF) δ 7.57 (d, *J* = 2.3 Hz, 1H), 7.55 (d, *J* = 2.3 Hz, 1H), 7.54 (d, *J* = 2.3 Hz, 1H), 7.53 (d, *J* = 2.3 Hz, 1H), 6.72 (d, *J* = 2.3 Hz, 1H), 6.71 (d, *J* = 2.1 Hz, 1H), 6.65 (d, *J* = 2.1 Hz, 1H), 6.58 (d, *J* = 1.9 Hz, 1H), 5.28 (s, 1H), 4.35 (d, *J* = 13.8 Hz, 2H),

4.34 (d, J = 13.3 Hz, 2H), 3.99 – 3.96 (m, 4H), 3.67 – 3.63 (m, 4H), 3.15 (d, J = 14.0 Hz, 1H), 3.14 (d, J = 14.0 Hz, 1H), 3.13 (d, J = 14.0 Hz, 1H), 3.11 (d, J = 14.0 Hz, 1H), 1.87 – 1.81 (m, 8H), 1.80 (s, 3H), 1.08 (t, J = 7.4 Hz, 3H), 1.07 (t, J = 7.4 Hz, 3H), 0.81 (t, J = 7.5 Hz, 3H), 0.80 (t, J = 7.5 Hz, 3H). ^{13}C NMR (151 MHz, [D$_8$]-THF) δ 186.63, 185.93, 184.50, 172.15 (q, J = 37.7 Hz), 159.60, 159.46, 159.26, 159.22, 140.93, 140.79, 140.76, 140.67, 138.99, 138.87, 138.82, 138.76, 133.20, 133.14, 133.11, 132.96, 130.47, 130.16, 129.95, 129.54, 128.09, 127.21, 112.04 (q, J = 286.0 Hz), 86.37, 86.25, 77.92, 77.64, 77.53, 31.61 (t, J = 6.5 Hz), 24.50, 23.83, 23.76, 23.53, 11.31 (d, J = 3.1 Hz), 10.08 (d, J = 3.5 Hz). ^{19}F NMR (282 MHz, [D$_8$]-THF) δ –75.83. IR (ATR): ṽ = 2965, 2933, 2876, 1639, 1462, 1403, 1384, 1294, 1200, 1158, 1113, 1002, 959, 840, 740 cm^{-1}. HRMS (ESI +): *m/z calcd.* for $C_{46}H_{48}I_2F_3NO_{11}Rh_2Na$ [M+Na]$^+$: 1329.92712; *found:* 1329.92715.

HPLC analysis: 150 mm Chiralcel IB-N3, Ø 4.6 mm i.D., acetonitrile/water = 75:25, v= 1.0 mL / min, λ = 220 nm, t(enantiomer 1) = 8.69 min, t(enantiomer 2) = 9.75 min. The enantiomers were separated by preparative HPLC using following conditions: 250 mm YMC Chiral Art Cellulose-SB, 5µm, 20.0 mm ∅, acetonitrile/water= 70:30 , v = 15.0 mL / min, λ = 220 nm. The overall yield of each enantiomer ranged between 14-16%.

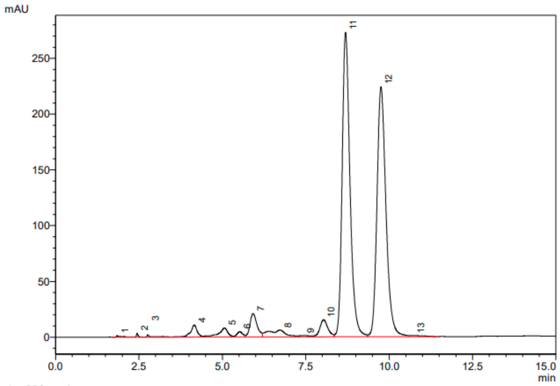

1 220nm,4nm

PDA Ch1 220nm

Peak #	Ret. Time	Area %	Name
1	1.85	0.15	
2	2.44	0.15	
3	2.76	0.25	
4	4.16	1.56	
5	5.06	1.60	
6	5.52	0.59	
7	5.92	3.13	
8	6.73	2.23	
9	7.42	0.32	
10	8.03	2.57	
11	8.69	44.83	1. Enantiomer
12	9.75	42.31	2. Enantiomer
13	10.72	0.30	
Total		100.00	

Separation of the enantiomers of complex **S7** by HPLC under the conditions described above

Complex 8. A two-neck flask was equipped with a Soxhlet extractor (returning-arm frit) topped by a reflux condenser. The Soxhlet extractor was filled with an oven-dried mixture of K_2CO_3 and sand (1:1, ca. 3 g). The flask was charged with the calix[4]arene diacid derivative **6** (49 mg, 0.06 mmol) and Rh_2(*N*-Me-acam)(Otfa)$_3$ **S5** (30 mg, 0.05 mmol), followed by addition of *tert*-butyl acetate (8 mL) and toluene (2 mL). Argon was bubbled through the mixture for 15 min, before the flask was immersed into a pre-heated oil bath (130 °C bath temperature). The mixture was stirred at reflux temperature such that a gentle flow of condensing solvent passed through the Soxhlet extractor (*Note*: initially the reaction mixture is a suspension, but as the reaction progresses, the suspension slowly becomes a transparent green solution). After 4 h, the mixture was cooled to room temperature and concentrated under reduced pressure. The green residue was purified by flash chromatography (silica, toluene/acetonitrile 10:1 to 7:1) to give the title compound as a green solid (29 mg, 48 %). ^{1}H NMR (600 MHz, THF) δ 7.39 – 7.35 (m, 4H), 6.75 (d, *J* = 2.1 Hz, 1H), 6.70 (d, *J* = 2.1 Hz, 1H), 6.65 (d, *J* = 2.1 Hz, 1H), 6.60 (d, *J* = 2.2 Hz, 1H), 4.39 – 4.35 (m, 4H), 4.04 – 3.94 (m, 4H), 3.70 – 3.63 (m, 4H), 3.21 – 3.08 (m, 4H), 3.02 (s, 3H), 1.88 – 1.81 (m, 11H), 1.08 (t, *J* = 7.4 Hz, 3H), 1.08 (t, *J* = 7.4 Hz, 3H), 0.82 (t, *J* = 7.8 Hz, 3H), 0.81 (t, *J* = 7.5 Hz, 3H). ^{13}C NMR (151 MHz, [D$_8$]-THF) δ 185.79, 184.29, 183.07, 171.95 (q, *J* = 38.0 Hz), 159.51, 159.34, 158.23 (d, *J* = 3.0 Hz), 140.32, 140.24, 140.21, 140.07, 133.01, 132.99, 132.95, 132.82, 132.78, 132.75, 132.58, 132.50, 130.45, 130.00, 129.62, 129.35, 128.00, 126.95, 115.20, 114.99, 111.88 (q, *J* = 285.7 Hz), 77.77 (d, *J* = 2.3 Hz), 77.54, 77.32, 38.59, 31.59 (dd, *J* = 16.9, 7.1 Hz), 24.33, 23.70, 23.55, 19.97, 11.13 (d, *J* = 2.9 Hz), 9.95, 9.89. ^{19}F NMR (565 MHz, [D$_8$]-THF) δ –75.86. IR (ATR): ṽ = 2962, 2930, 2876, 1633, 1590, 1519, 1462, 1404, 1383, 1200, 1159, 1113, 1003, 960, 864 cm^{-1}. HRMS (ESI +): *m/z calcd.* for $C_{47}H_{50}Br_2F_3NO_{11}Rh_2Na$ [M+Na]$^+$: 1247.9705; *found:* 1247.97017.

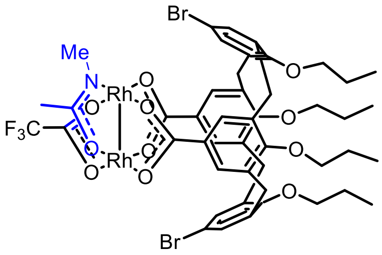

HPLC analysis: 150 mm Chiralpak IE-3, 4.6 mm ∅, acetonitrile/water = 82:18, v = 1.0 mL / min, λ = 220 nm, t(enantiomer 1) = 6.54 min, t(enantiomer 2) = 9.69 min. The enantiomers were separated by preparative HPLC using following conditions: 250 mm Chiralpak IE, 5µm, 20.0 mm ∅, aceto-nitrile/water = 80:20, v = 15.0 mL / min, λ = 220 nm. The overall yield of each enantiomer ranged between 14-16%.

Complex S8. A flame dried Schlenk flask was charged with *n*-tetrabutylammonium acetate (13.9 mg, 0.046 mmol) and MeCN (3 mL). Complex *rac*-**7** (40 mg, 0.03 mmol) was added, followed by THF (1 mL) to improve the solubility. The mixture was stirred for 22 h at room temperature before it was concentrated. The residue was purified by flash chromatography (silica, toluene/acetonitrile 4:1 to 1:1) to afford the title complex as a green

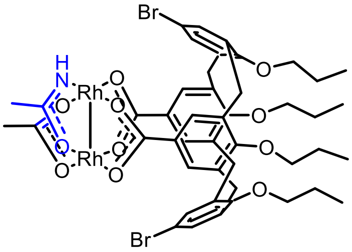

solid (20 mg, 52%). ^{1}H NMR (600 MHz, [D_8]-THF) δ 7.37 (d, *J* = 2.5 Hz, 1H), 7.35 (d, *J* = 2.6 Hz, 1H), 7.35 (d, *J* = 2.6 Hz, 1H), 7.34 (d, *J* = 2.6 Hz, 1H), 6.71 (s, 2H), 6.65 (d, *J* = 2.1 Hz, 1H), 6.59 (d, *J* = 2.1 Hz, 1H), 5.01 (s, 1H), 4.40 – 4.33 (m, 4H), 4.00 – 3.96 (m, 4H), 3.67 – 3.62 (m, 4H), 3.18 – 3.10 (m, 4H), 1.88 – 1.80 (m, 8H), 1.75 (s, 3H), 1.67 (s, 3H), 1.08 (t, *J* = 7.4 Hz, 3H), 1.08 (t, *J* = 7.4 Hz, 3H), 0.82 (t, *J* = 7.5 Hz, 3H), 0.81 (t, *J* = 7.5 Hz, 3H). ^{13}C NMR (151 MHz, [D_8]-THF) δ 188.71, 185.28, 184.20, 183.25, 158.97, 158.92, 158.26, 158.23, 140.32, 140.26, 140.21, 140.18, 132.66 – 132.61 (m), 132.57 (d, *J* = 2.7 Hz), 132.47 (d, *J* = 4.4 Hz), 130.16, 129.92, 129.66, 129.37, 128.34, 127.78, 115.13, 115.00, 77.70 (d, *J* = 3.9 Hz), 77.43, 77.36, 31.61 (dd, *J* = 4.8, 2.2 Hz), 24.34 (d, *J* = 1.7 Hz), 23.67, 23.62, 23.46, 22.95, 11.15 (d, *J* = 1.9 Hz), 9.95 (d, *J* = 2.6 Hz). IR (ATR): ṽ = 2961, 2925, 2875, 1599, 1582, 1551, 1462, 1400, 1384, 1293, 1207, 1111, 1003, 960, 770 cm^{-1}. HRMS (ESI +): *m/z calcd.* for $C_{46}H_{52}Br_2NO_{11}Rh_2$ $[M+H]^+$: 1158.00117; *found:* 1157.99987.

cis-Selective Formation of Stannylated Cyclopropanes

General Procedure for *cis*-Selective Cyclopropanations. A flame-dried Schlenk flask equipped with a magnetic stir bar was charged with complex ***P*-7** (0.5 mol%). The respective olefin (5 equiv.) and CH_2Cl_2 (3 mL) were added, followed by the *rapid* addition (< 1 min) of a solution of α-stannyl-α-diazoacetate **2** (55mg, 0.2 mmol, 1 equiv.) in CH_2Cl_2 (1 mL). The resulting mixture was stirred at room temperature for 4 h before it was concentrated under reduced pressure. The d.r. of the crude material was determined by ^{1}H NMR. The residue was then purified by flash chromatography. If the diastereomers were separable under these conditions, only the major diastereomer was isolated.

The corresponding racemic compounds were prepared analogously using $Rh_2(esp)_2$ (1 mol%)[28] as the catalyst.

The following compounds were prepared according to the general procedure described above:

Compound 3a. The crude matrial was purified by flash chromatography (silica, *n*-pentane/*tert*-butyl

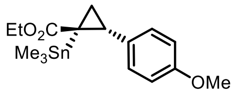

methyl ether 30:1) to afford the title compound as a colorless oil (43.5 mg, 58%, d.r. = 10:1 (*cis*), 98% *ee*). $[\alpha]_D^{20}$ = +46.9 (c = 1.9, $CHCl_3$). The optical purity was determined by HPLC (Chiralpak IG-3, 4.6 mm ∅, *n*-heptan/2-propanol = 98:2, v = 1.0 mL/min, λ =230 nm): 3.48 min (major) and 3.90 min (minor).

The spectral data matched those previously reported in the literature.[25]

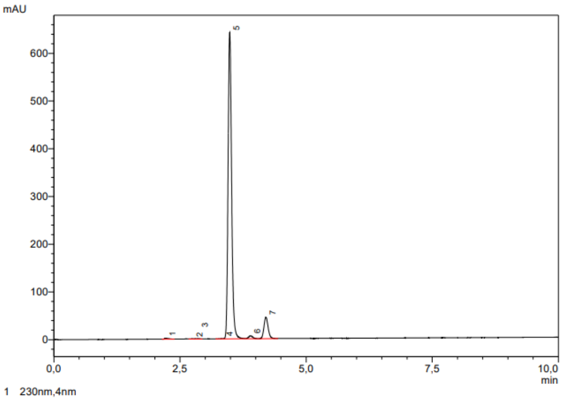

PDA Ch1 230nm

Peak #	Ret. Time	Area %	Name
1	2,22	0,32	
2	2,76	0,10	
3	2,85	0,15	
4	3,35	0,11	
5	3,48	90,63	1. Enantiomer 97.7 % ee
6	3,90	1,08	2. Enantiomer
7	4,20	7,60	
Total		100,00	

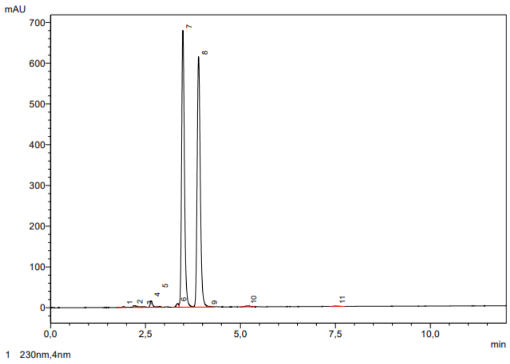

PDA Ch1 230nm

Peak #	Ret. Time	Area %	Name
1	1,92	0,12	
2	2,19	0,47	
3	2,45	0,15	
4	2,65	0,96	
5	2,86	0,17	
6	3,34	0,55	
7	3,48	48,29	1. Enantiomer Diastereomer 1
8	3,90	48,68	2. Enantiomer Diastereomer 1
9	4,15	0,16	
10	5,19	0,28	1. Enantiomer Diastereomer 2
11	7,52	0,16	2. Enantiomer Diastereomer 2
Total		100,00	

HPLC traces of **3a** (left) and the corresponding racemate (right).

Compound 3b. The crude product was purified by flash chromatography (silica, *n*-pentane/*tert*-butyl

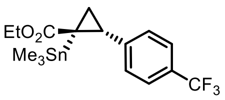

methyl ether 30:1) to give the title compound as a colorless oil (54.4 mg, 64%, d.r. = 7:1, 98% *ee*). $[\alpha]_D^{20}$ = +32.1 (c = 1.3, $CHCl_3$). The optical purity was determined by HPLC (Chiralpak IM-3, 4.6 mm ∅, methanol/water = 80:20, v = 1.0 mL/min, λ =220 nm): 7.09 min (minor) and 7.85 min (major). ^1H NMR (600 MHz, $CDCl_3$) δ 7.55 – 7.51 (m, 2H), 7.37 – 7.33 (m, 2H), 4.18 – 4.12 (m, 2H), 2.77 (dd, *J* = 8.8, 6.4 Hz, 1H), 1.78 (dd, *J* = 8.8, 4.3 Hz, 1H), 1.35 (dd, *J* = 6.4, 4.3 Hz, 1H), 1.28 (t, *J* = 7.1 Hz, 3H), –0.18 (s, 9H). ^{13}C NMR (151 MHz, $CDCl_3$) δ 176.9, 144.2 (q, *J* = 1.4 Hz), 129.9, 129.3 (q, *J* = 32.4 Hz), 125.3 (q, *J* = 3.8 Hz), 124.3 (q, *J* = 271.9 Hz), 61.2, 30.3, 21.2, 16.5, 14.4, –8.4. ^{119}Sn NMR (224 MHz, $CDCl_3$) δ 5.94. ^{19}F NMR (565 MHz, $CDCl_3$) δ –62.43. IR (ATR): ṽ = 1709, 1323, 1226, 1163, 1122, 1110, 1066, 1017, 843, 768, 529 cm^{-1}. HRMS (ESI^+) *m/z calcd.* for $C_{16}H_{21}F_3O_2SnNa$ $[M+Na]^+$: calcd: 445.04078; *found:* 445.04121.

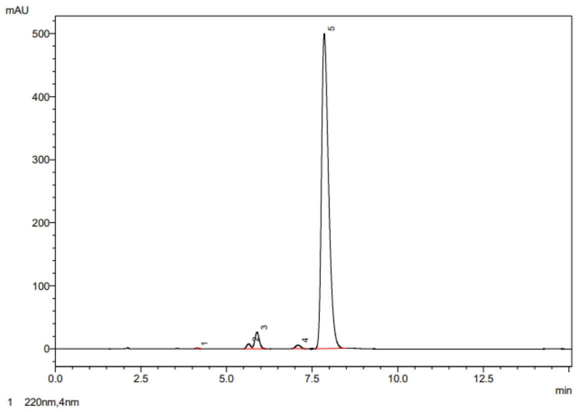

PDA Ch1 220nm

Peak #	Ret. Time	Area %	Name
1	4.14	0.11	
2	5.65	0.87	1st enantiomer trans
3	5.89	3.22	2nd enantiomer trans
4	7.09	0.82	1st enantiomer cis
5	7.85	94.97	2nd enantiomer cis
Total		100.00	

= 98.3 % ee

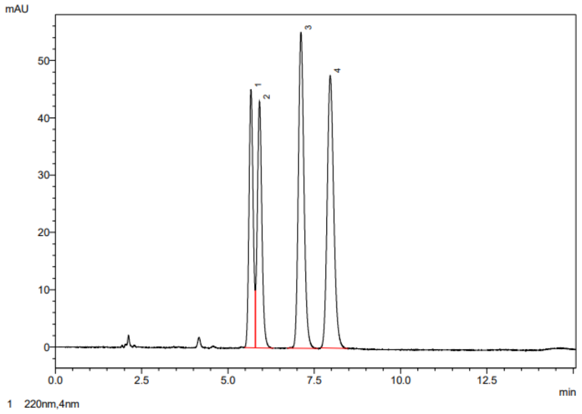

PDA Ch1 220nm

Peak #	Ret. Time	Area %	Name
1	5.67	18.58	1st enantiomer trans
2	5.91	19.71	2nd enantiomer trans
3	7.12	30.76	1st enantiomer cis
4	7.96	30.94	2nd enantiomer cis
Total		100.00	

HPLC traces of **3b** (left) and the corresponding racemate (right).

Compound 3c. The crude product was purified by flash chromatography (silica, *n*-pentane/*tert*-butyl methyl ether 30:1) to give the title compound as a colorless oil (57 mg, 71% (contains *trans*-diastereomer), d.r. = 7:1, 96% *ee*). $[\alpha]_D^{20}$ = +40.3 (c = 1.5, $CHCl_3$).

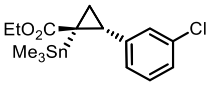

The optical purity was determined by 2D-HPLC: 1st dimension: (50 mm Eclipse Plus C18, 4.6 mm ∅, methanol/water 80:20, v = 1.0 mL/min, 308 K, λ =220 nm): 4.63-4.67 min; 2nd dimension: (Chiralpak OJ-3R, 4.6 mm ∅, methanol/water gradient = 70:30 to 95:5 in 10 min, v = 1.0 mL/min, λ = 220 nm): 8.82 min (major) and 9.12 min (minor).

The spectral data matched those previously reported in the literature.[29]

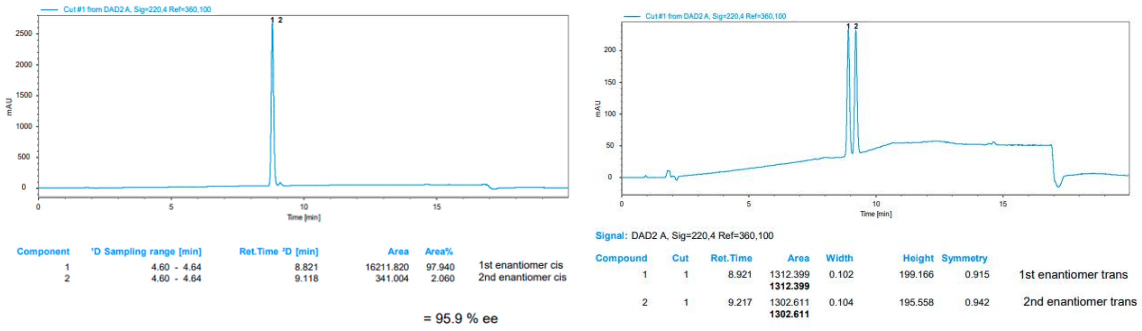

HPLC traces of **3c** (left) and the corresponding racemate (right).

Compound 3d. The crude material was purified by flash chromatography (silica, *n*-pentane/*tert*-butyl methyl ether 30:1) to give the title compound as a colorless oil (50 mg, 66%, d.r. = 9:1, 98% *ee*). The optical purity was determined by HPLC (Chiralpak OJ-3R, 4.6 mm ∅, methanol/water gradient = 70:30 to 95:5 in 10 min, v = 1.0 mL/min, λ =220 nm): 9.16 min (major) and 10.45 min (minor). $[\alpha]_D^{20}$ = –43.7 (c = 1.0, $CHCl_3$). ^1H NMR (400 MHz, $CDCl_3$) δ 7.41 – 7.31 (m, 1H), 7.24 – 7.12 (m, 2H), 7.11 – 7.08 (m, 1H), 4.25 – 4.08 (m, 2H), 2.73 (ddd, *J* = 8.6, 6.6, 0.8 Hz, 1H), 1.79 (dd, *J* = 8.6, 4.3 Hz, 1H), 1.41 (dd, *J* = 6.6, 4.3 Hz, 1H), 1.28 (t, *J* = 7.1 Hz, 3H), –0.17 (s, 9H). ^{13}C NMR (101 MHz, $CDCl_3$) δ 176.9, 137.8, 136.9, 129.6, 129.4, 128.3, 126.5, 61.0, 29.7, 21.3, 16.4, 14.5, –8.4. ^{119}Sn NMR (149 MHz, $CDCl_3$) δ 6.59. IR (ATR): ṽ = 1708, 1442, 1372, 1240, 1222, 1123, 1052, 758, 741, 528 cm^{-1}. HRMS (ESI^+) *m/z calcd.* for $C_{15}H_{21}O_2ClSnNa$ $[M+Na]^+$: calcd: 411.01442; *found:* 411.01419.

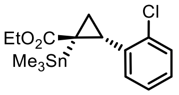

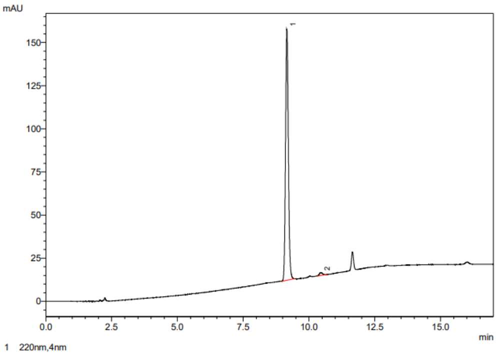

1 220nm,4nm

PDA Ch1 220nm

Peak #	Ret. Time	Area %	Name
1	9.16	98.75	1st enantiomer cis
2	10.45	1.25	2nd enantiomer cis
Total		100.00	

= 98 % ee

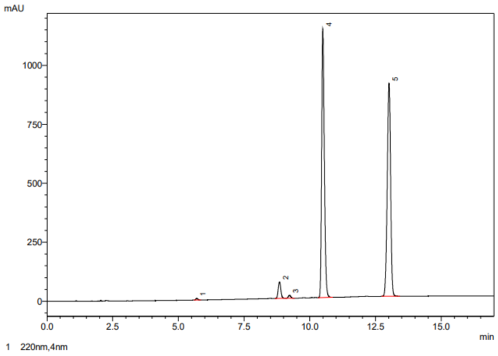

1 220nm,4nm

PDA Ch1 220nm

Peak #	Ret. Time	Area %	Name
1	5.70	0.22	
2	8.85	2.92	
3	9.23	0.50	1st enantiomer cis
4	10.49	48.51	1st enantiomer trans + 0.5 % 2nd enantiomer cis
5	13.01	47.85	2nd enantiomer trans
Total		100.00	

HPLC traces of **3d** (left) and the corresponding racemate (right).

Compound 3e. The crude material was purified by flash chromatography (silica, *n*-pentane/*tert*-butyl methyl ether 30:1) to give the title compound as a colorless oil (51.2 mg, 71%, d.r. = 15:1, 97% *ee*). $[\alpha]_D^{20}$ = +127.3 (c = 1.1, $CHCl_3$). The optical purity was determined by HPLC (Chiralpak OJ-3R, 4.6 mm ∅, methanol/water gradient = 60:40 to 80:20 in 10 min, v = 1.0 mL/min, λ =220 nm): 8.82 min (major) and 9.12 min (minor).

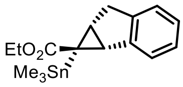

The spectral data matched those previously reported in the literature.[25]

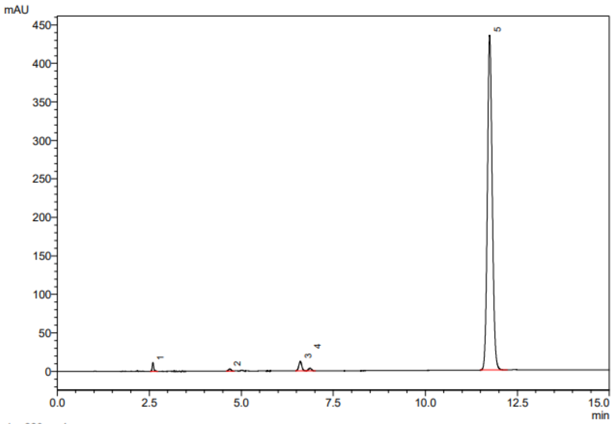

1 220nm,4nm

PDA Ch1 220nm

Peak #	Ret. Time	Area %	Name
1	2.60	0.78	
2	4.69	0.31	
3	6.60	1.71	1st enantiomer
4	6.87	0.46	
5	11.75	96.74	2nd enantiomer
Total		100.00	

= 96.5 % ee

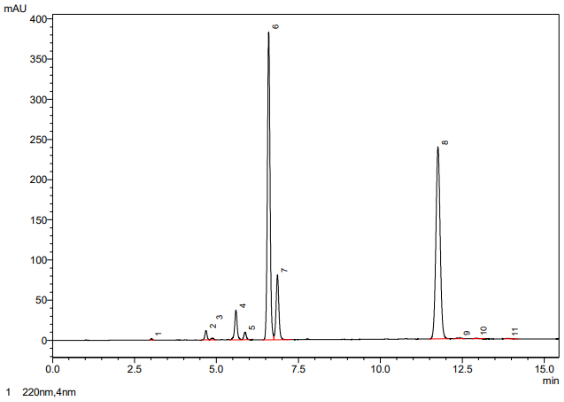

1 220nm,4nm

PDA Ch1 220nm

Peak #	Ret. Time	Area %	Name
1	3.02	0.10	
2	4.68	1.02	
3	4.87	0.22	
4	5.59	3.81	
5	5.87	0.93	
6	6.59	42.34	1st enantiomer
7	6.86	9.21	
8	11.76	42.00	2nd enantiomer
9	12.43	0.19	
10	12.93	0.11	
11	13.89	0.07	
Total		100.00	

HPLC traces of **3e** (left) and the corresponding racemate (right).

Compound 3f. The crude product was purified by flash chromatography (silica, *n*-pentane/*tert*-butyl

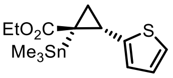

methyl ether 30:1) to furnish the title compound as a dark yellow oil (28 mg, 40%, d.r. = 10:1, 96% *ee*). The optical purity was determined by HPLC (Chiralpak OJ-3R, 4.6 mm ∅, acetonitrile/water = 55:45, v = 1.0 mL/min, λ =220 nm): 9.11 min (major) and 10.00 min (minor). $[\alpha]_D^{20}$ = +6.9 (c = 0.8, $CHCl_3$). ^1H NMR (400 MHz, $CDCl_3$) δ 7.12 (ddd, *J* = 5.2, 1.2, 0.6 Hz, 1H), 6.88 (dd, *J* = 5.1, 3.5 Hz, 1H), 6.80 (dt, *J* = 3.5, 1.2 Hz, 1H), 4.22 – 4.05 (m, 2H), 2.71 (ddd, *J* = 8.8, 5.8, 1.0 Hz, 1H), 1.81 (dd, *J* = 8.8, 3.9 Hz, 1H), 1.32 (dd, *J* = 6.1, 4.0 Hz, 1H), 1.28 (t, *J* = 7.1 Hz, 3H), –0.09 (s, 9H). ^{13}C NMR (101 MHz, $CDCl_3$) δ 176.7, 144.4, 126.7, 126.3, 124.6, 61.2, 24.7, 22.2, 18.7, 14.4, –8.5. ^{119}Sn NMR (149 MHz, $CDCl_3$) δ 4.26. IR (ATR): ṽ = 1707, 1367, 1236, 1209, 1125, 1082, 767, 695, 528, 512 cm^{-1}. HRMS (ESI^+) *m/z calcd.* for $C_{13}H_{20}O_2SSnNa$ $[M+Na]^+$: calcd: 383.00982; *found:* 383.00970.

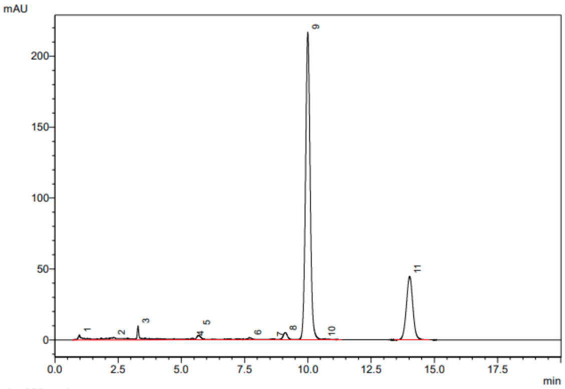

1 220nm,4nm

PDA Ch1 220nm

Peak #	Ret. Time	Area %	Name	
1	0.97	1.16		
2	2.32	2.11		
3	3.29	2.66		
4	5.44	0.47		
5	5.69	1.79		
6	7.72	0.40	1. Enantiomer trans	
7	8.61	0.38		
8	9.11	1.51	1. Enantiomer cis	ee = 95.7%
9	10.00	69.26	2. Enantiomer cis/trans	
10	10.63	0.28		
11	14.02	20.00	? no achiral separation!	the sample contains 0.76% of trans isomer
Total		100.00		

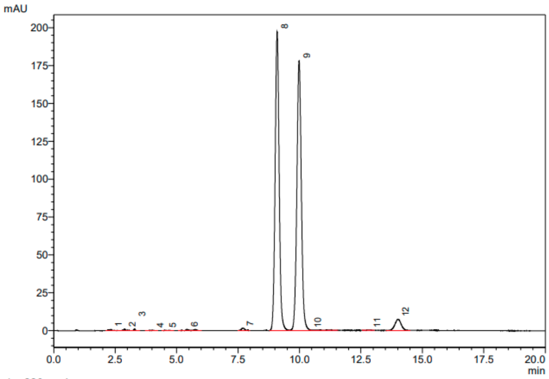

1 220nm,4nm

PDA Ch1 220nm

Peak #	Ret. Time	Area %	Name	
1	2.32	0.16		
2	2.88	0.15		
3	3.29	0.08		
4	4.03	0.04		
5	4.52	0.04		
6	5.43	0.23		
7	7.68	0.33	1. Enantiomer trans	
8	9.09	47.69	1. Enantiomer cis	
9	9.98	48.11	2. Enantiomer cis/trans	the sample contains 0.66% of trans isomer
10	10.43	0.26		
11	12.84	0.08		
12	14.01	2.83	?	
Total		100.00		

HPLC traces of **3f** (left) and the corresponding racemate (right).

Compound 3g. The crude product was purified by flash chromatography (silica, *n*-pentane/*tert*-butyl

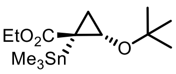

methyl ether 30:1) to give the title compound as a colorless oil (55 mg, 79%, d.r. > 20:1, 96% *ee*). $[\alpha]_D^{20}$ = +37.3 (c = 1.3, $CHCl_3$). The optical purity was determined by HPLC (Chiralpak OJ-3R, 4.6 mm ∅, methanol/water = 80:20, v = 1.0 mL/min, λ =210 nm): 3.63 min (major) and 4.19 min (minor).

The spectral data matched those previously reported in the literature.[25]

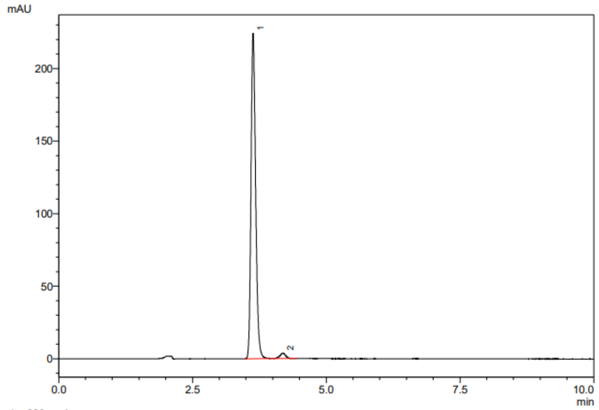

1 220nm,4nm

PDA Ch1 220nm

Peak #	Ret. Time	Area %	Name
1	3.63	97.97	1st enantiomer cis
2	4.19	2.03	2nd enantiomer cis
Total		100.00	

= 95.9 % ee

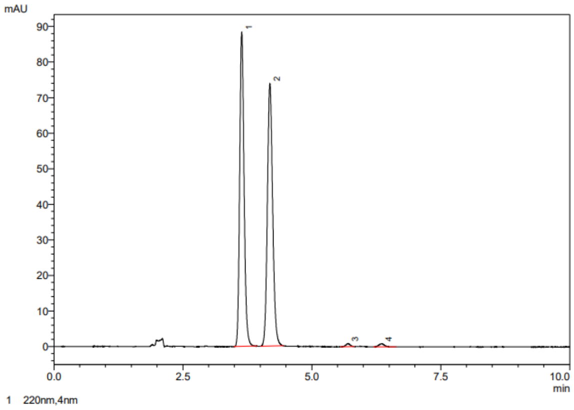

1 220nm,4nm

PDA Ch1 220nm

Peak #	Ret. Time	Area %	Name
1	3.64	49.37	1st enantiomer cis
2	4.19	49.58	2nd enantiomer cis
3	5.70	0.46	1st enantiomer trans ?
4	6.36	0.59	2nd enantiomer trans ?
Total		100.00	

HPLC traces of **3g** (left) and the corresponding racemate (right).

Compound 3h. The crude product was purified by flash chromatography (silica, *n*-pentane/EtOAc 12:1 to 10:1) to afford the title compound as a colorless oil (60 mg, 70%, d.r. > 20:1, 97% *ee*). $[\alpha]_D^{20}$ = +8.4 (c = 1.2, $CHCl_3$). The optical purity was determined by HPLC (Chiralpak OJ-3R, 4.6 mm ∅, methanol/water = 85:15, v = 1.0 mL/min, λ =220 nm): 4.73 min (major) and 6.29 min (minor).

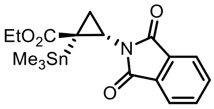

The spectral data matched those previously reported in the literature.[25]

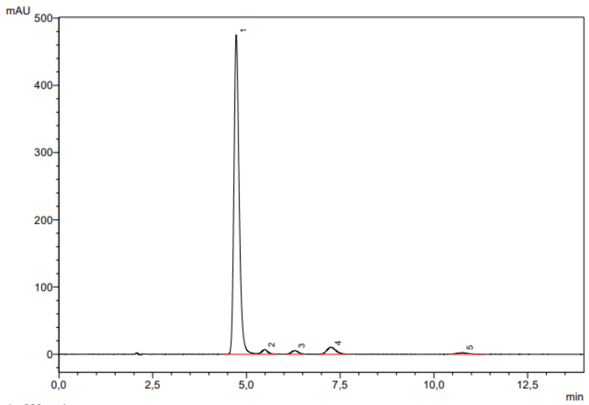

1 220nm,4nm

PDA Ch1 220nm

Peak #	Ret. Time	Area %	Name
1	4,73	92,29	1. Enantiomer Diastereomer 2 96.9 % ee
2	5,49	1,66	1. Enantiomer Diastereomer 1
3	6,29	1,43	2. Enantiomer Diastereomer 2
4	7,25	3,58	2. Enantiomer Diastereomer 1
5	10,77	1,05	
Total		100,00	

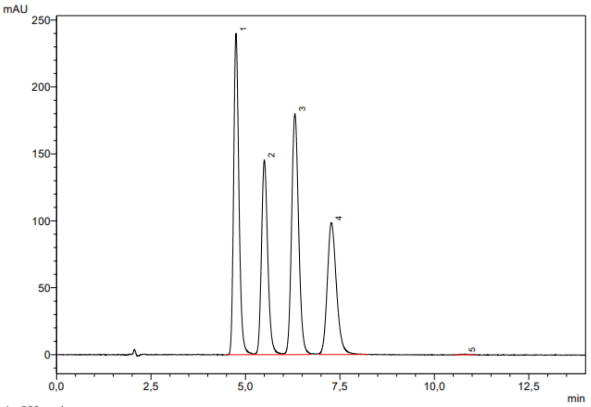

1 220nm,4nm

PDA Ch1 220nm

Peak #	Ret. Time	Area %	Name
1	4,75	28,94	1. Enantiomer Diastereomer 2
2	5,50	20,98	1. Enantiomer Diastereomer 1
3	6,31	29,13	2. Enantiomer Diastereomer 2
4	7,28	20,82	2. Enantiomer Diastereomer 1
5	10,81	0,12	
Total		100,00	

HPLC traces of **3h** (left) and the corresponding racemate (right).

Compound 3i. The crude product was purified by flash chromatography (silica, *n*-pentane/*tert*-butyl methyl ether 30:1) to give the title compound as a colorless oil (58.8 mg, 80% (contains *trans* diastereomer), d.r. = 3:1, 93% *ee*). $[\alpha]_D^{20}$ = +29.2 (c = 1.3, $CHCl_3$). ^1H NMR (400 MHz, $CDCl_3$) δ 4.18 – 3.96 (m, 2H), 1.56 – 1.43 (m, 1H), 1.33 (dddd, *J* = 11.8, 9.1, 6.2, 3.1 Hz, 1H), 1.23 (t, *J* = 7.1 Hz, 3H), 1.16 – 1.05 (m, 1H), 0.57 (dt, *J* = 6.2, 3.0 Hz, 1H), 0.15 (s, 9H), 0.03 (s, 9H). ^{13}C NMR (101 MHz, $CDCl_3$) δ 177.7, 60.7, 24.3, 21.6, 20.3, 19.9, 14.4, –1.4, –7.6. ^{119}Sn NMR (149 MHz, $CDCl_3$) δ 19.67 (minor diastereomer), –0.45 (major diastereomer). IR (ATR): ṽ = 1710, 1248, 4219, 1175, 1130, 856, 837, 765, 695, 527, 512 cm^{-1}. HRMS (ESI^+) *m/z calcd.* for $C_{13}H_{28}O_2SiSnNa$ $[M+Na]^+$: calcd: 387.07727; *found:* 387.07709.

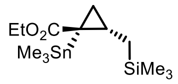

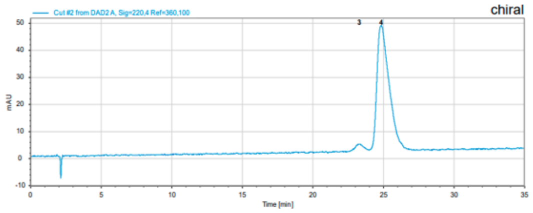

Component	1D Sampling range [min]	Ret.Time 2D [min]	Area	Area%
1	4.31 - 4.39	23.226	617.991	15.174
2	4.31 - 4.39	24.665	675.312	16.581
3	4.64 - 4.72	23.322	102.890	2.526
4	4.64 - 4.72	24.850	2676.579	65.719

ee = 92.6 %

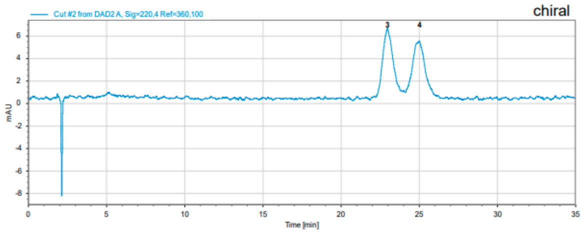

Signal: DAD2 A, Sig=220,4 Ref=360,100

Compound	Cut	Ret.Time	Area	Width	Height	Symmetry
3	2	22.984	274.336<br>**274.336**	0.757	6.039	0.819
4	2	25.024	282.715<br>**282.715**	0.942	5.002	1.210

HPLC traces of **3i** (left) and the corresponding racemate (right).

Other Cyclopropanations

Compound 10. An oven dried cooling Schlenk flask equipped with a magnetic stir bar was charged with catalyst ***M*-7** (1.0 mol%). Styrene (0.06 mL, 0.51 mmol) and *n*-pentane (1 mL) were added and the mixture was cooled to –40°C. At this temperature, a solution of 2,2,2-trichloroethyl 2-diazo-2-phenylacetate **9** (30 mg, 0.1 mmol) in *n*-pentane (2 mL) was added dropwise over 5 min and stirring was continued at –40°C for 5 h. The mixture was concentrated under reduced pressure and the residue was purified by flash chromatography (silica, *n*-pentane/*tert*-butyl methyl ether, 40:1) to give the title compound as a white oil (27 mg, 71%, dr > 20:1, 70% *ee*). $[\alpha]_D^{20}$ = –8.1 (c = 1.2, $CHCl_3$). The optical purity was determined by HPLC (Chiralcel OJ-3R, 4.6 mm ∅, acetonitrile/water= 60:40, v = 1.0 mL/min, λ =220 nm): 10.24 min (major) and 13.34 min (minor). ^1H NMR (600 MHz, $CDCl_3$) δ 7.14 – 7.12 (m, 3H), 7.09 – 7.06 (m, 5H), 6.82 – 6.79 (m, 2H), 4.84 (d, *J* = 12.0 Hz, 1H), 4.65 (d, *J* = 11.9 Hz, 1H), 3.22 (dd, *J* = 9.4, 7.4 Hz, 1H), 2.29 (dd, *J* = 9.4, 5.1 Hz, 1H), 2.02 (dd, *J* = 7.4, 5.1 Hz, 1H). ^{13}C NMR (151 MHz, $CDCl_3$) δ 172.3, 135.9, 133.8, 132.2, 128.3, 127.9, 127.8, 127.4, 126.7, 95.2, 74.5, 37.4, 34.0, 20.4.

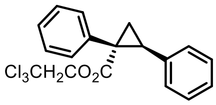

The spectral data matched those previously reported in the literature.[30]

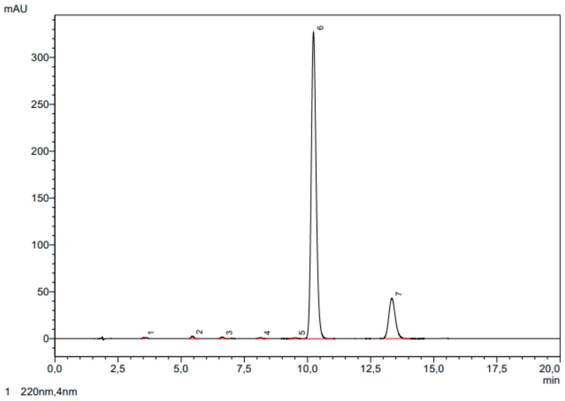

PDA Ch1 220nm

Peak #	Ret. Time	Area %	Name
1	3,53	0,13	
2	5,44	0,37	
3	6,63	0,29	
4	8,13	0,22	
5	9,52	0,23	
6	10,24	84,04	1. Enantiomer 70.2 % ee
7	13,34	14,73	2. Enantiomer
Total		100,00	

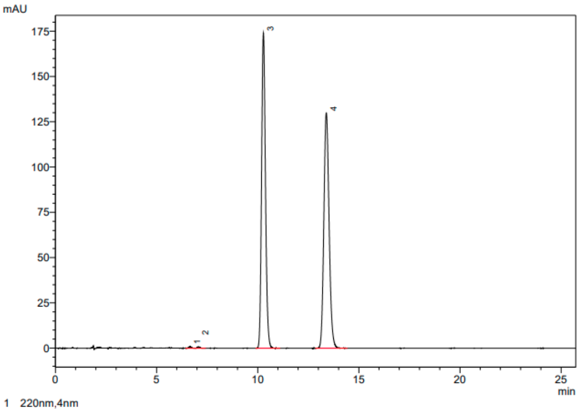

PDA Ch1 220nm

Peak #	Ret. Time	Area %	Name
1	6,65	0,16	
2	7,07	0,16	
3	10,28	49,84	1. Enantiomer
4	13,39	49,84	2. Enantiomer
Total		100,00	

HPLC traces of **10** (left) and the corresponding racemate (right).

Compound 12. An oven dried cooling Schlenk flask equipped with a magnetic stir bar was charged with

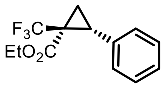

catalyst ***P*-7** (0.5 mol%). Styrene (0.13 mL, 1 mmol) and *n*-pentane (1 mL) were added and the mixture was cooled to –40°C. At this temperature, a solution of ethyl 3,3,3-trifluoro-2-diazopropionate **11** (36.4 mg, 0.2 mmol) in *n*-pentane (2 mL) was added dropwise over 5 min and stirring was continued at –40°C for 22 h. The mixture was concentrated under reduced pressure and the residue was purified by flash chromatography (silica, *n*-pentane/*tert*-butyl methyl ether, 40:1) to give the title compound as a colorless oil (33 mg, 63%, dr = 12:1), 95% *ee*). $[\alpha]_D^{20}$ = –2.6 (c = 1.0, $CHCl_3$). The optical purity was determined by HPLC (Chiralcel OJ-3R, 4.6 mm ∅, methanol/water = 80:20, v = 1.0 mL/min, λ =220 nm): 5.32 min (minor) and 7.45 min (major). ^1H NMR (600 MHz, $CDCl_3$) δ 7.33 – 7.21 (m, 5H), 3.92 – 3.82 (m, 2H), 2.95 (t, *J* = 9.0 Hz, 1H), 2.19 – 2.13 (m, 1H), 1.78 (dd, *J* = 9.7, 5.7 Hz, 1H), 0.88 (t, *J* = 7.1 Hz, 3H). ^{13}C NMR (151 MHz, $CDCl_3$) δ 165.2 (q, *J* = 1.1 Hz), 133.9, 129.3, 128.4, 127.7, 125.5 (q, *J* = 272.6 Hz), 61.6, 34.5 (q, *J* = 33.4 Hz), 29.3 (q, *J* = 1.9 Hz), 15.1 (q, *J* = 2.0 Hz), 13.7. ^{19}F NMR (565 MHz, $CDCl_3$) δ –66.9. IR (ATR): ṽ = 1737, 1395, 1372, 1334, 1315, 1224, 1198, 1146, 1121, 1079, 1026, 732, 697 cm^{-1}. HRMS (EI) m/z calcd. for $C_{13}H_{13}O_2F_3$ $[M]^+$: calcd: 258.08622; found: 258.08632.

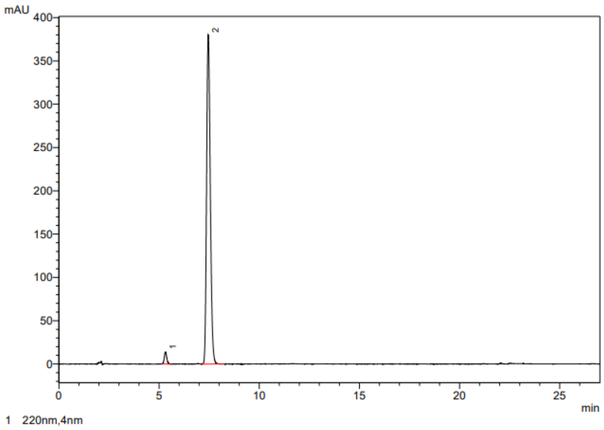

PDA Ch1 220nm

Peak #	Ret. Time	Area %	Name
1	5,32	2,38	1. Enantiomer Diastereomer 1
2	7,45	97,62	2. Enantiomer Diastereomer 1 95.2 % ee
Total		100,00	

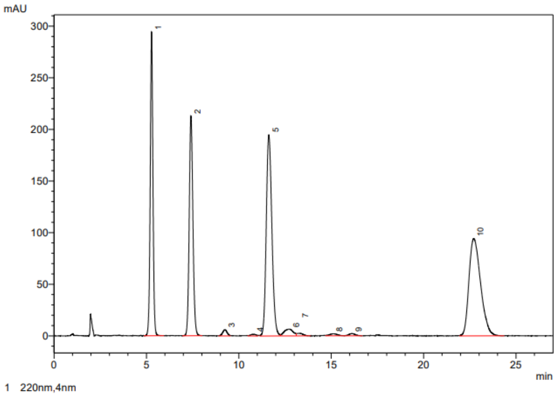

PDA Ch1 220nm

Peak #	Ret. Time	Area %	Name
1	5,28	20,23	1. Enantiomer Diastereomer 1
2	7,41	20,08	2. Enantiomer Diastereomer 1
3	9,24	0,69	
4	10,80	0,24	
5	11,62	28,03	1. Enantiomer Diastereomer 2
6	12,74	1,53	
7	13,23	0,40	
8	15,09	0,41	
9	16,12	0,36	
10	22,71	28,04	2. Enantiomer Diastereomer 2
Total		100,00	

HPLC traces of **12** (left) and of the corresponding racemate (right).

Spectra

Compound S1: ^{13}C NMR (101 MHz, $CDCl_3$):

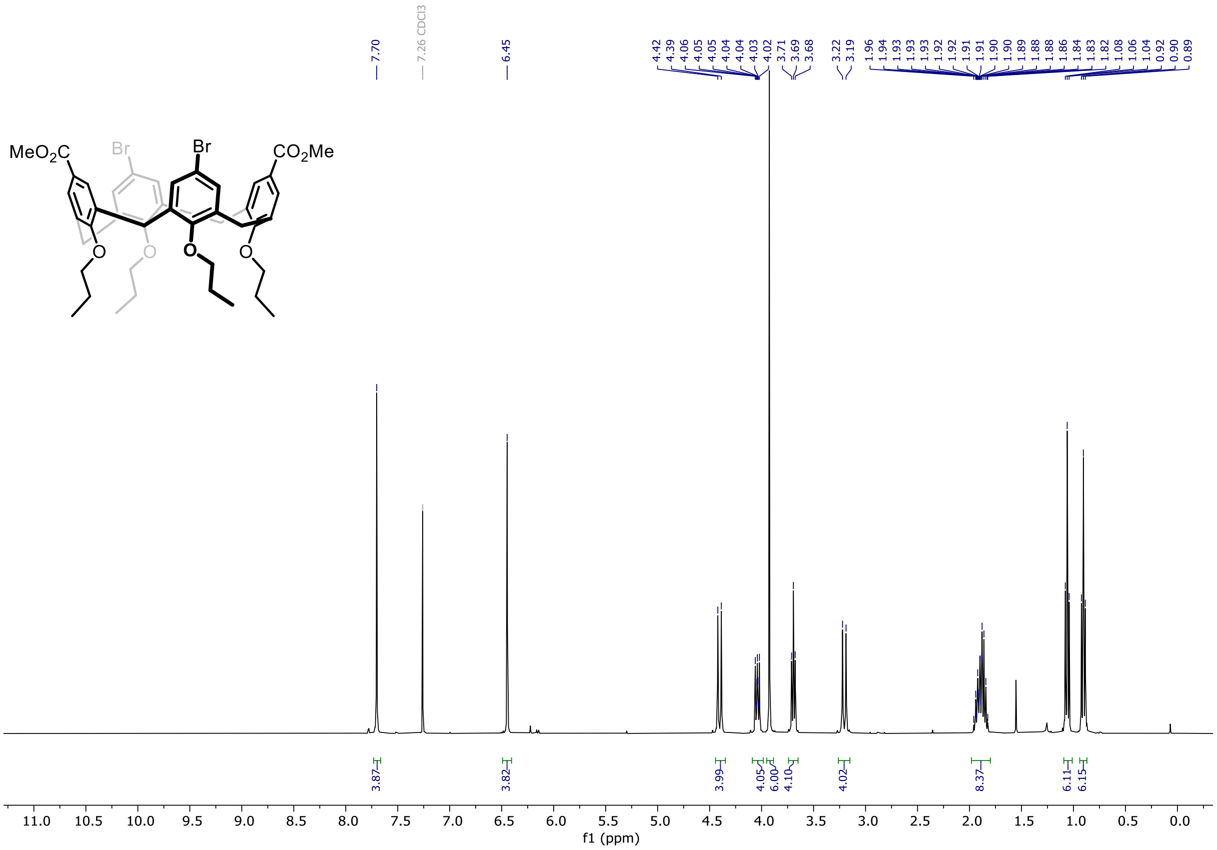

Compound S1: ^{13}C NMR (101 MHz, $CDCl_3$):

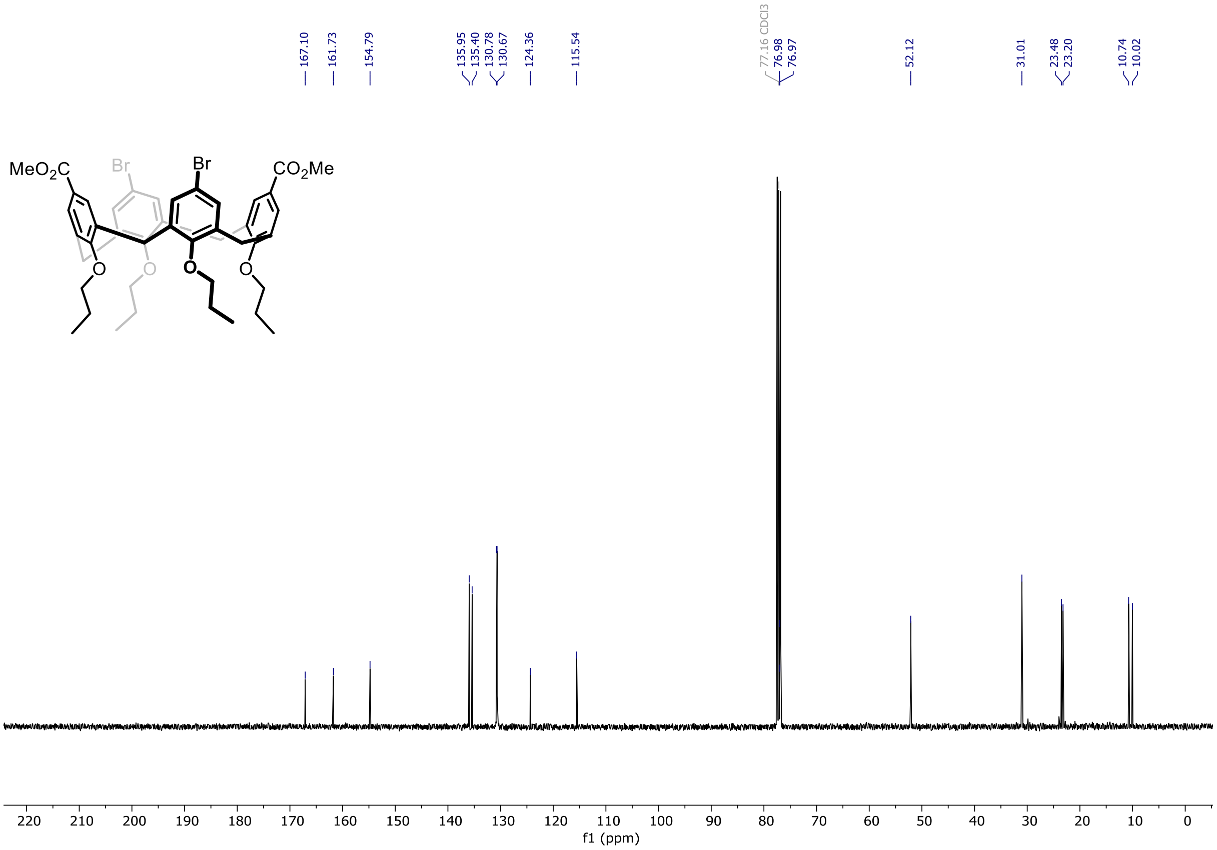

Compound S2: ^{1}H NMR (400 MHz, $CDCl_3$):

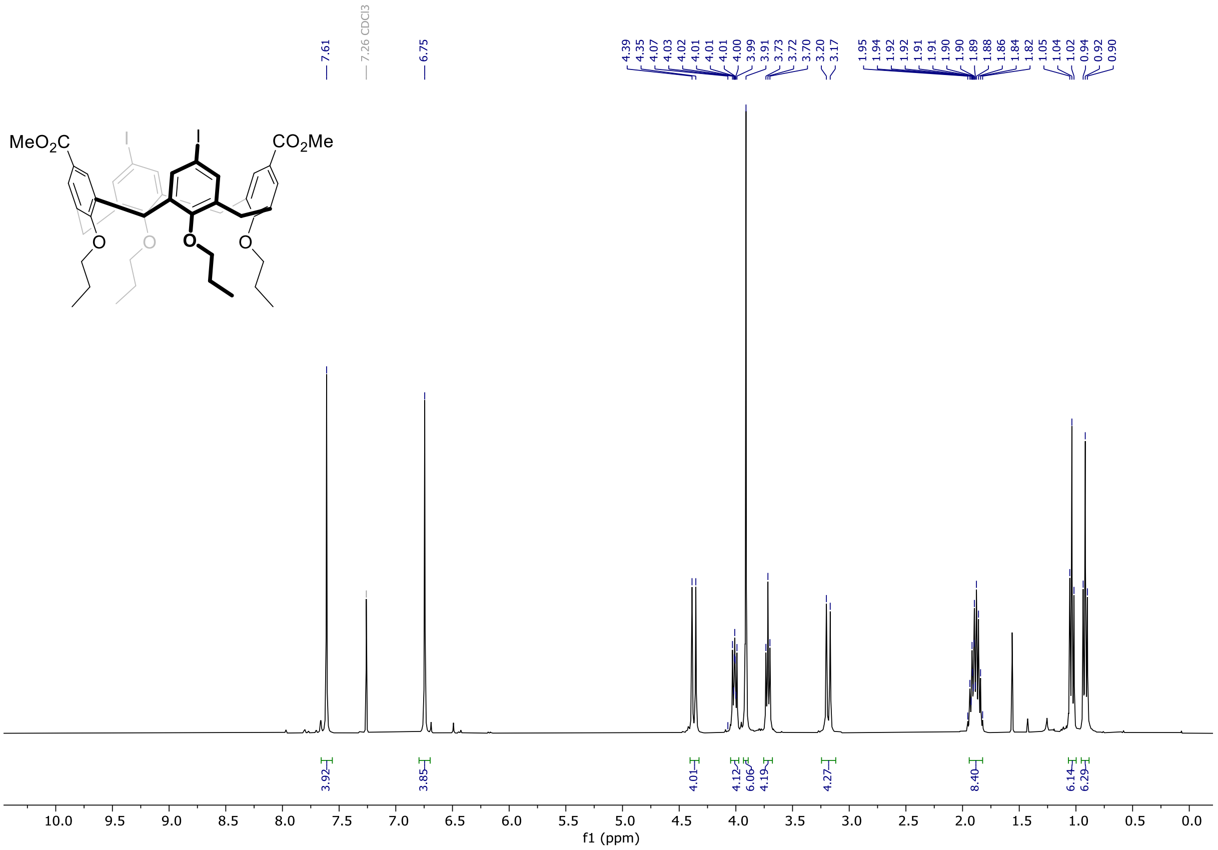

Compound S2: ^{13}C NMR (101 MHz, $CDCl_3$):

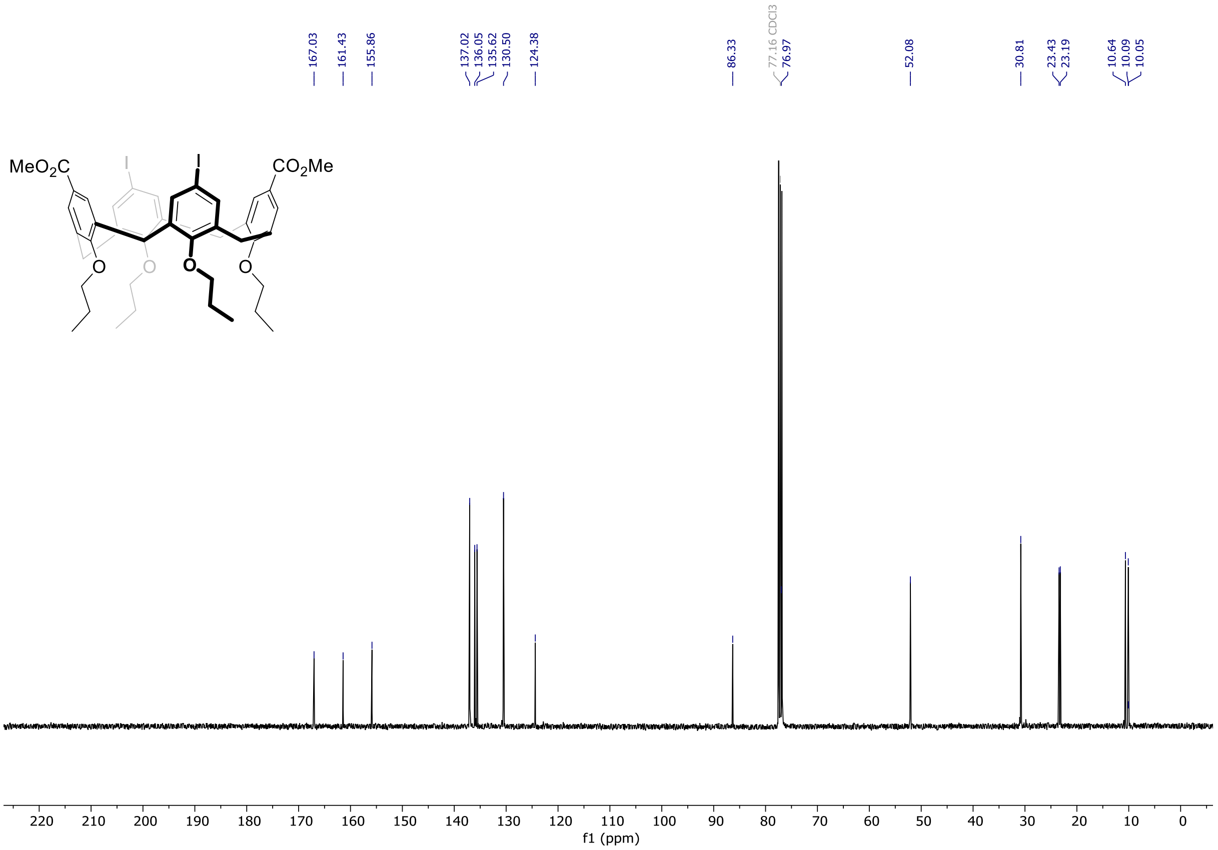

Compound S3: ^{1}H NMR (400 MHz, $CDCl_3$):

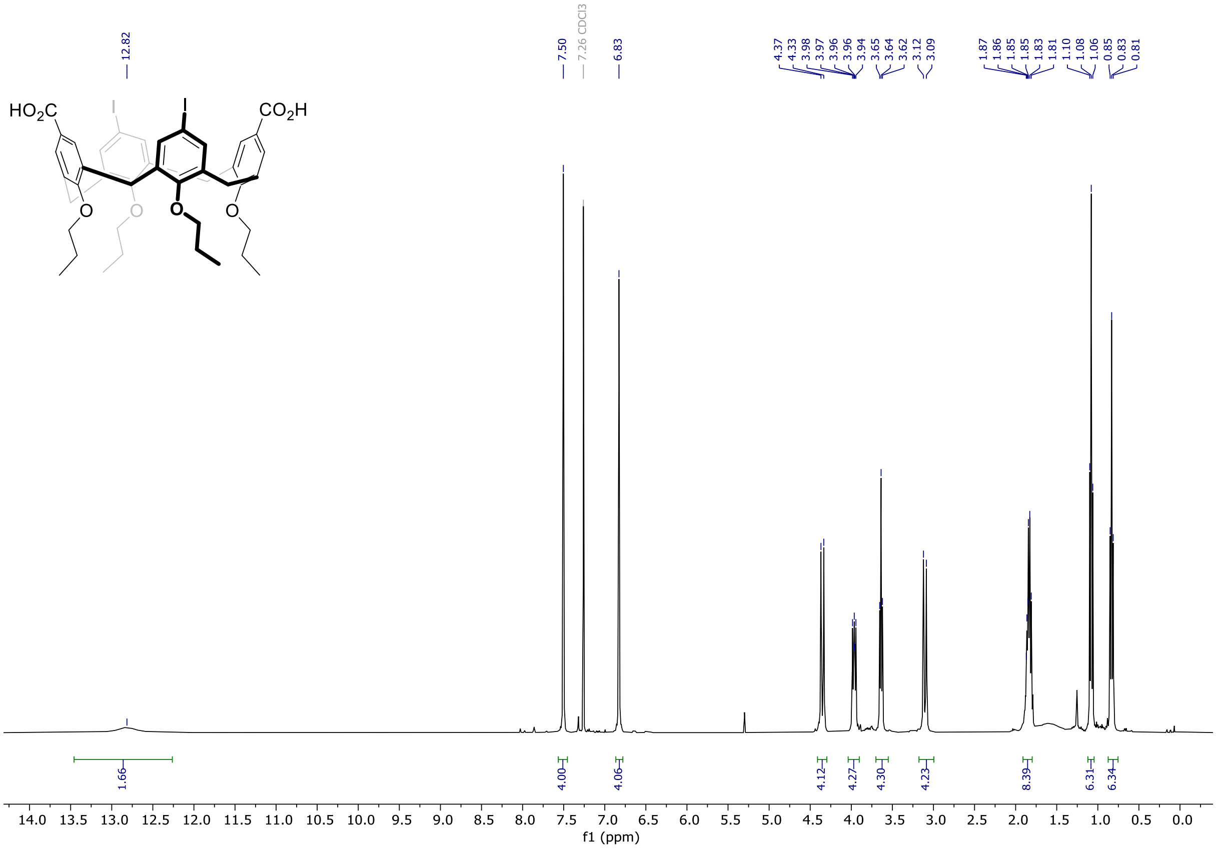

Compound S3: ^{13}C NMR (151 MHz, $CDCl_3$):

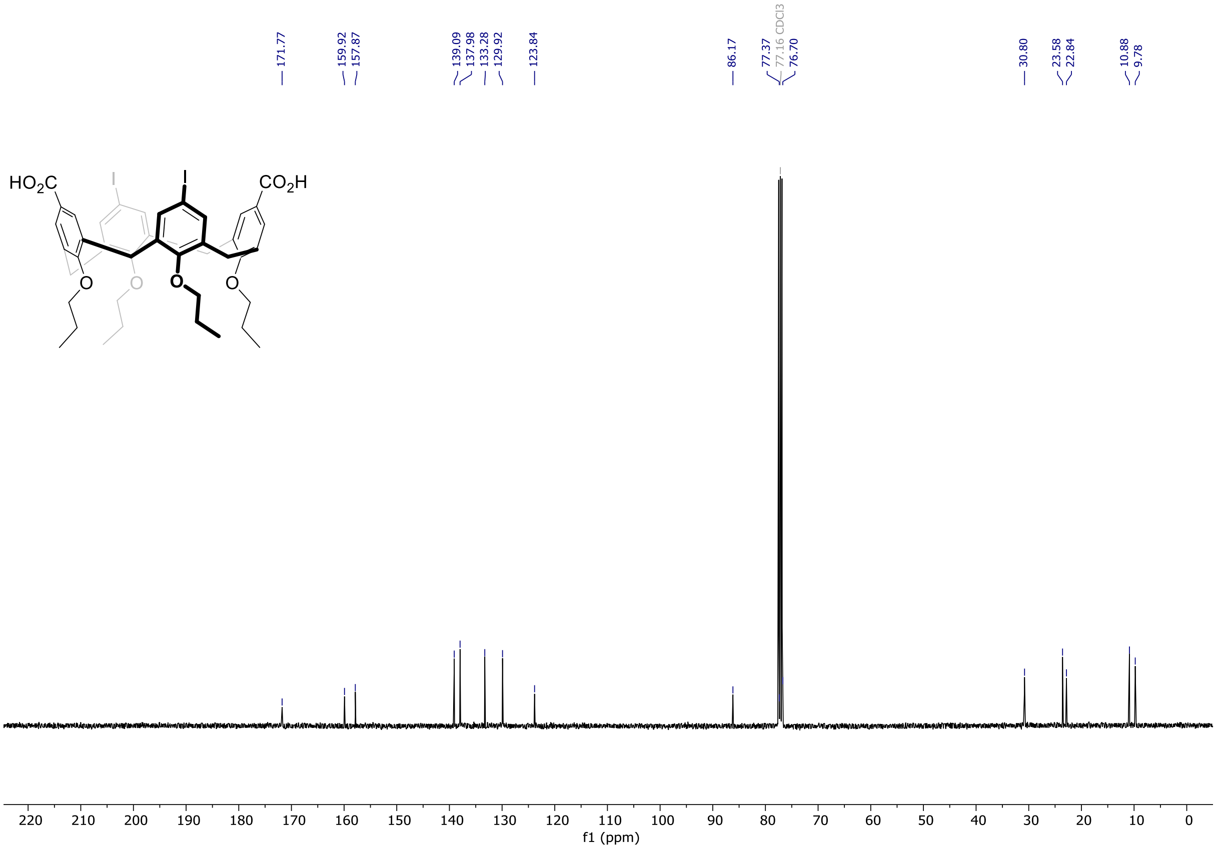

Complex S5: ^{1}H NMR (400 MHz, [D_8]-THF):

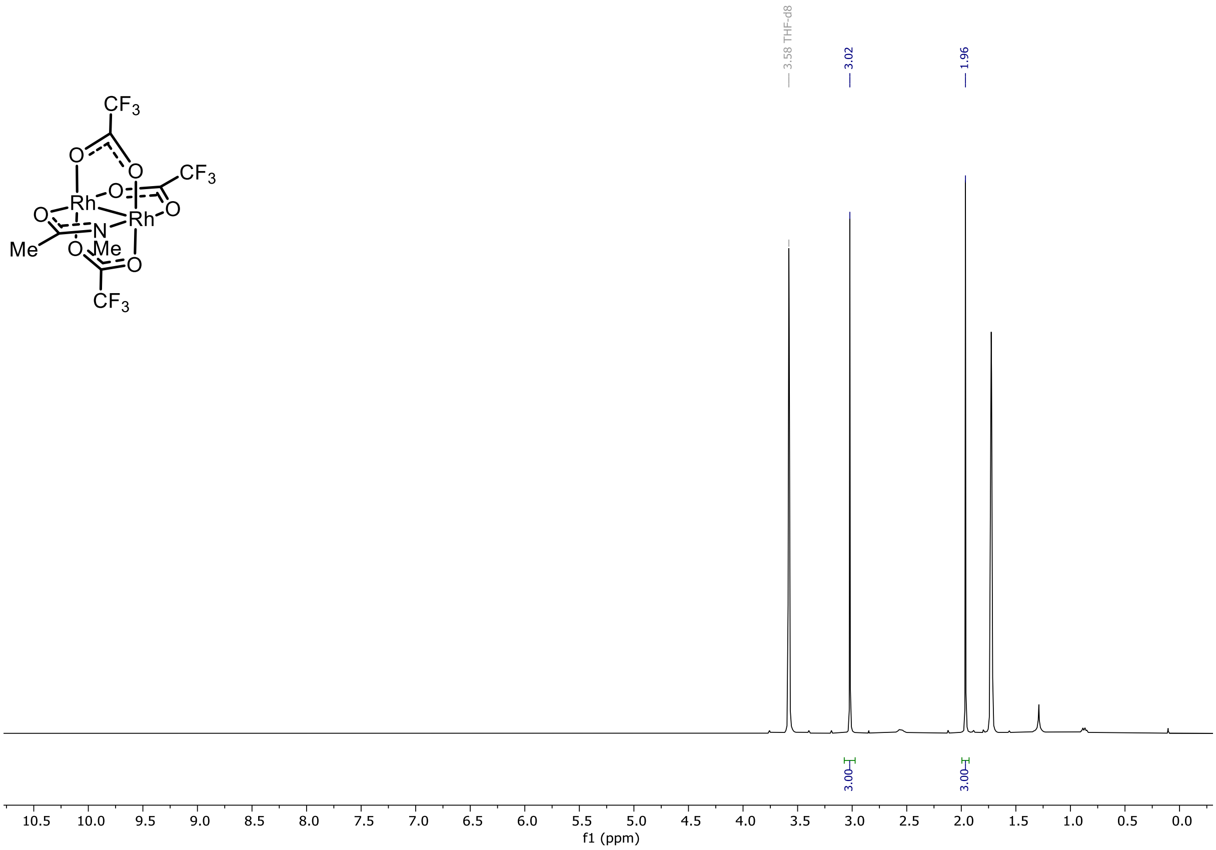

Complex S5: ^{13}C NMR (101 MHz, [D_8]-THF):

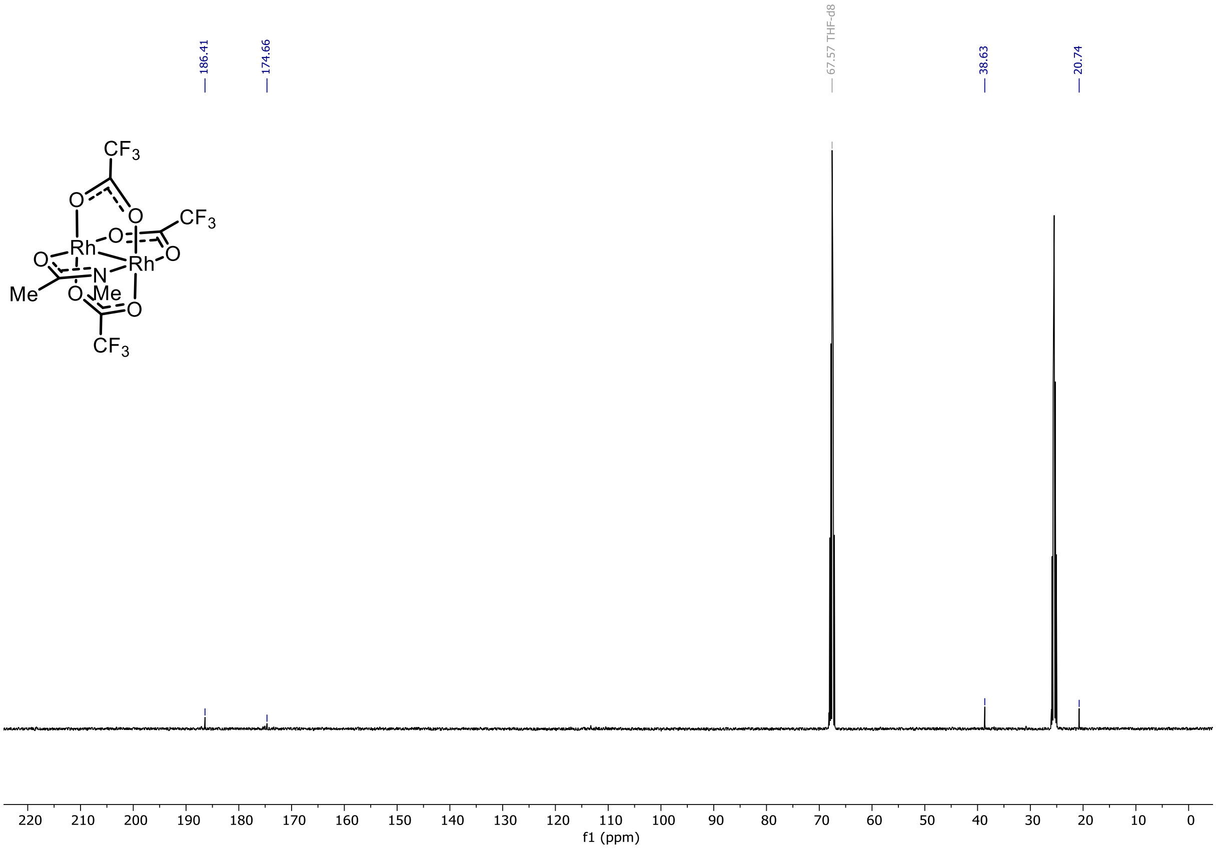

Complex S5: ^{19}F NMR (282 MHz, $CDCl_3$):

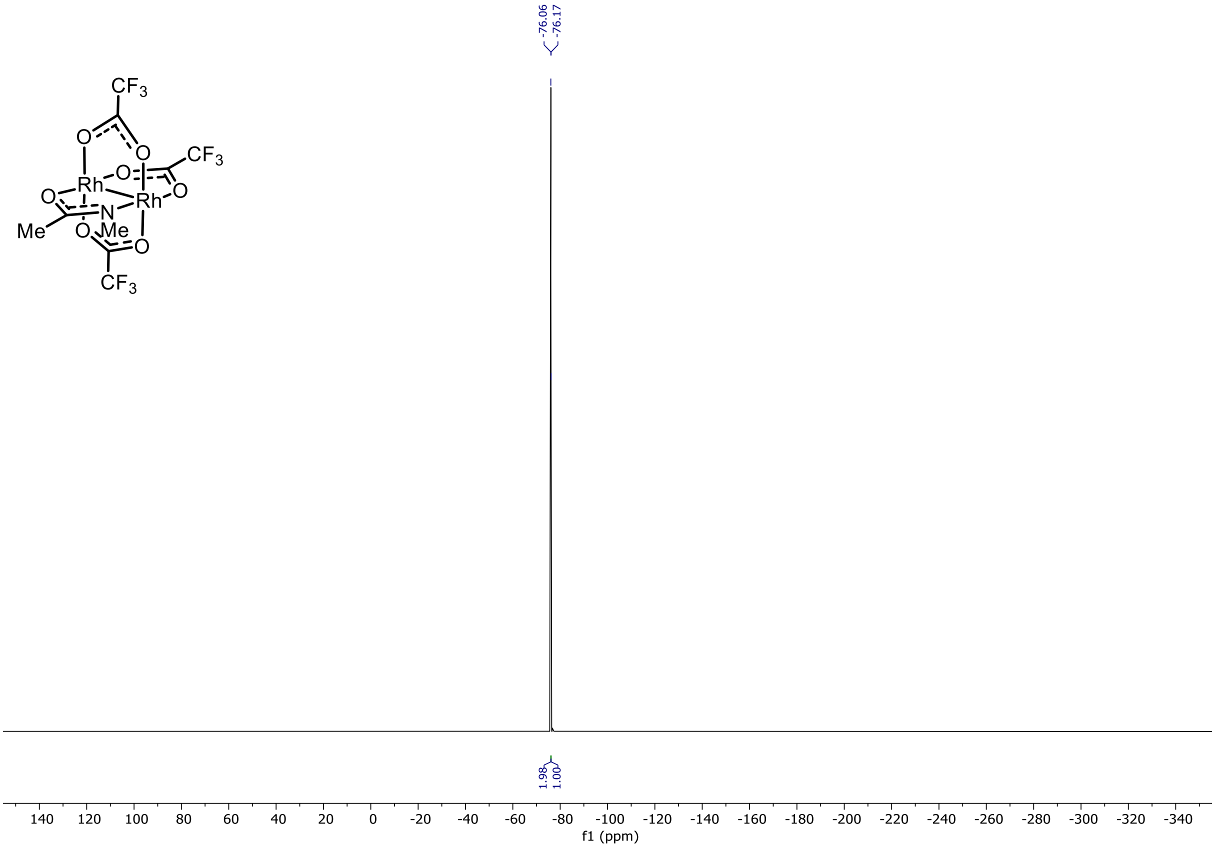

Complex 7: ^{1}H NMR (600 MHz, [D$_8$]-THF):

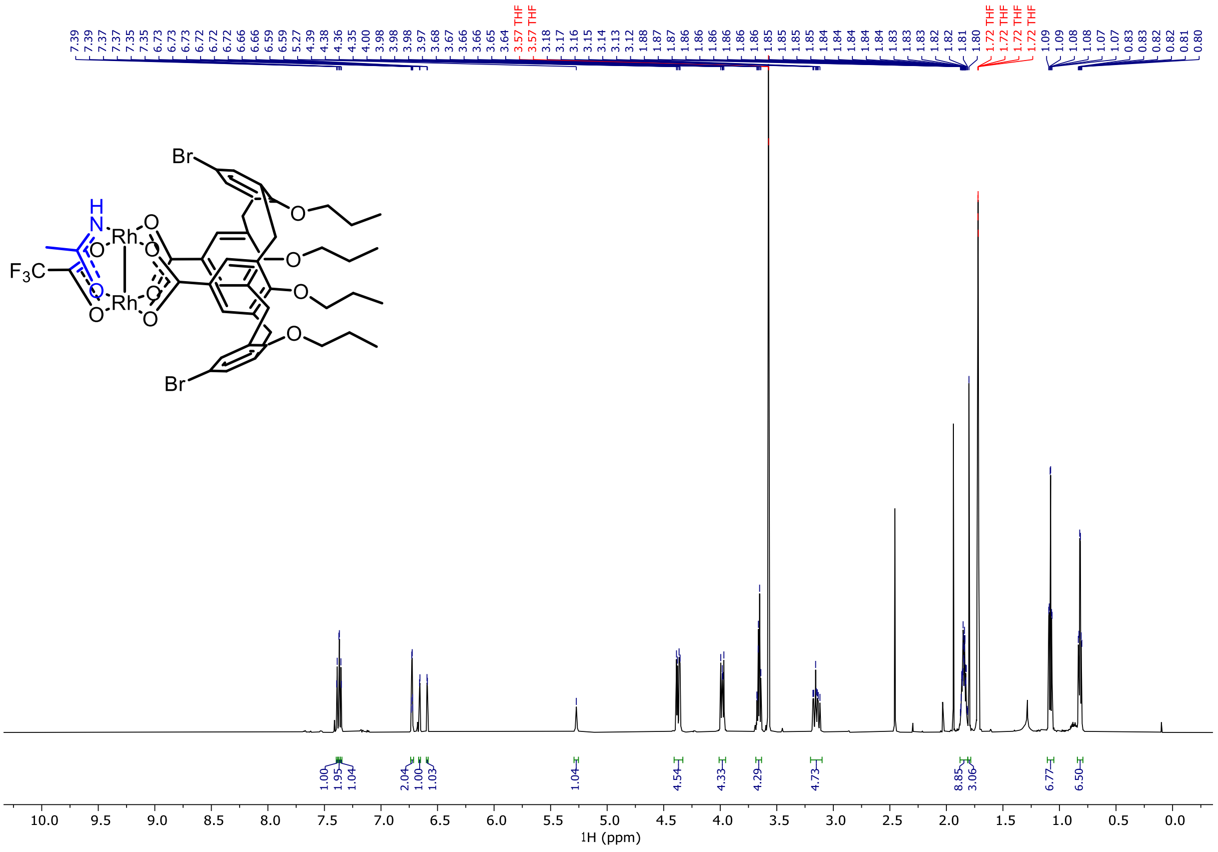

Complex 7: ^{13}C NMR (151 MHz, [D$_8$]-THF):

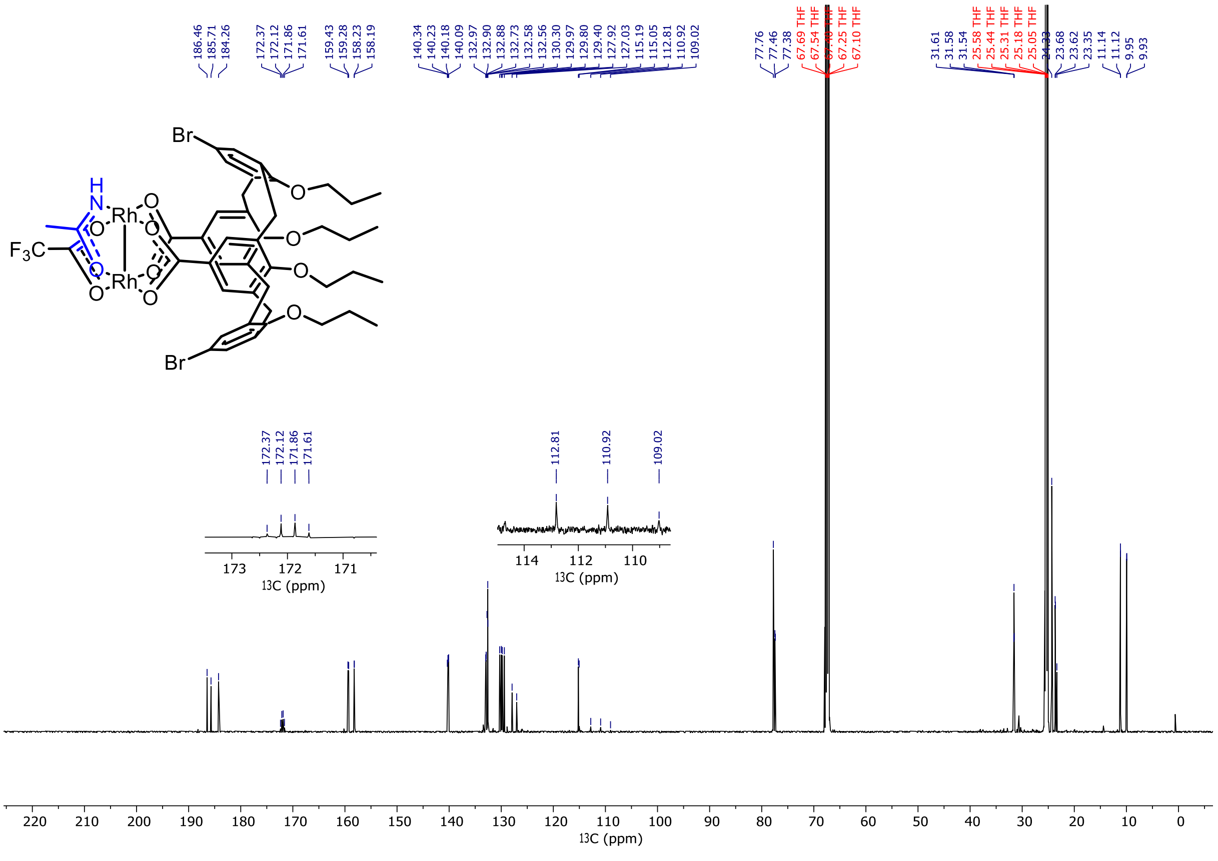

Complex 7: ^{19}F NMR (586 MHz, [D$_8$]-THF):

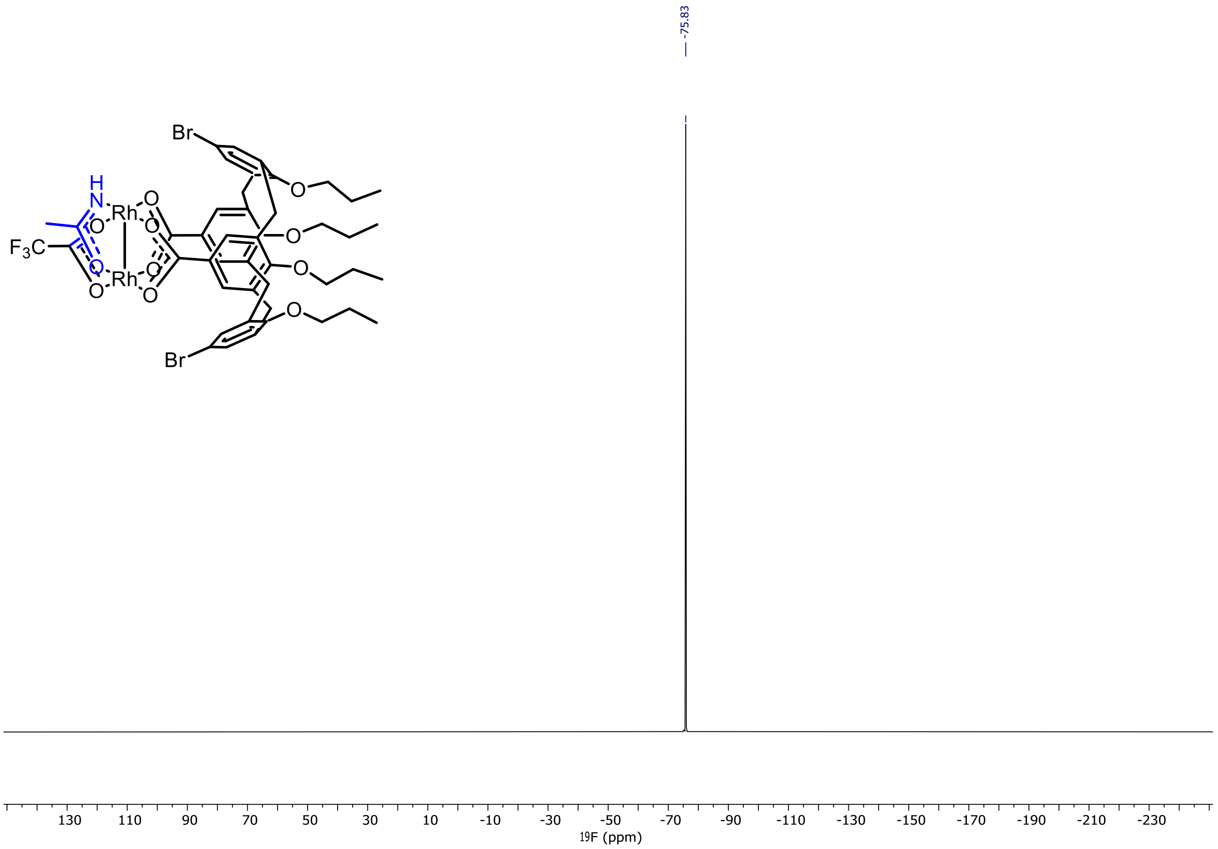

Complex 7: NOESY ([D$_8$]-THF):

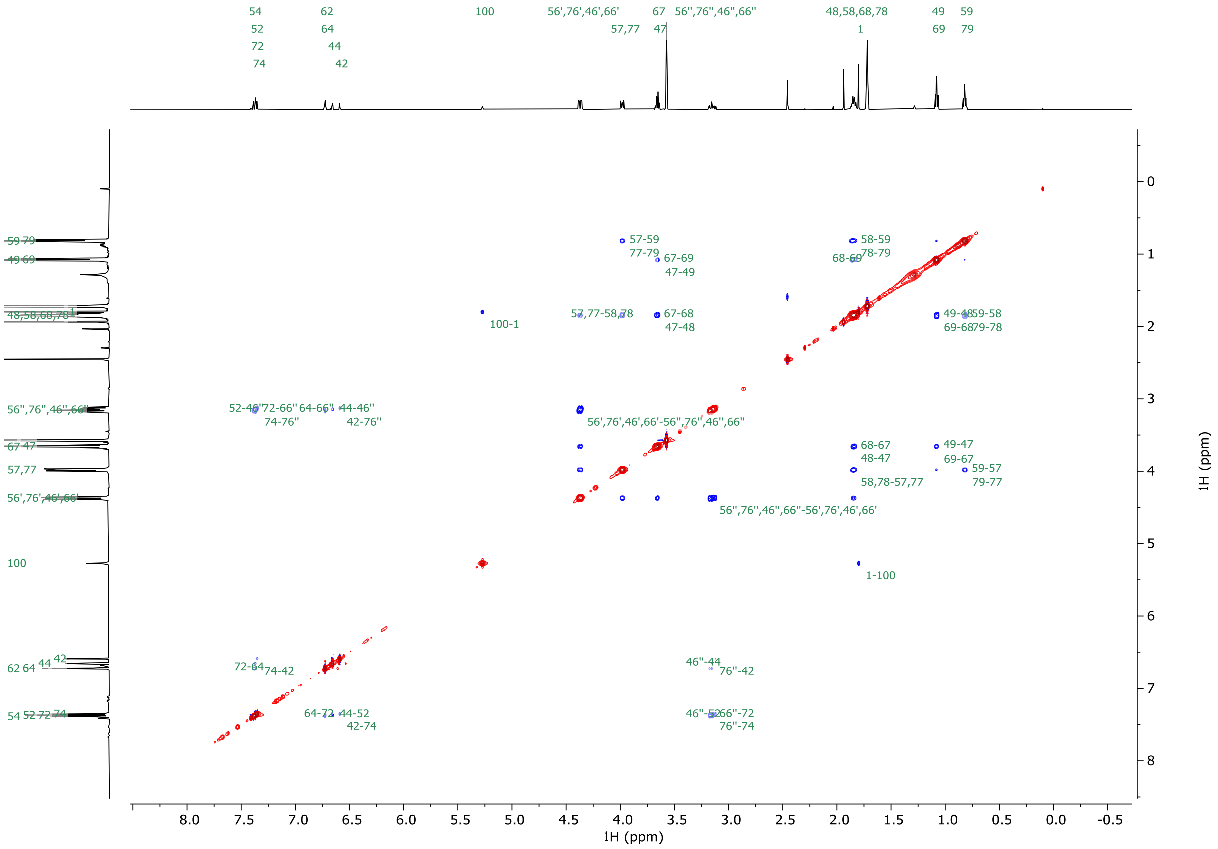

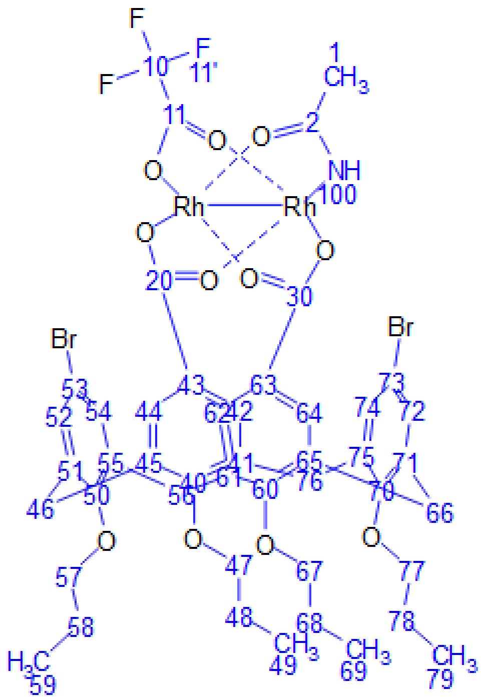

The NOE data suggests that all of –OPr groups in complex **7** are facing to the same side because X6' (X = 4, 5, 6, 7) shows cross correlations to X7. In contrast, X6'' show only NOEs to the aromatic signals X2 and X4.

Complex S6: ^{1}H NMR (600 MHz, [D$_8$]-THF):

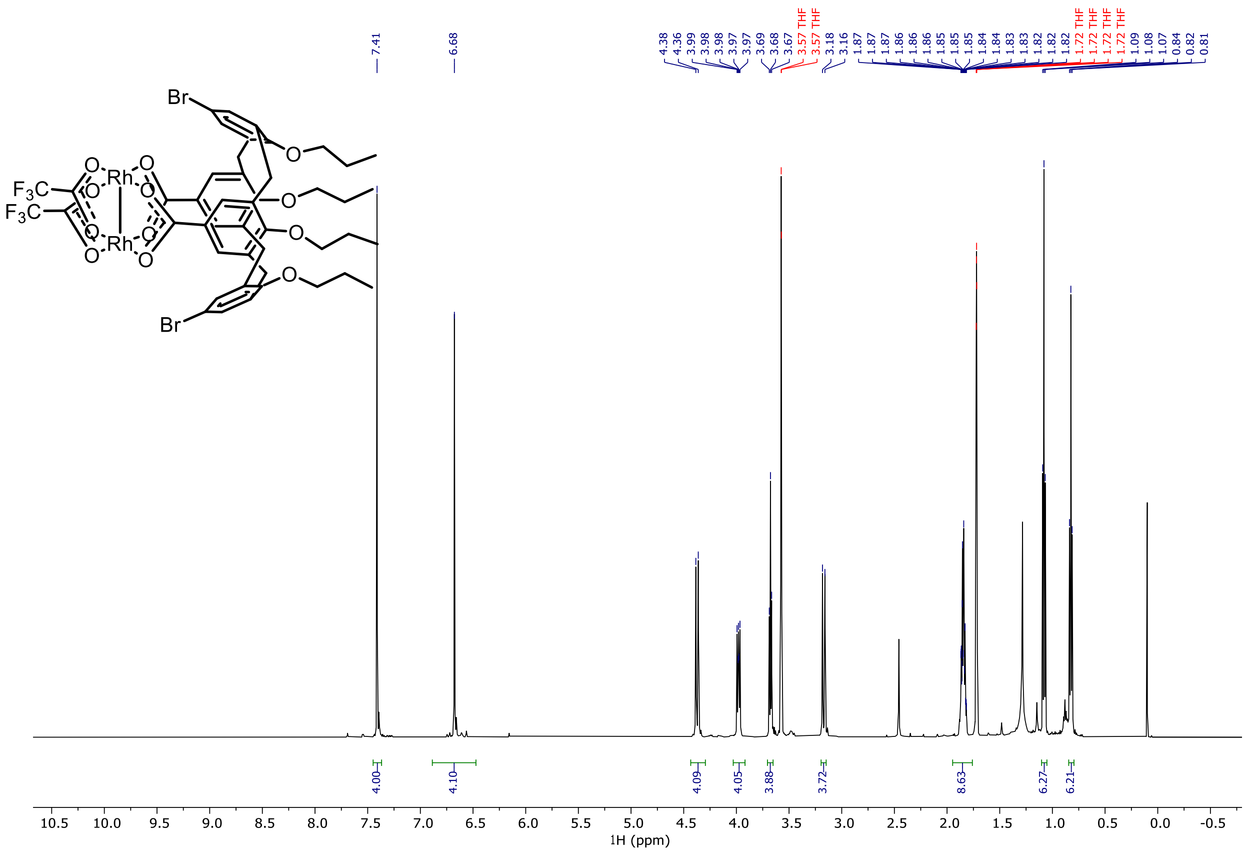

Complex S6: ^{13}C NMR (151 MHz, [D$_8$]-THF):

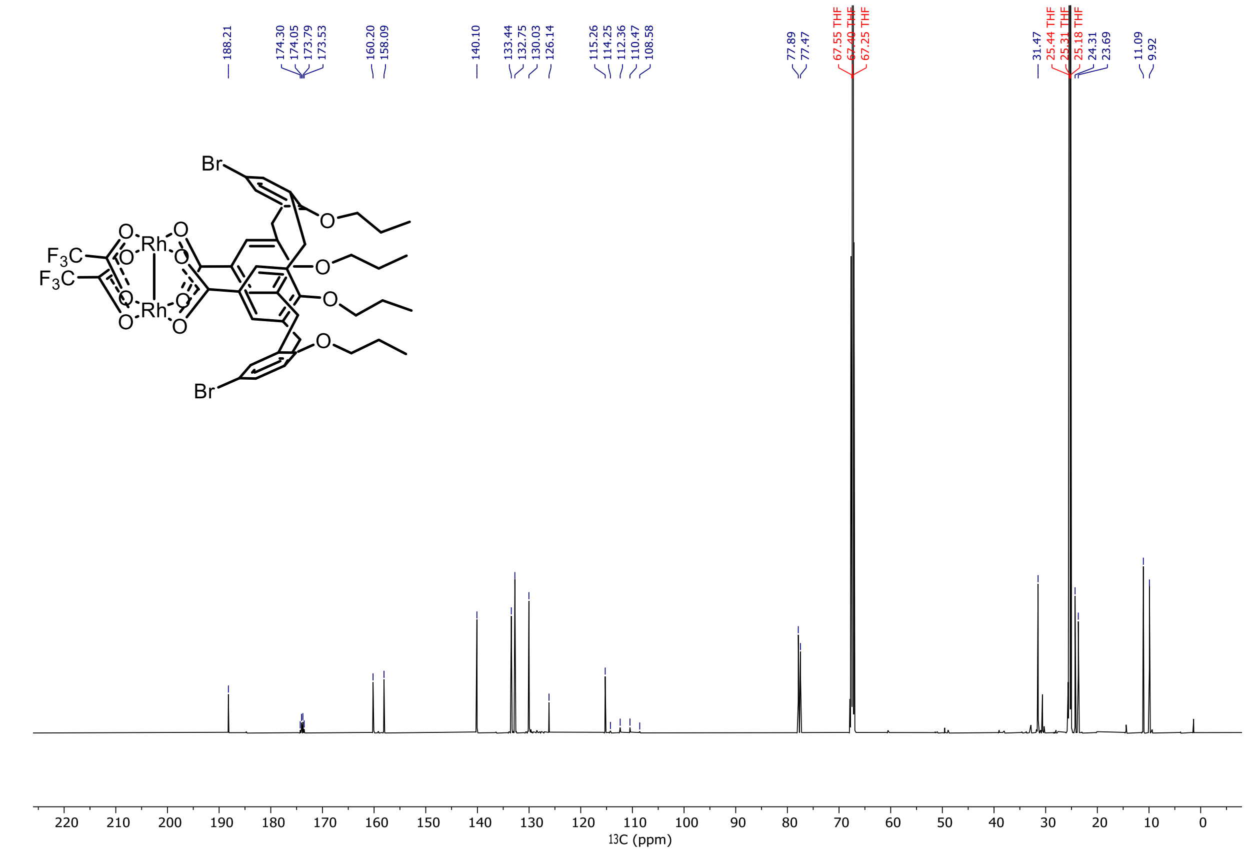

Complex S6: ^{19}F NMR (586 MHz, [D$_8$]-THF):

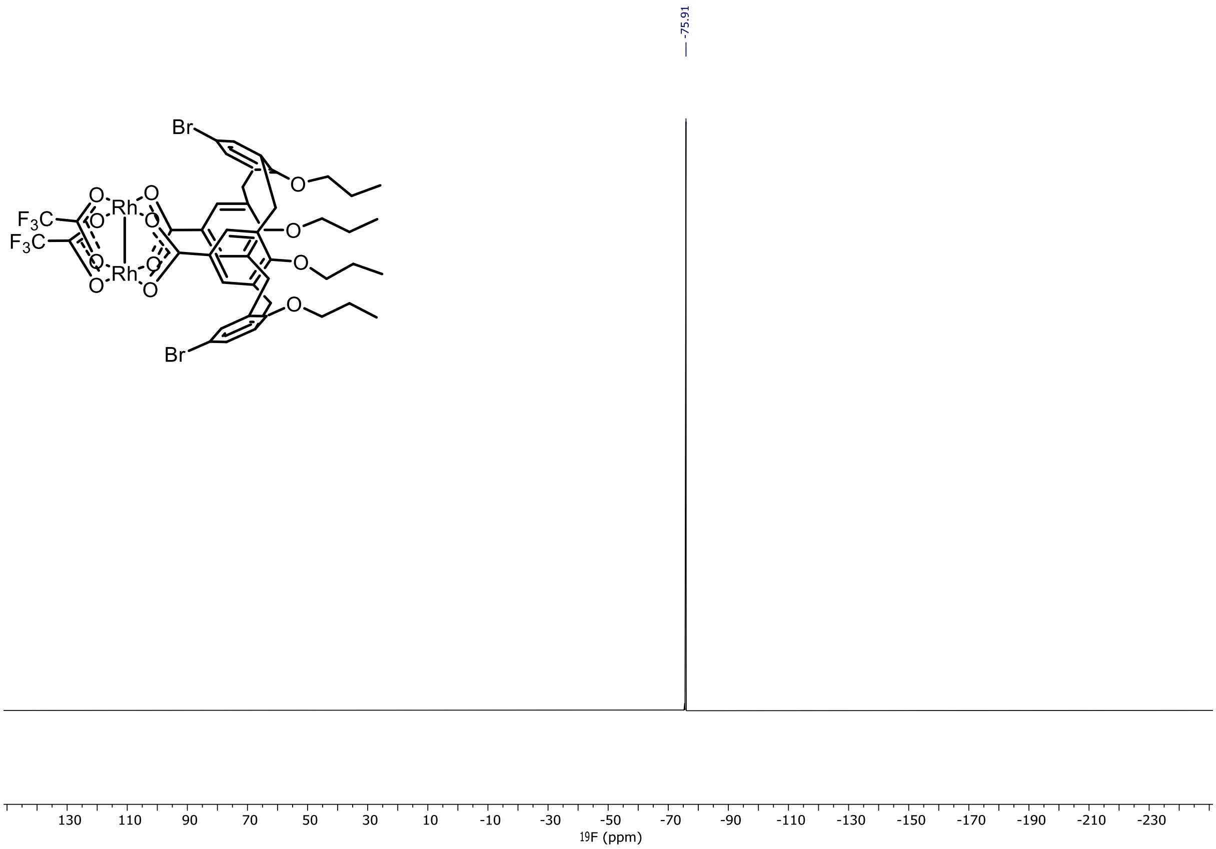

Complex S7: ^{1}H NMR (600 MHz, [D$_8$]-THF):

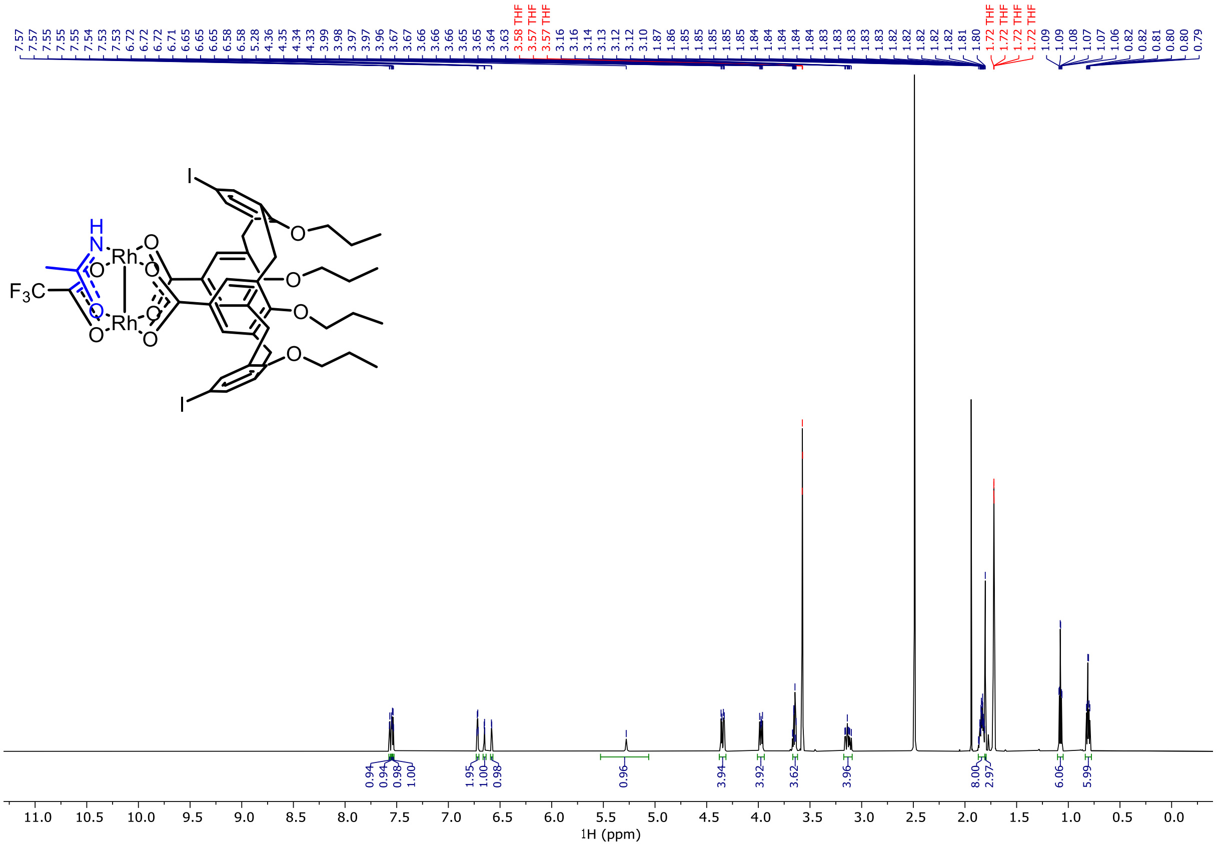

Complex S7: ^{13}C NMR (151 MHz, [D$_8$]-THF):

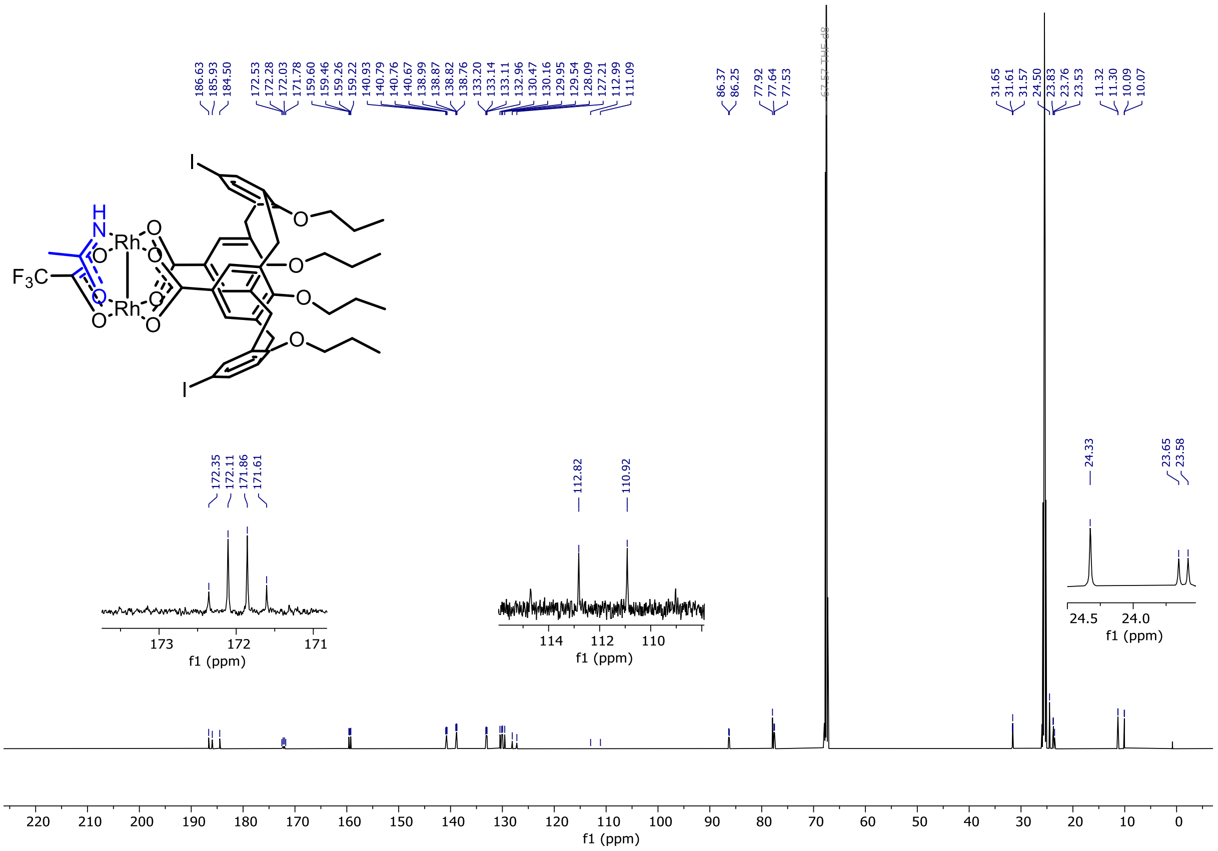

Complex S7: ^{19}F NMR (282 MHz, [D$_8$]-THF):

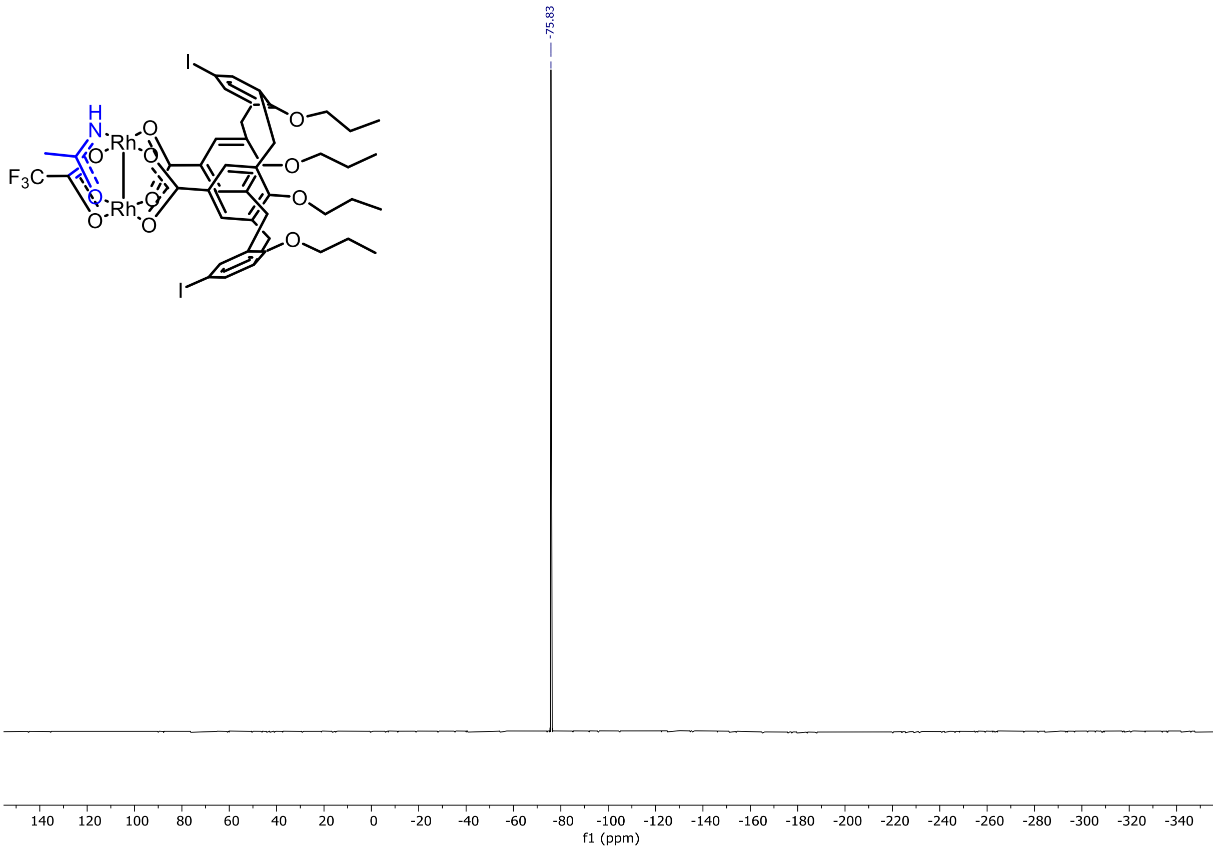

Complex 8: ^{1}H NMR (600 MHz, [D$_8$]-THF):

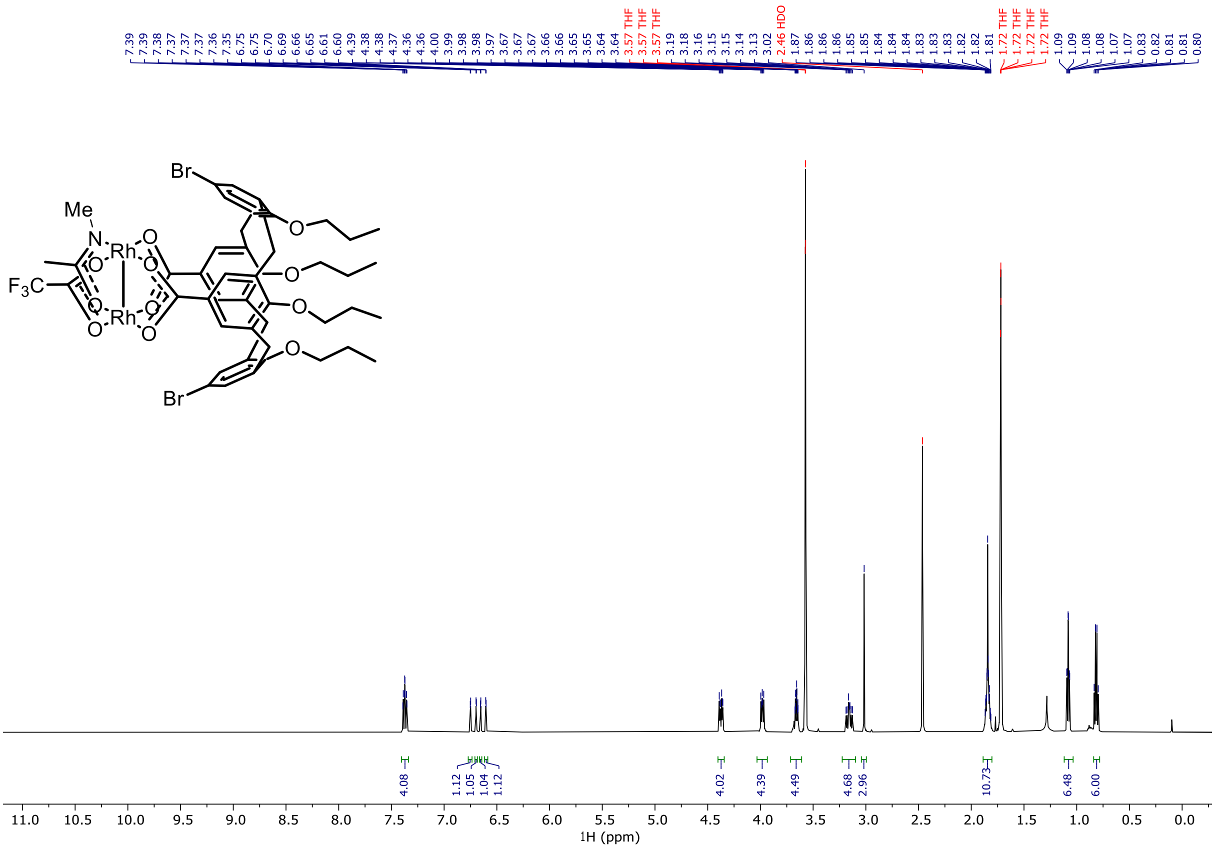

Complex 8: ^{13}C NMR (151 MHz, [D$_8$]-THF):

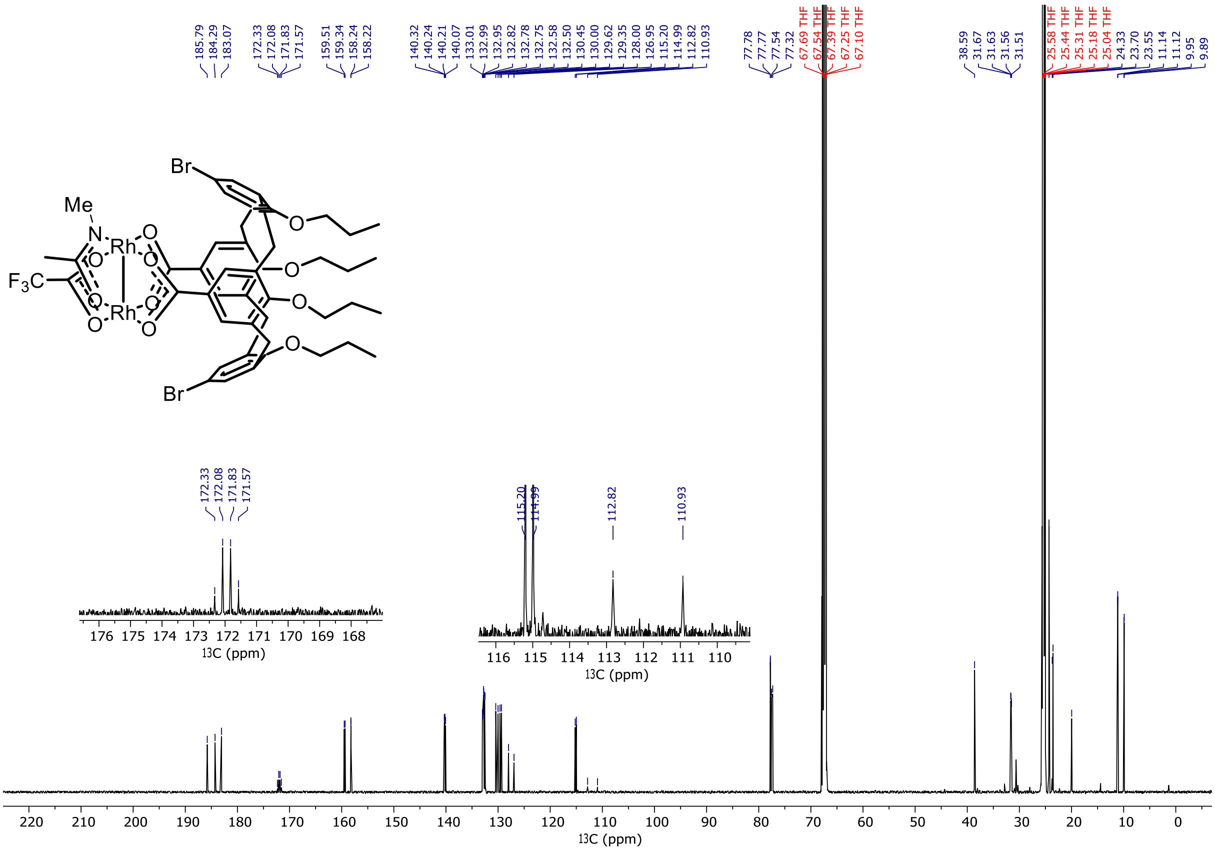

Complex 8: ^{19}F NMR (586 MHz, [D_8]-THF):

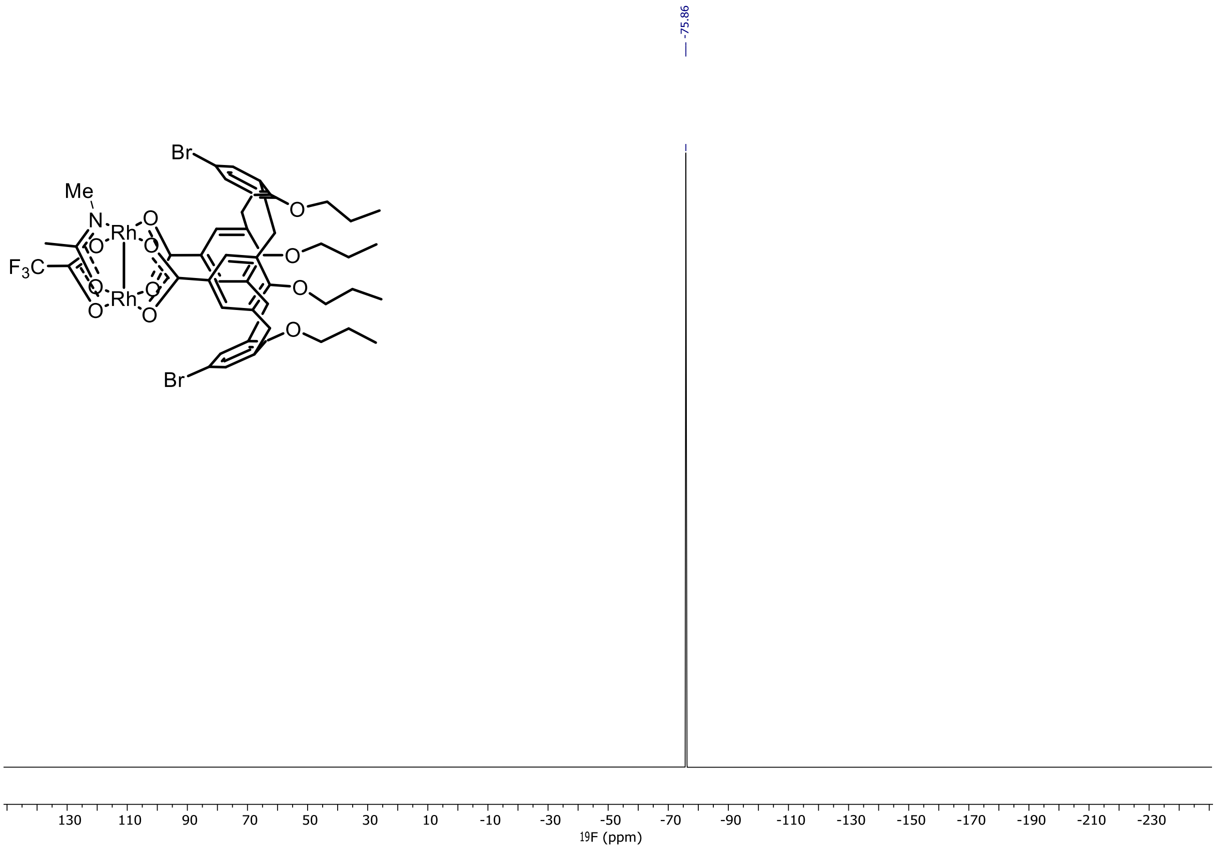

Complex S8: ^{1}H NMR (600 MHz, [D_8]-THF):

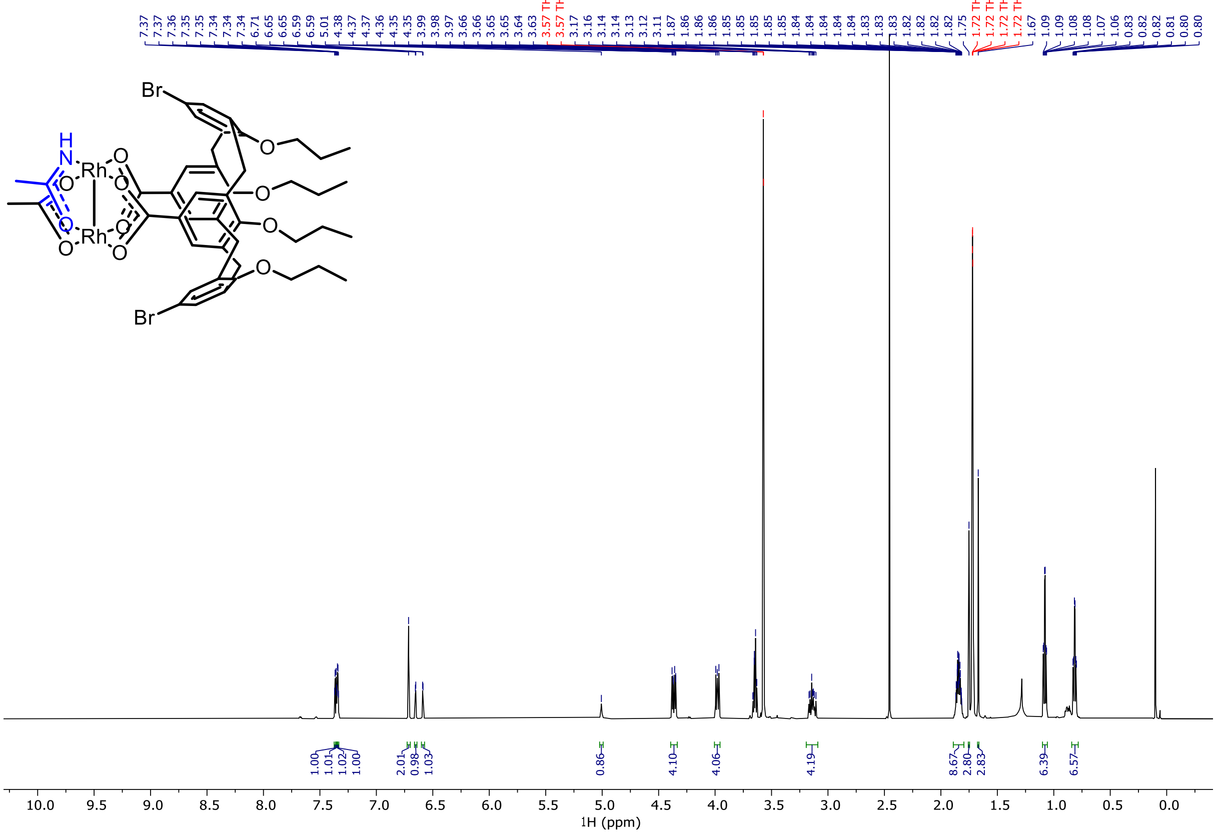

Complex S8: ^{13}C NMR (151 MHz, [D_8]-THF):

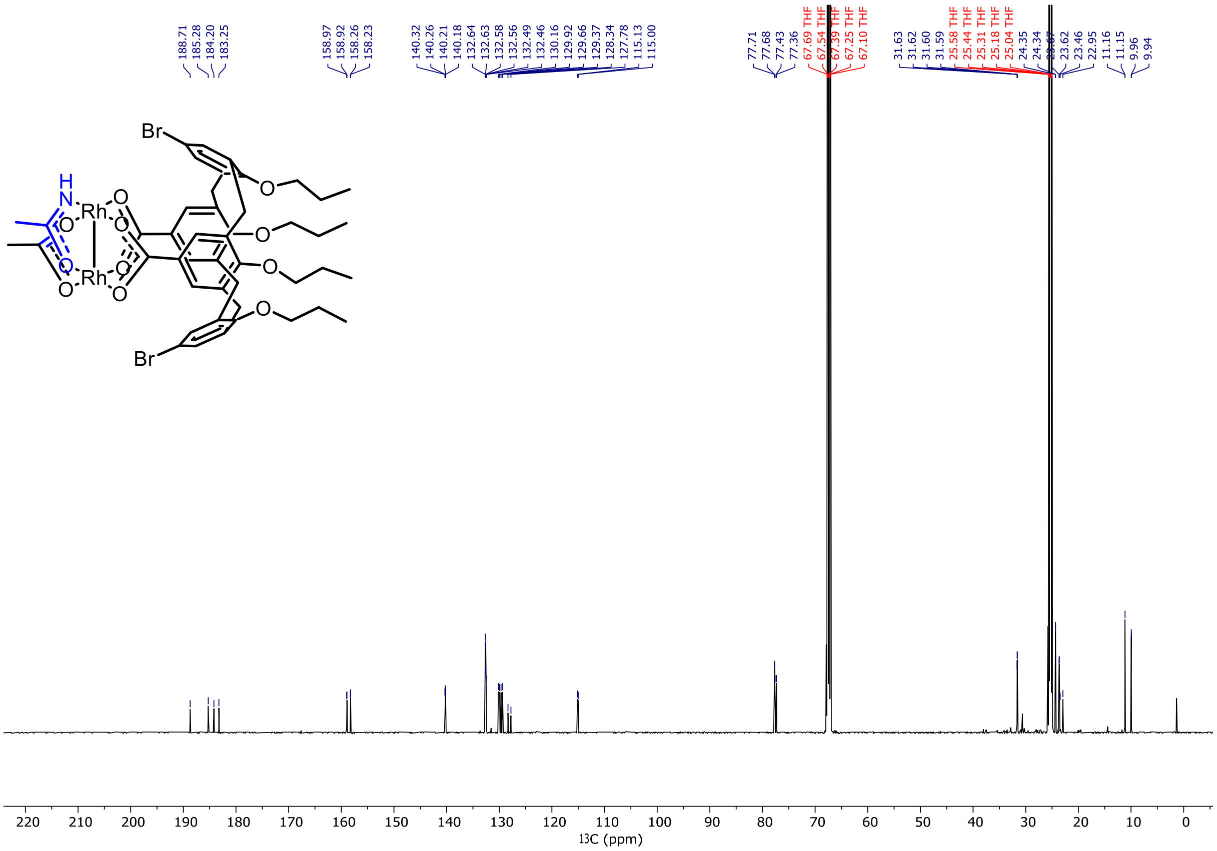

Compound 3a: ^{1}H NMR (400 MHz, $CDCl_3$):

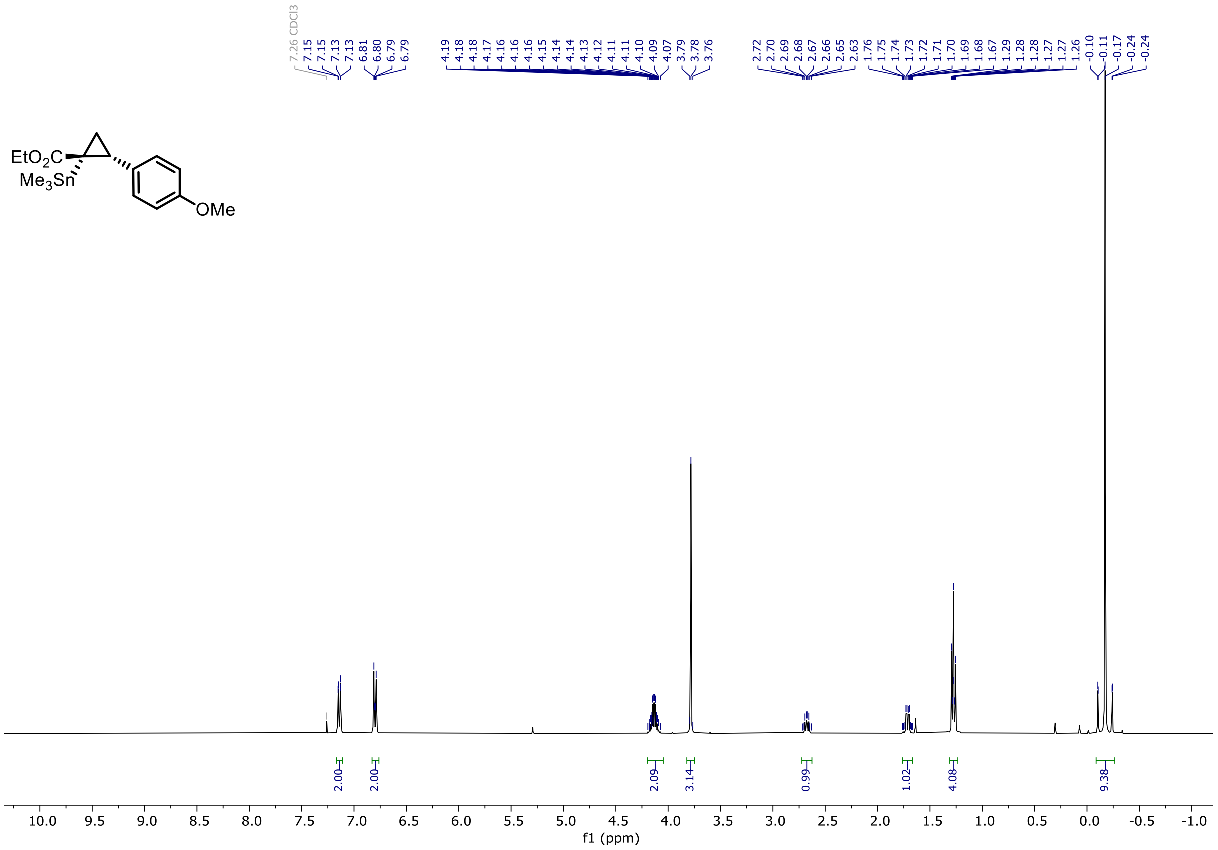

Compound 3a: ^{13}C NMR (101 MHz, $CDCl_3$):

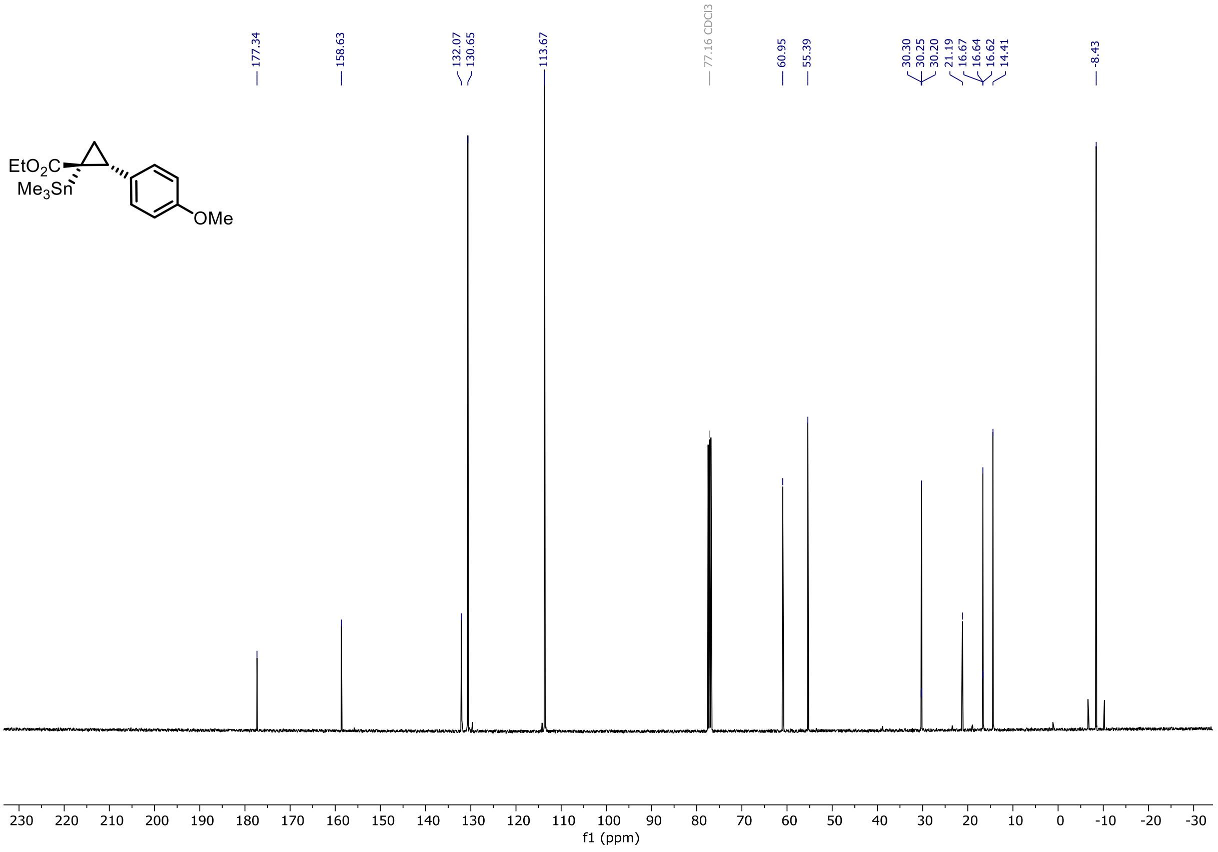

Compound 3a: ^{119}Sn NMR (149 MHz, $CDCl_3$):

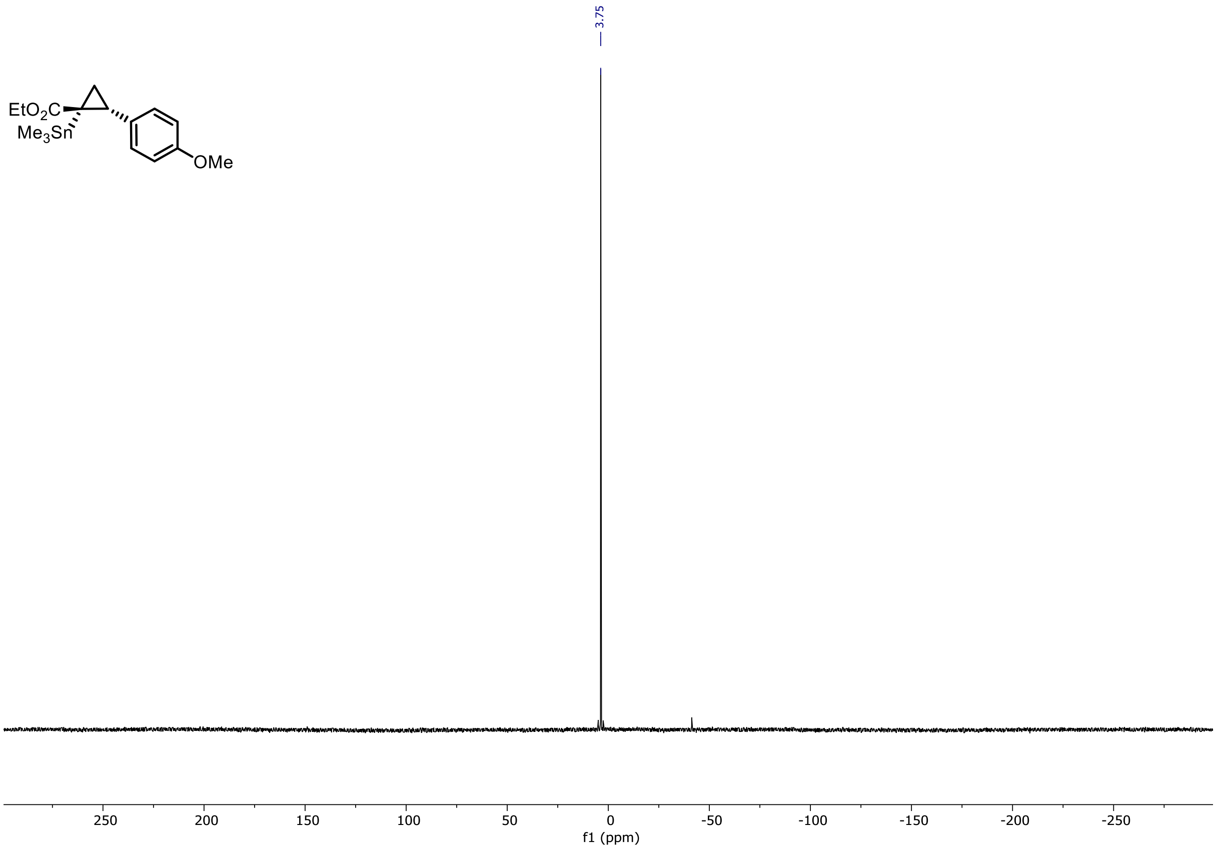

Compound 3b: ^{1}H NMR (600 MHz, $CDCl_3$):

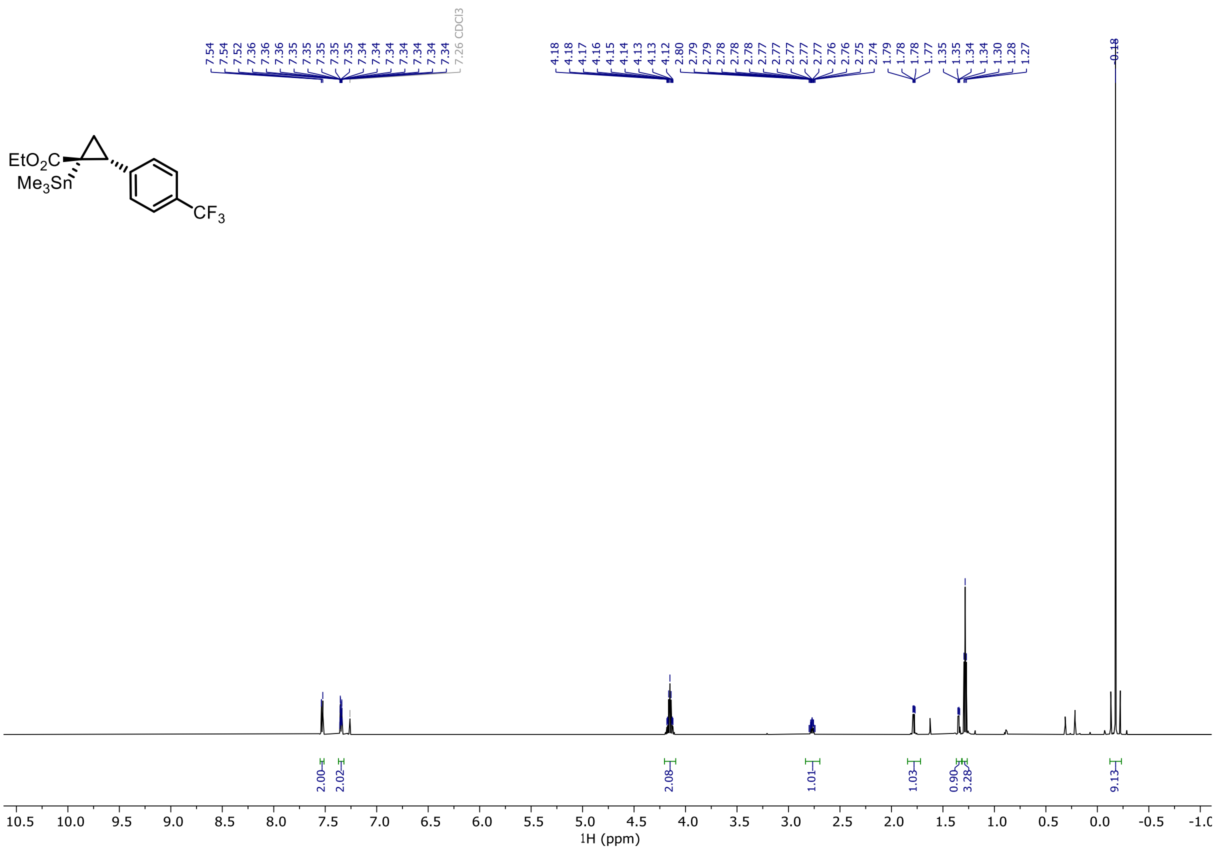

Compound 3b: ^{13}C NMR (151 MHz, $CDCl_3$):

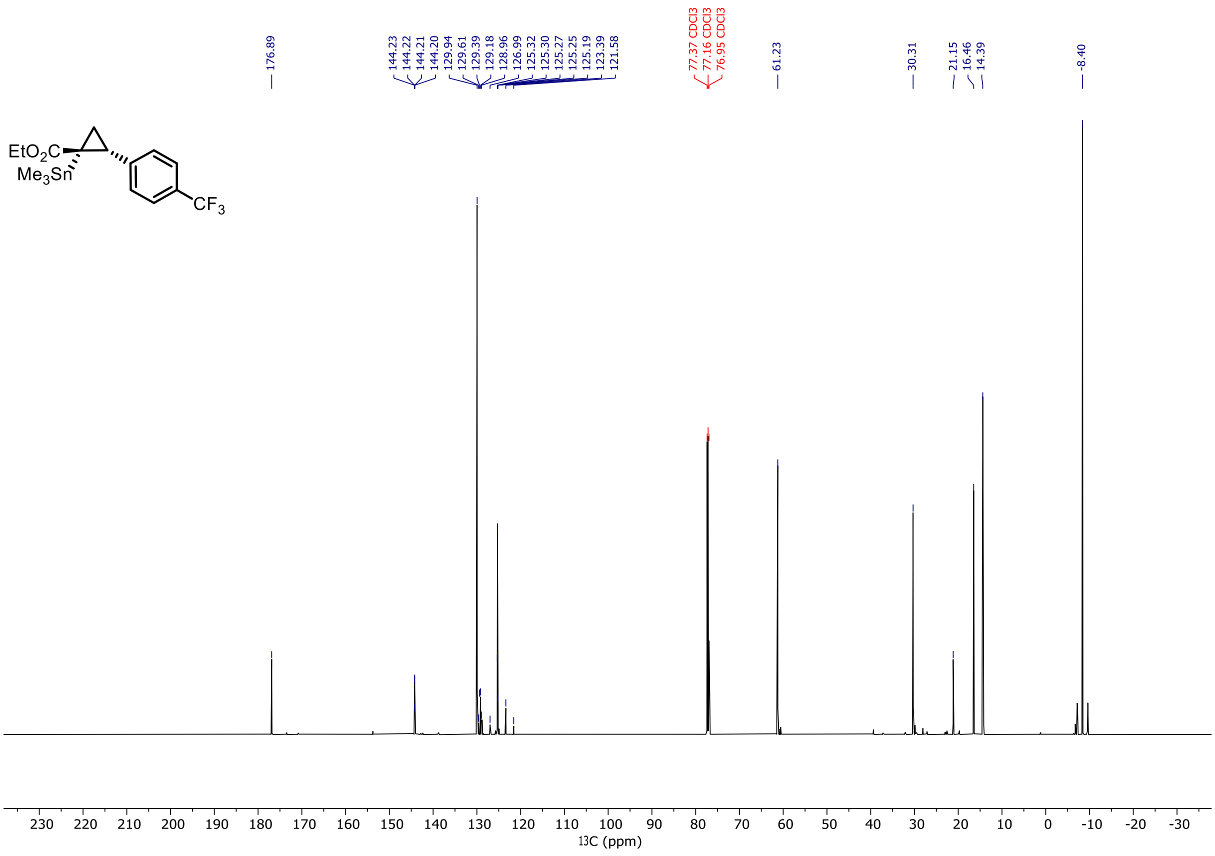

Compound 3b: ^{19}F NMR (565 MHz, $CDCl_3$):

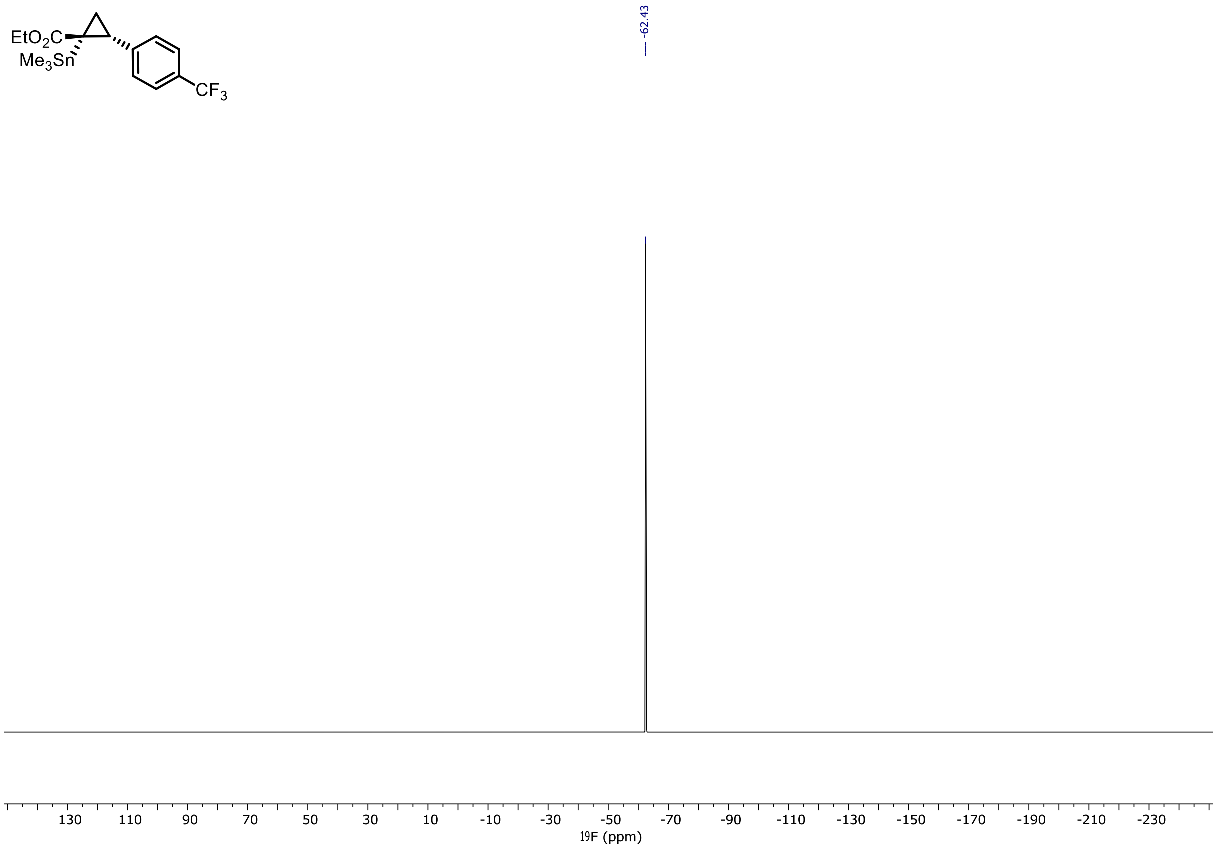

Compound 3b: ^{119}Sn NMR (224 MHz, $CDCl_3$):

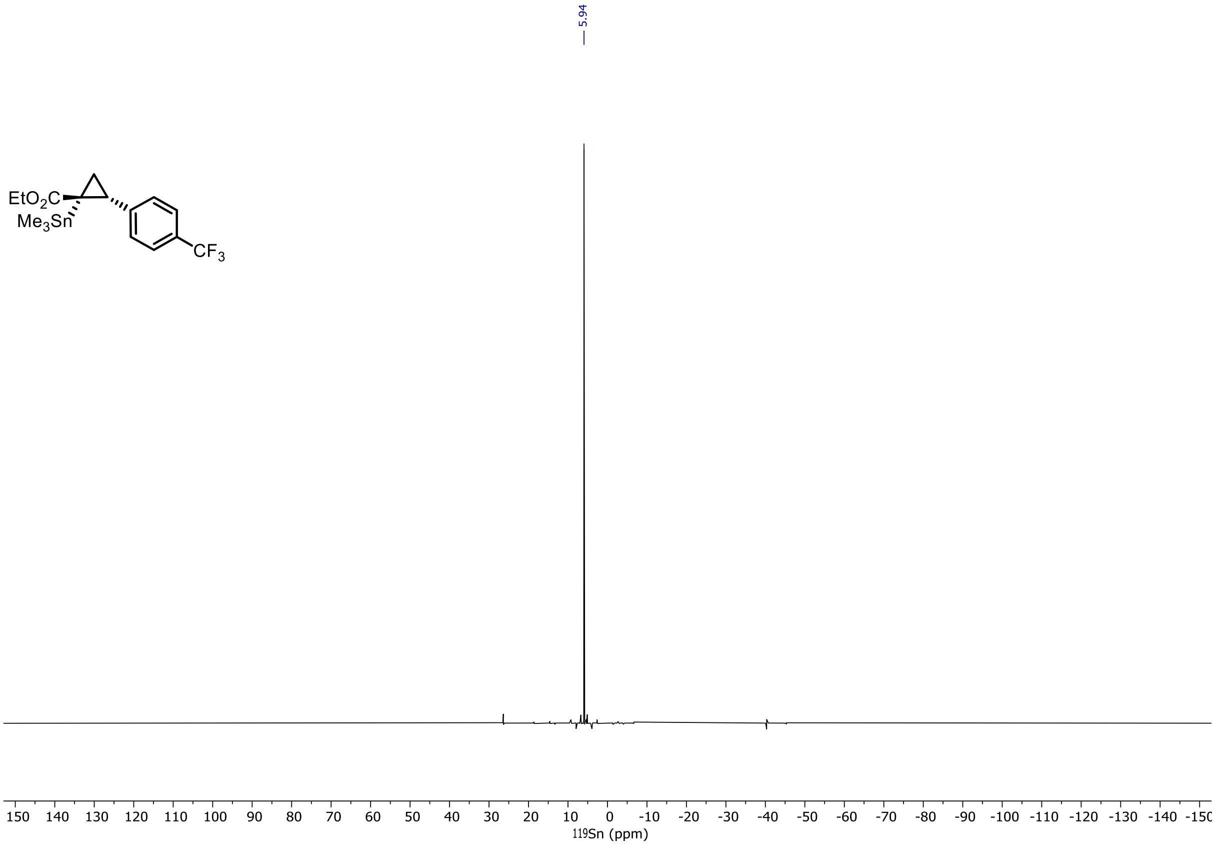

Compound 3b: ^{1}H-^{1}H-NOESY ($CDCl_3$):

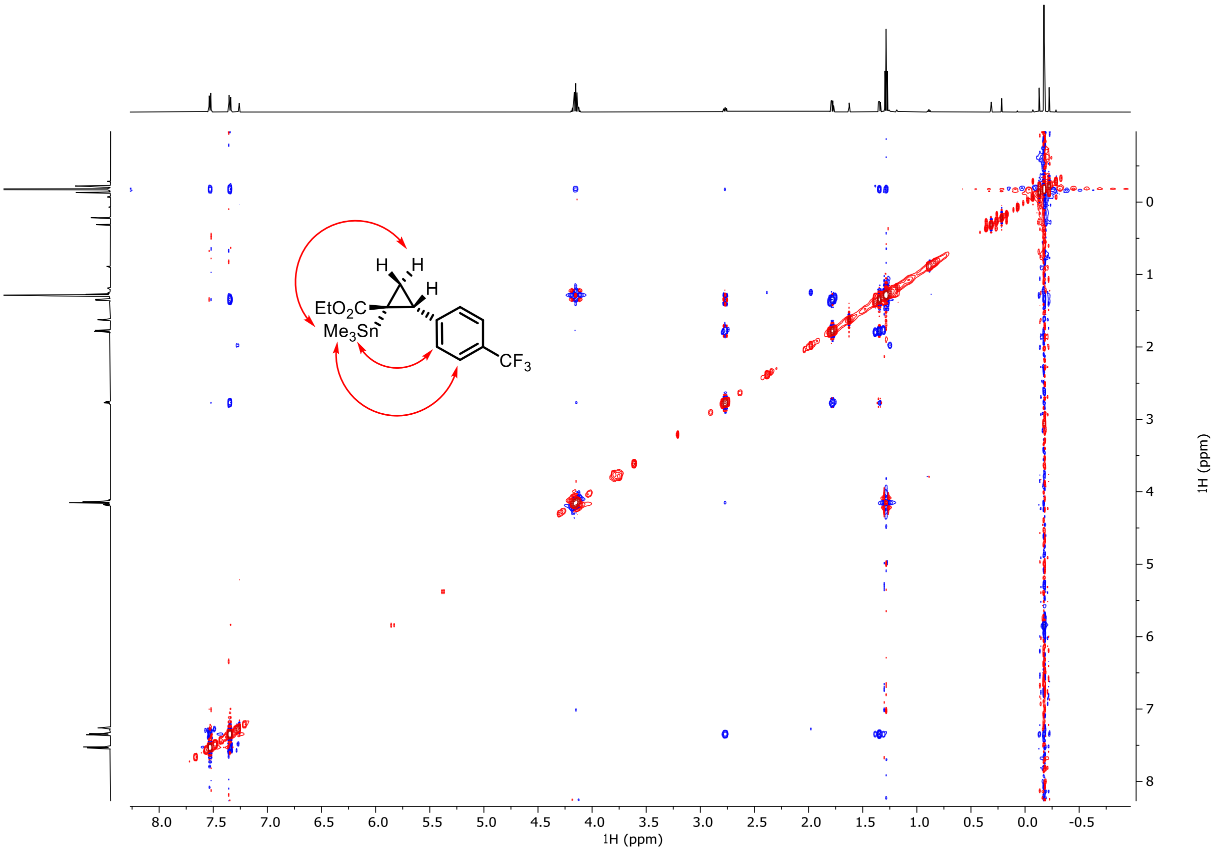

Compound 3c: ^{1}H NMR (400 MHz, $CDCl_3$) (*cis/trans* mixture):

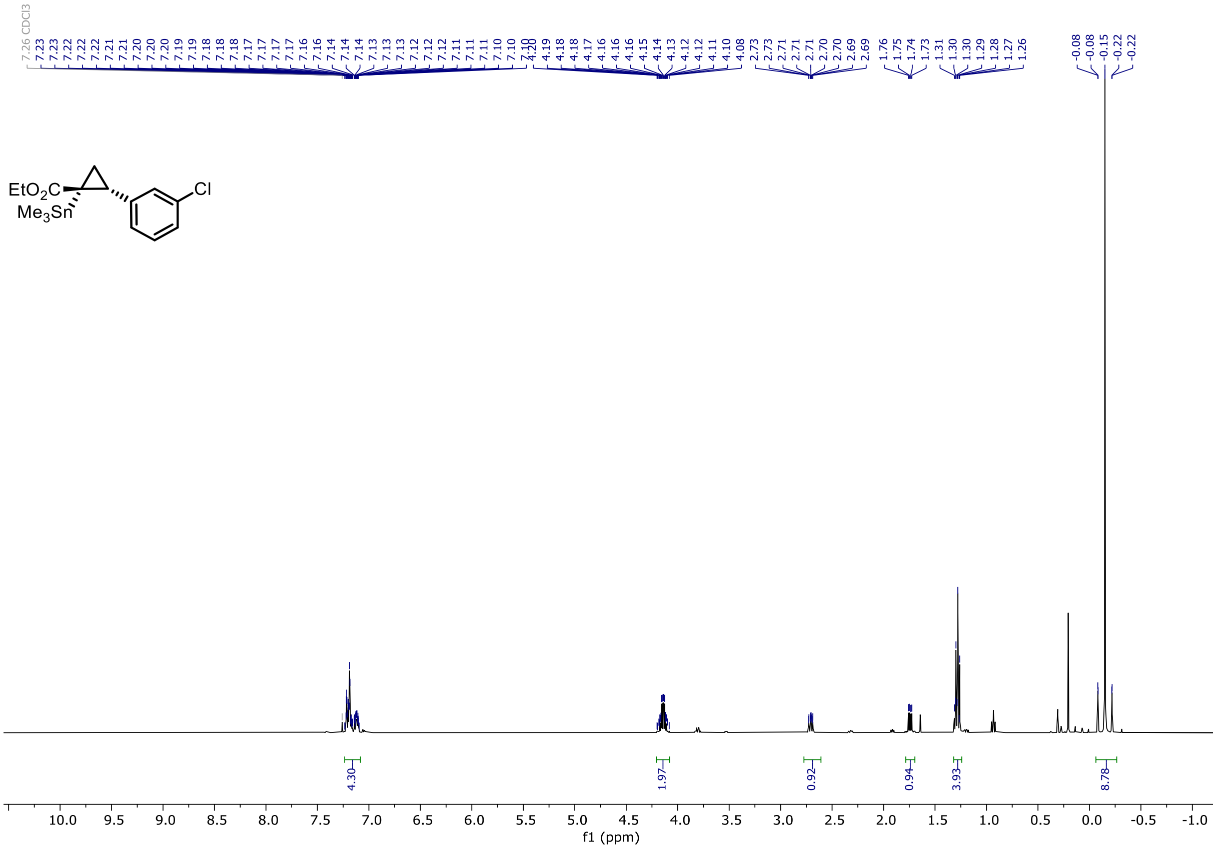

Compound 3c: ^{13}C NMR (101 MHz, $CDCl_3$) (*cis/trans* mixture):

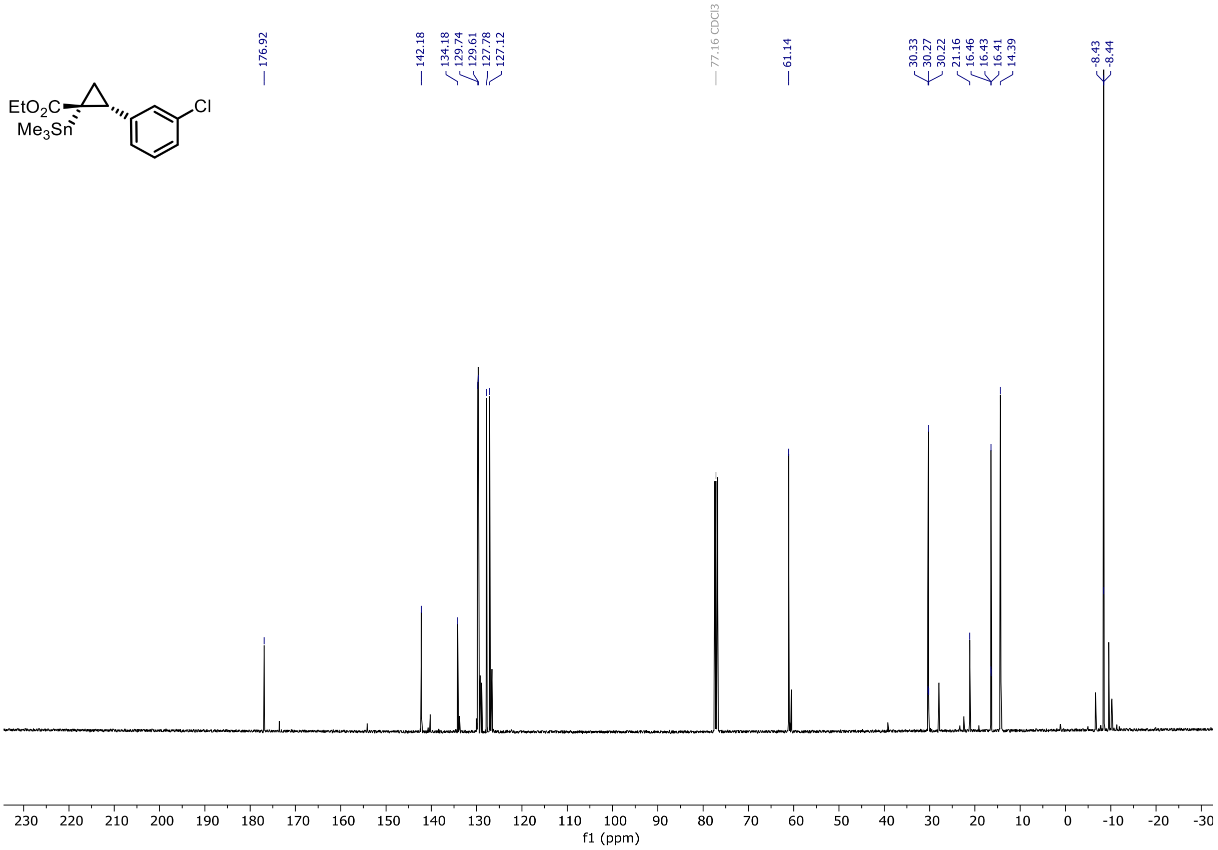

Compound 3c: ^{119}Sn NMR (149 MHz, $CDCl_3$) (*cis/trans* mixture):

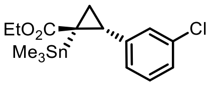

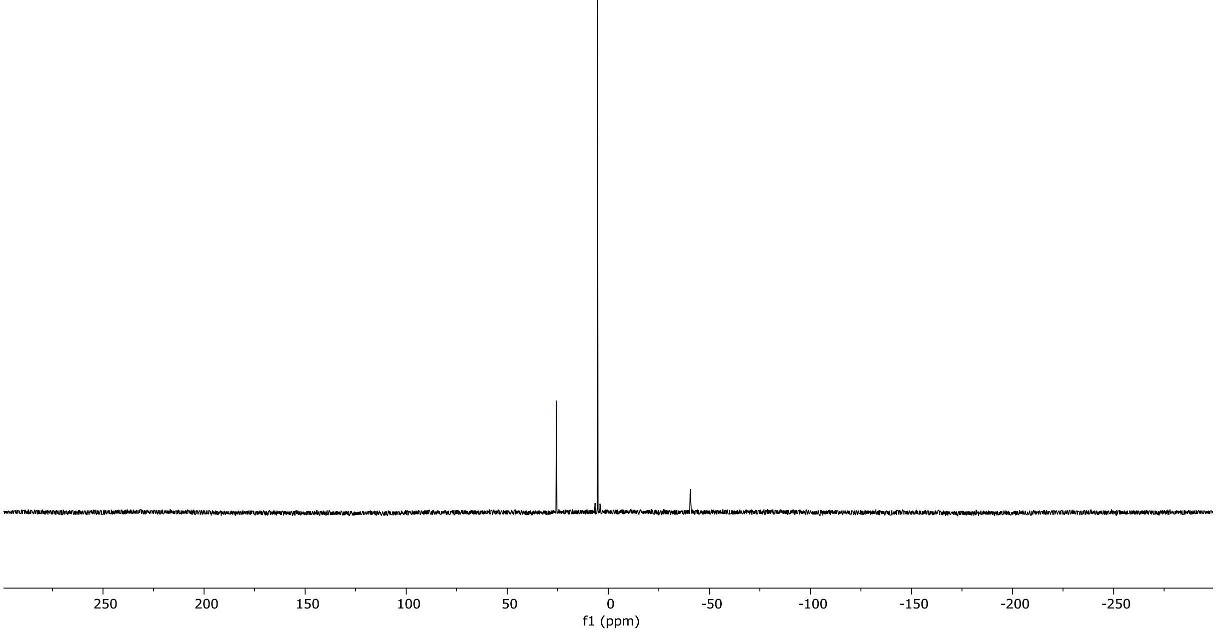

Compound 3d: ^{1}H NMR (400 MHz, $CDCl_3$):

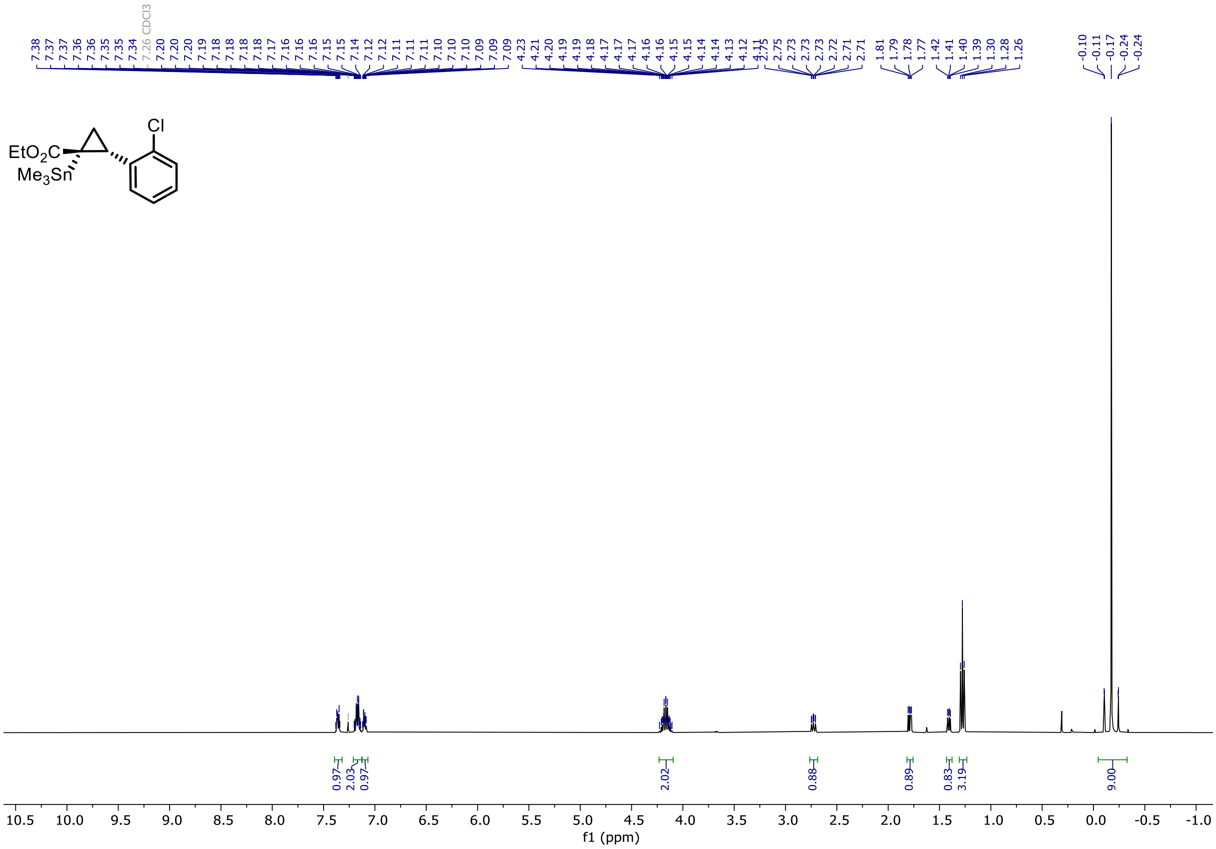

Compound 3d: ^{13}C NMR (101 MHz, $CDCl_3$):

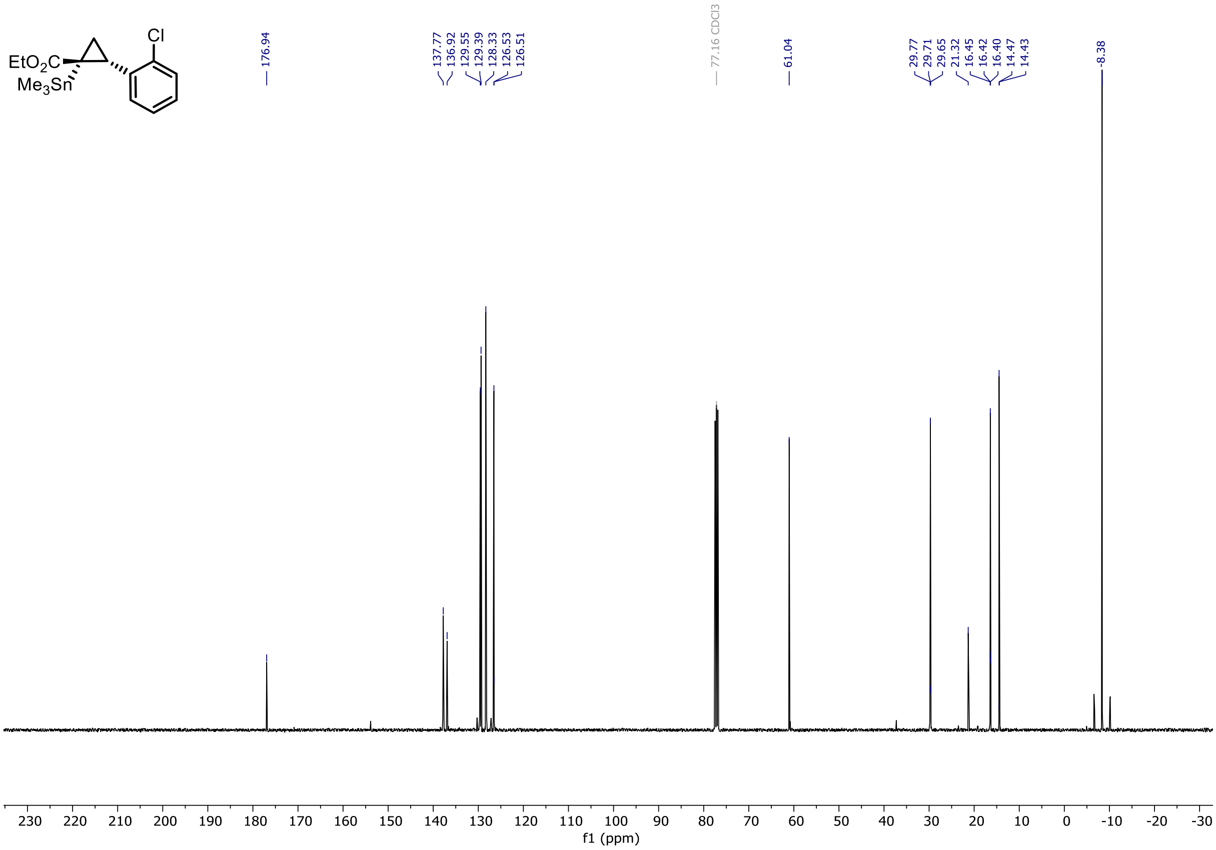

Compound 3d: ^{119}Sn NMR (149 MHz, $CDCl_3$):

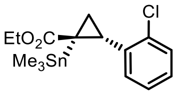

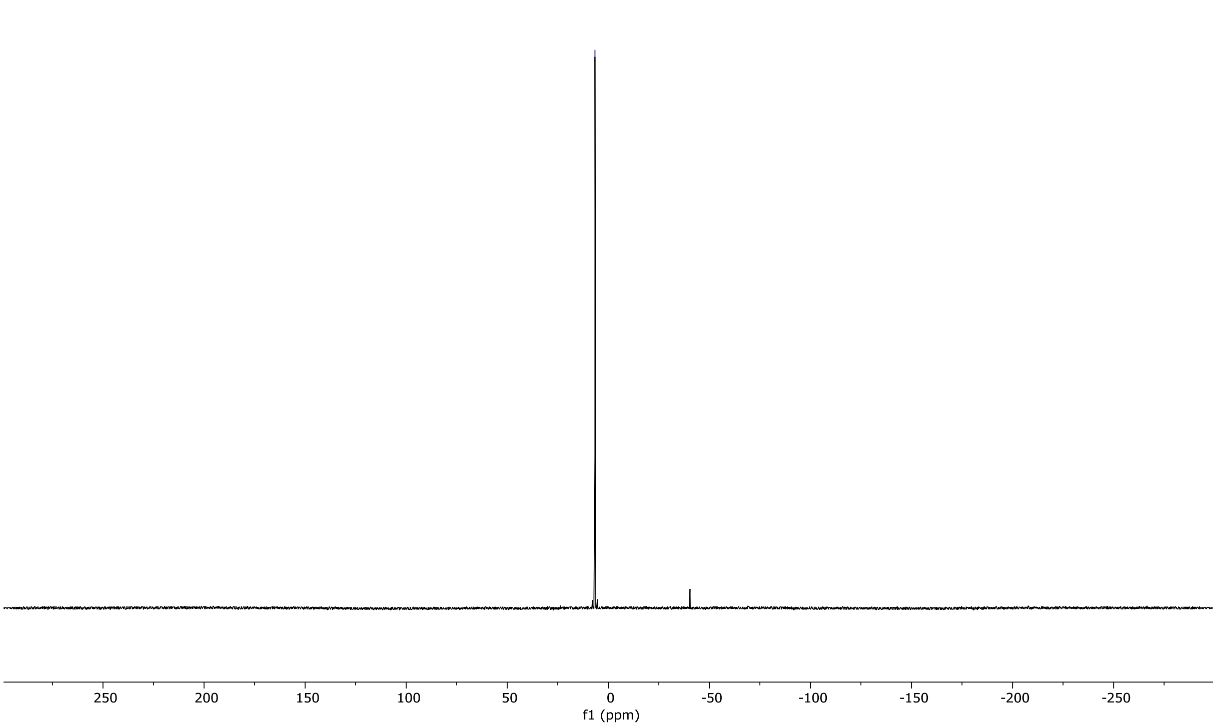

Compound 3e: ^{1}H NMR (400 MHz, $CDCl_3$):

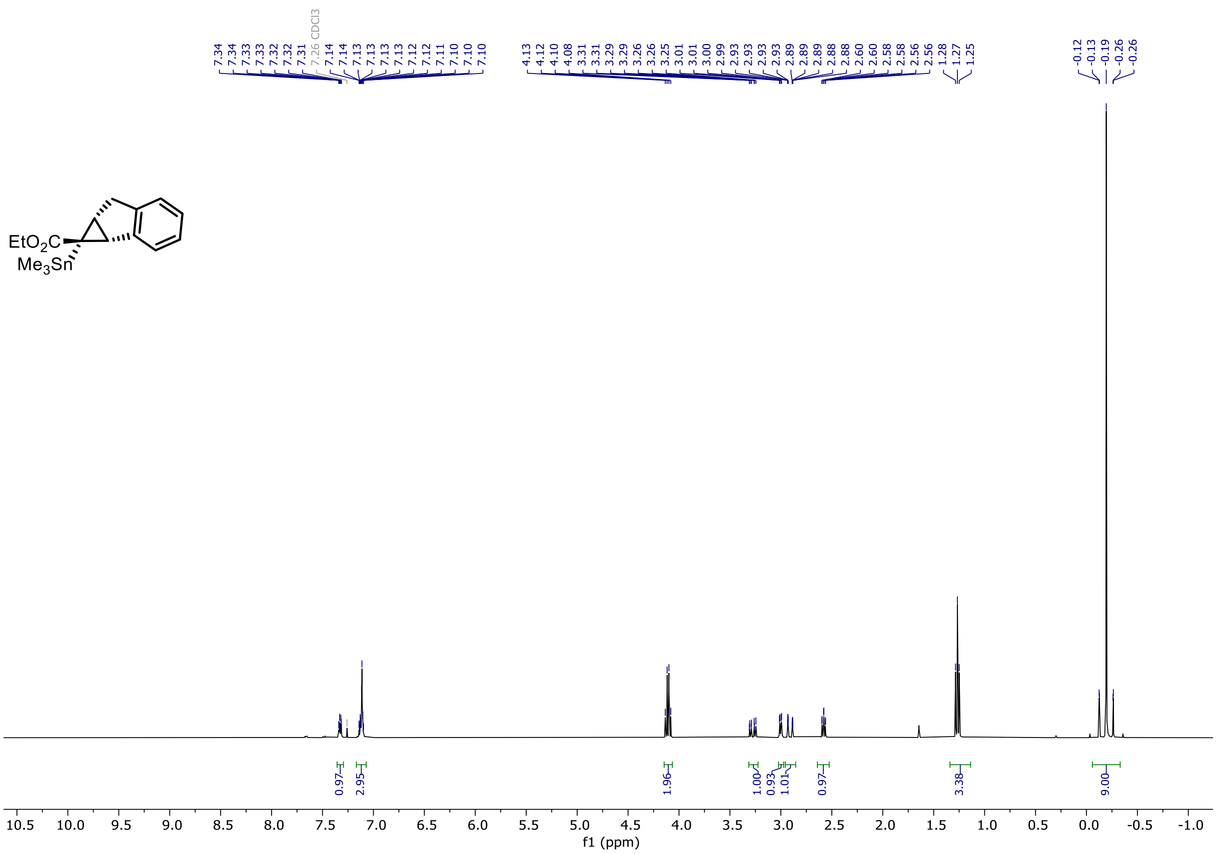

Compound 3e: ^{13}C NMR (101 MHz, $CDCl_3$):

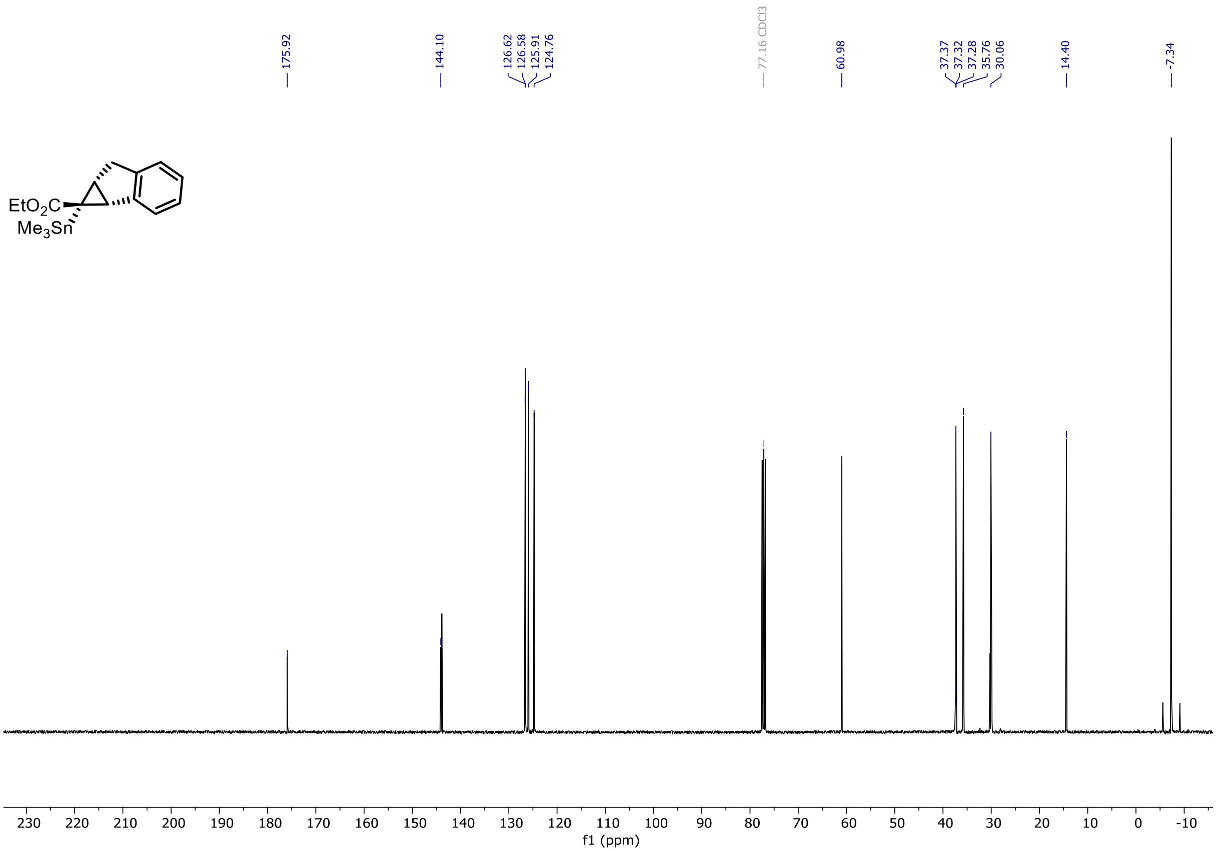

Compound 3e: ^{119}Sn NMR (149 MHz, $CDCl_3$):

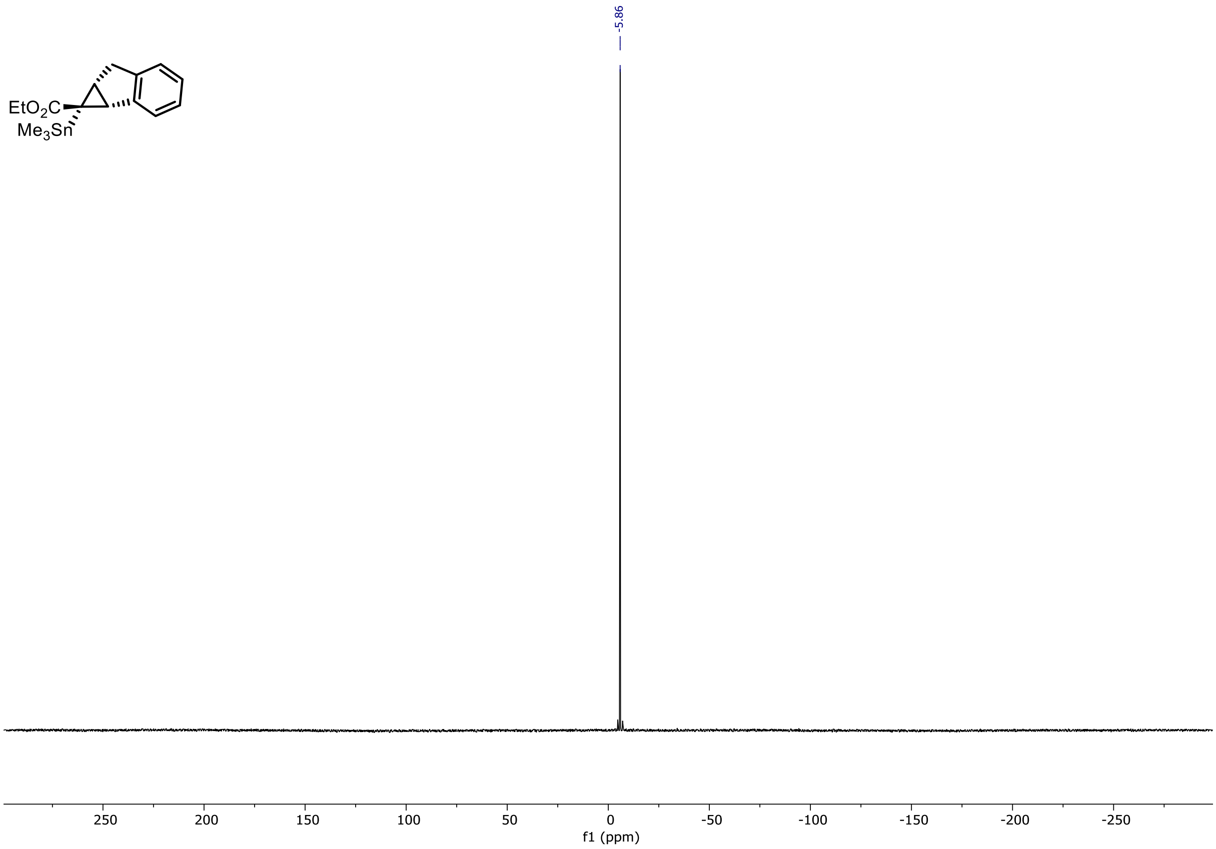

Compound 3f: ^{1}H NMR (400 MHz, $CDCl_3$) (*cis/trans* mixture):

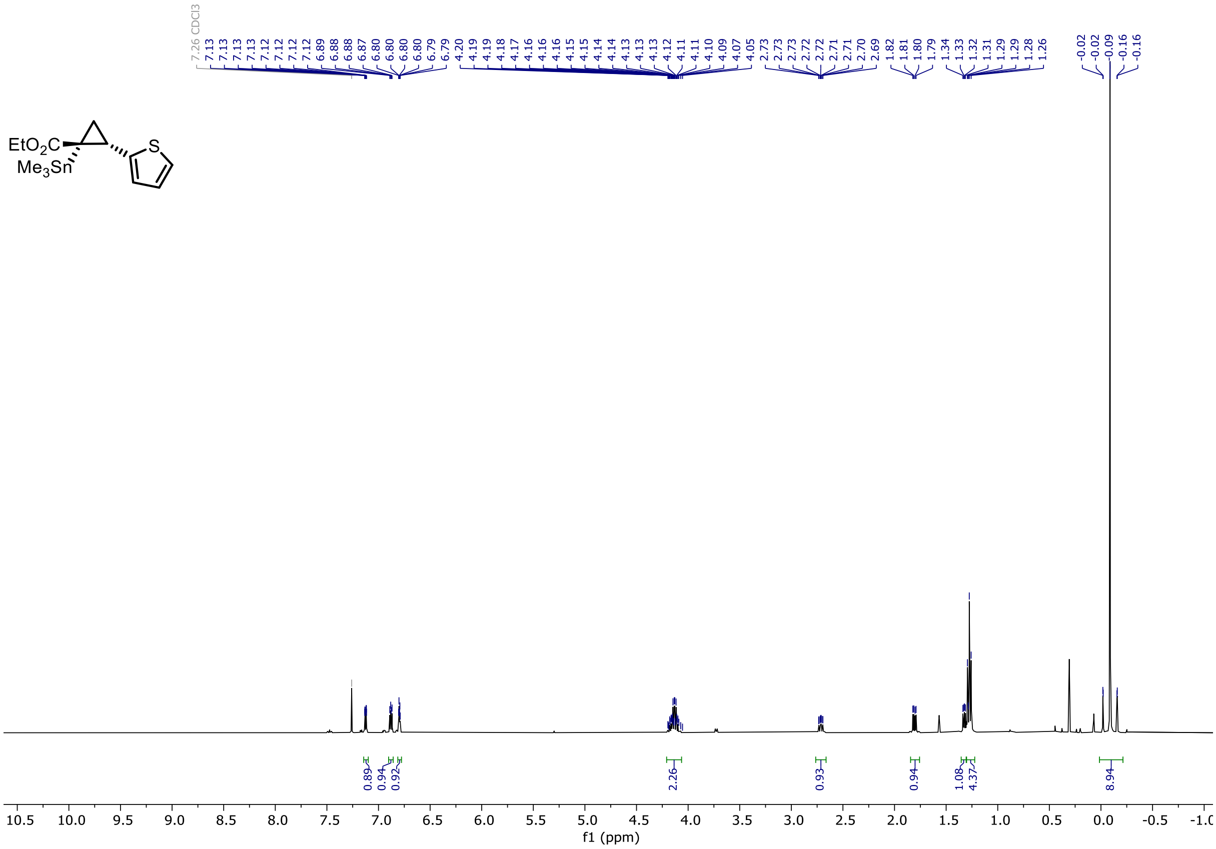

Compound 3f: ^{13}C NMR (101 MHz, $CDCl_3$) (*cis/trans* mixture):

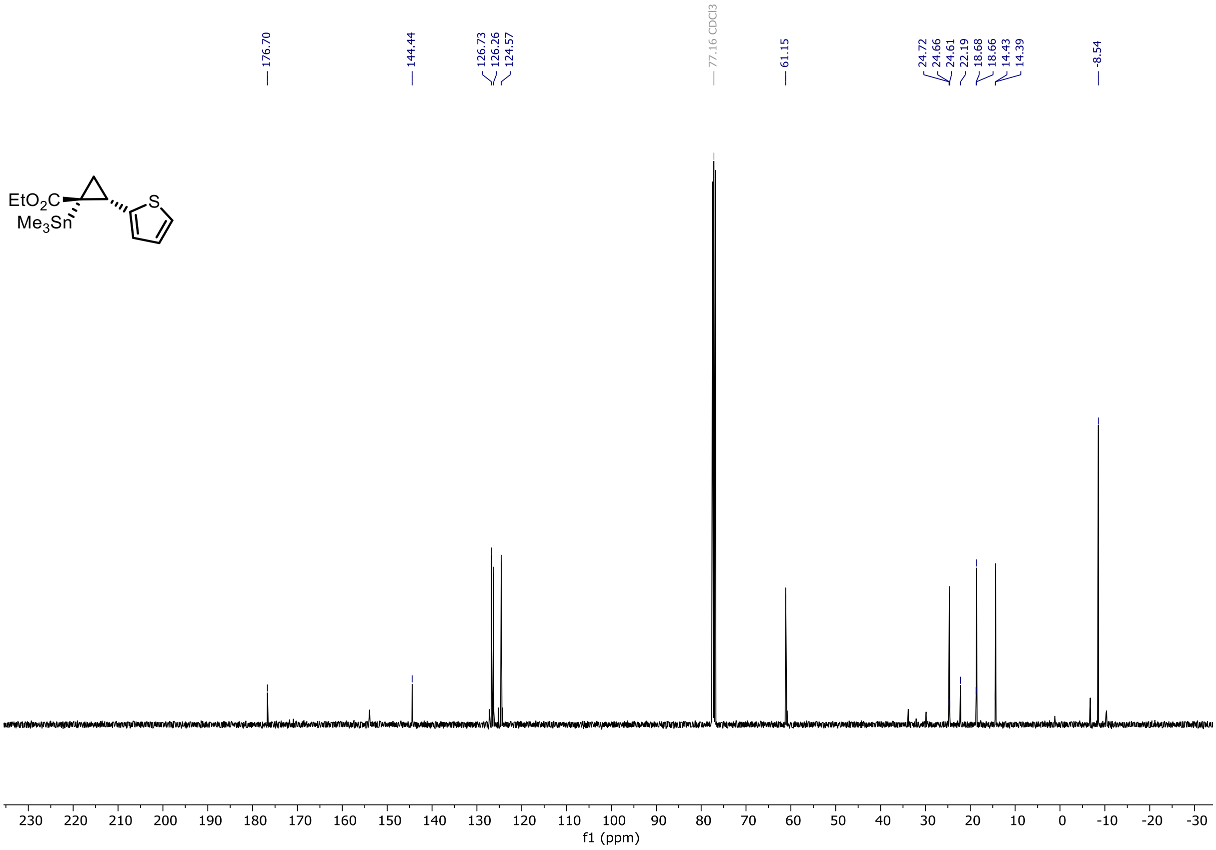

Compound 3f: ^{119}Sn NMR (149 MHz, $CDCl_3$) (*cis/trans* mixture):

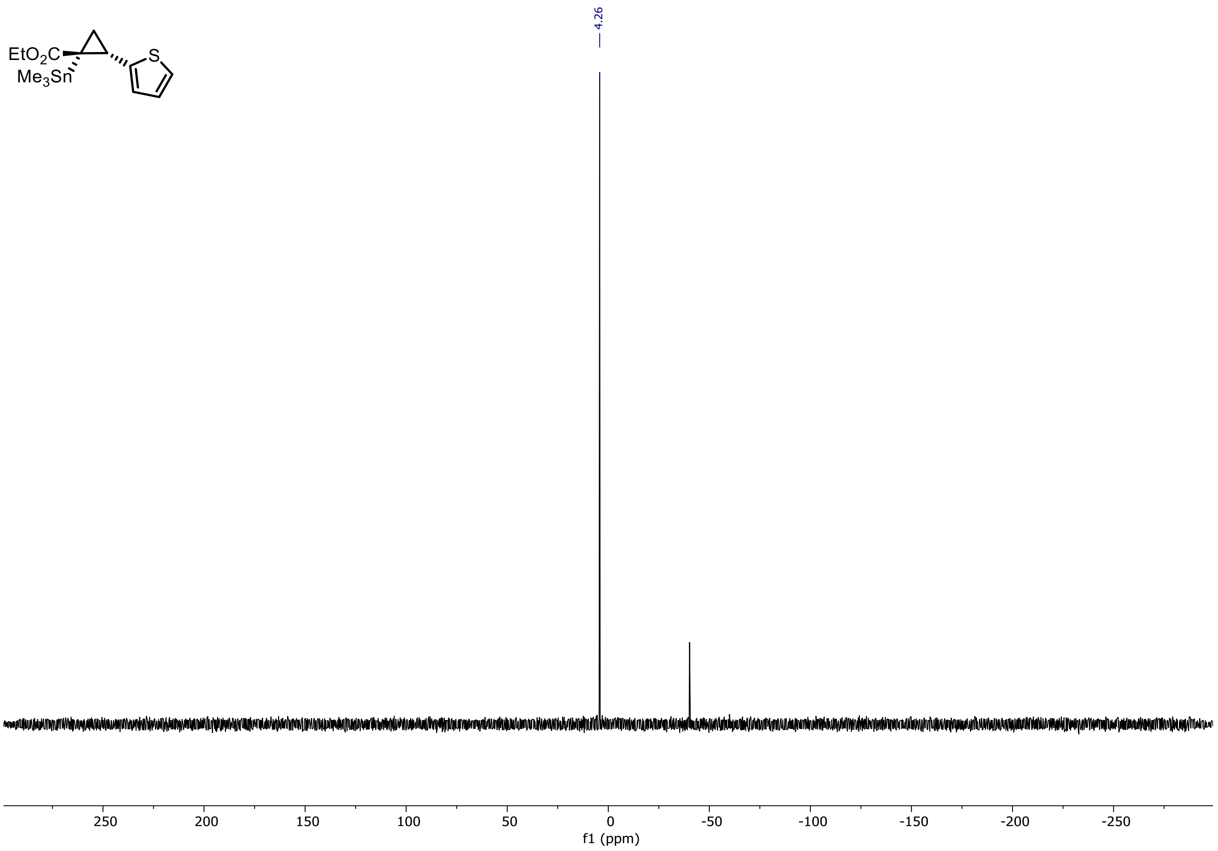

Compound 3g: ^{1}H NMR (400 MHz, $CDCl_3$):

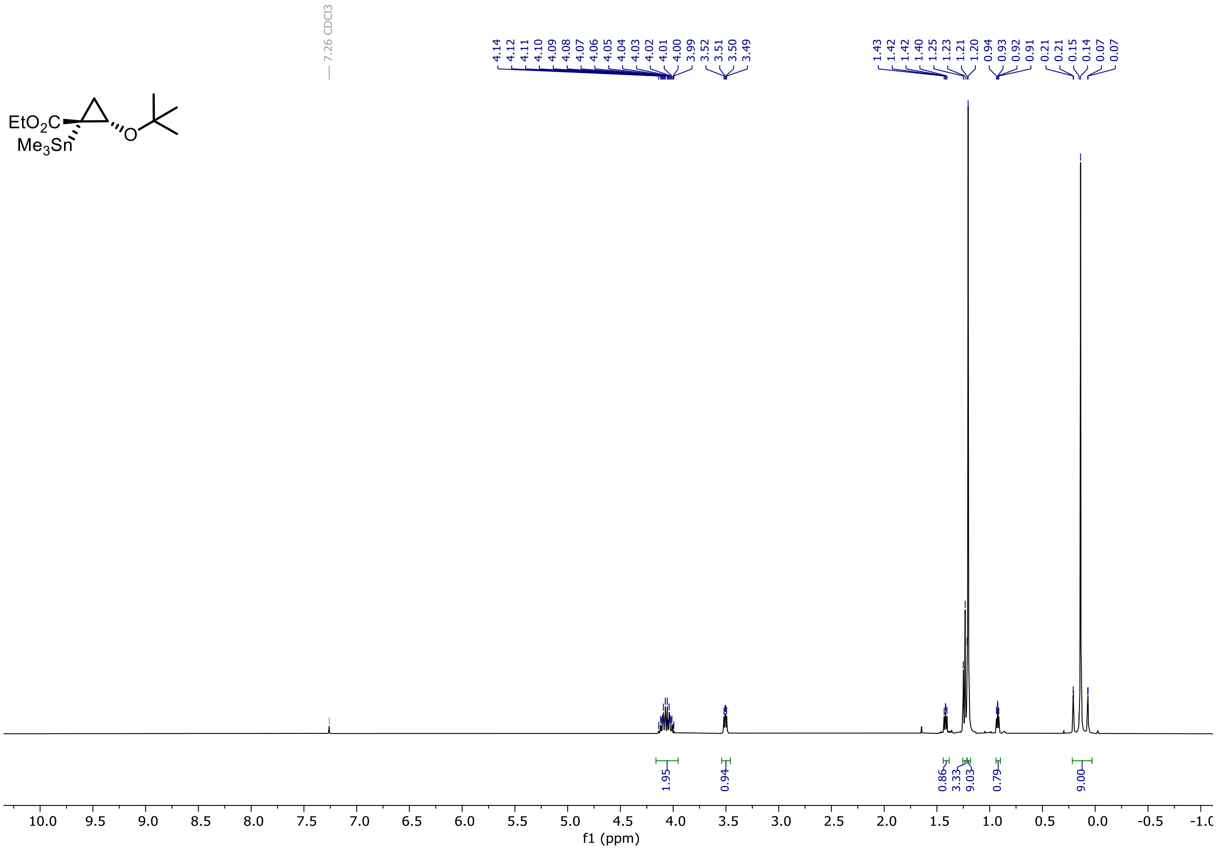

Compound 3g: ^{13}C NMR (101 MHz, $CDCl_3$):

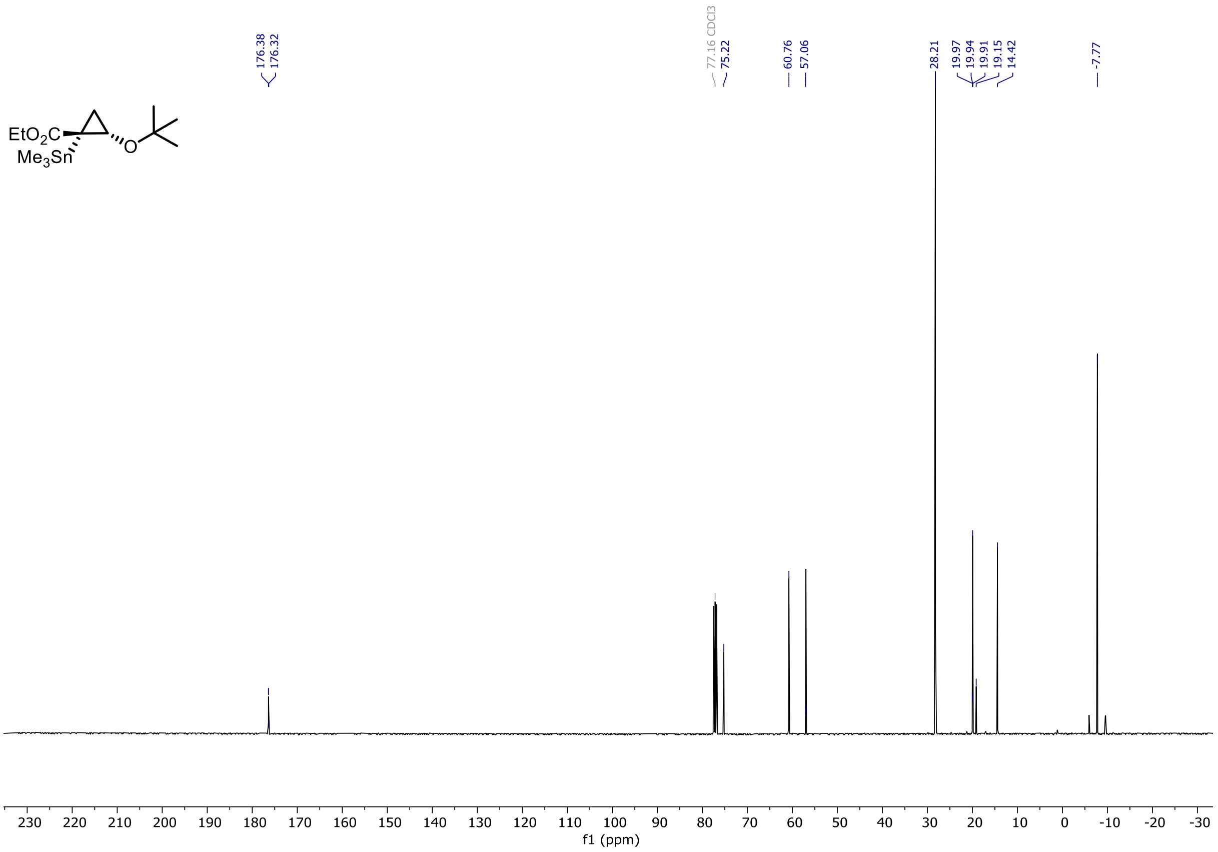

Compound 3g: ^{119}Sn NMR (149 MHz, $CDCl_3$):

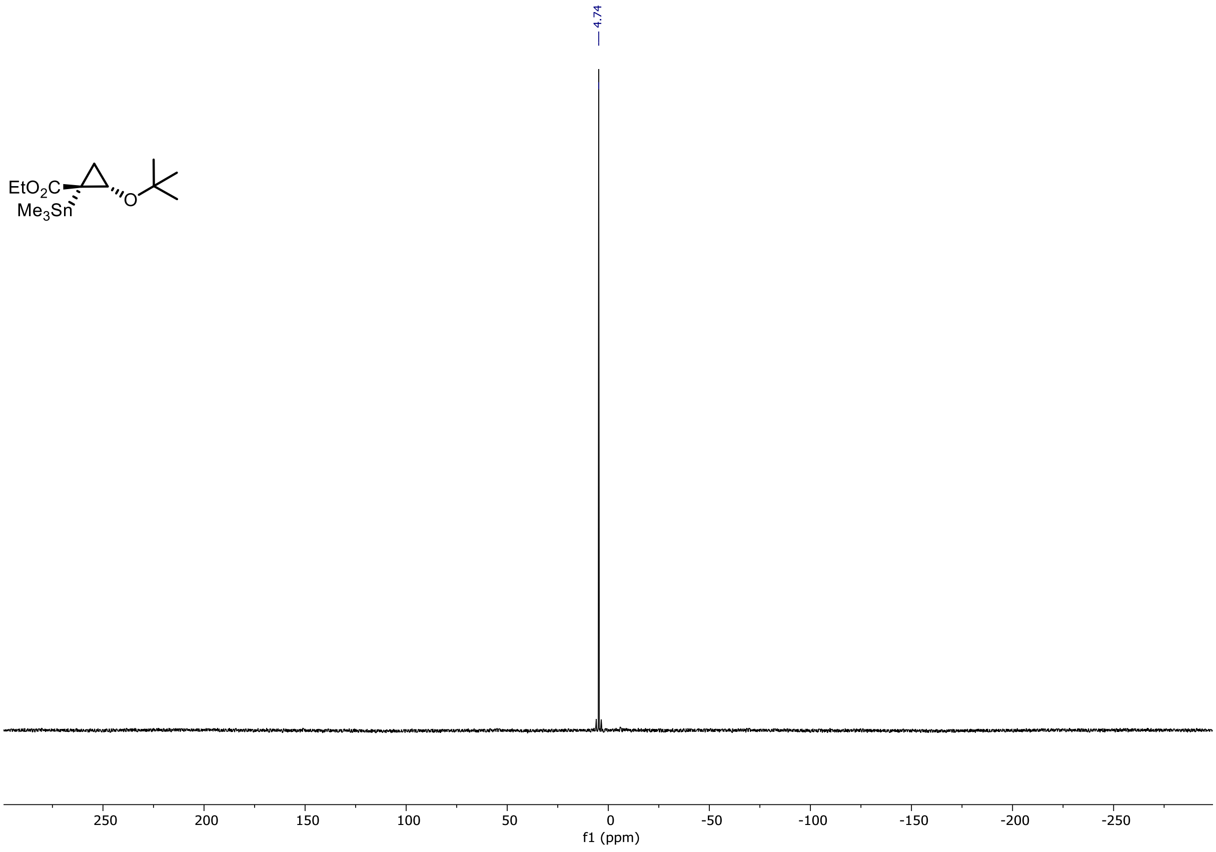

Compound 3h: ^{1}H NMR (400 MHz, $CDCl_3$) (containing ≤ 5% of the *trans*-isomer):

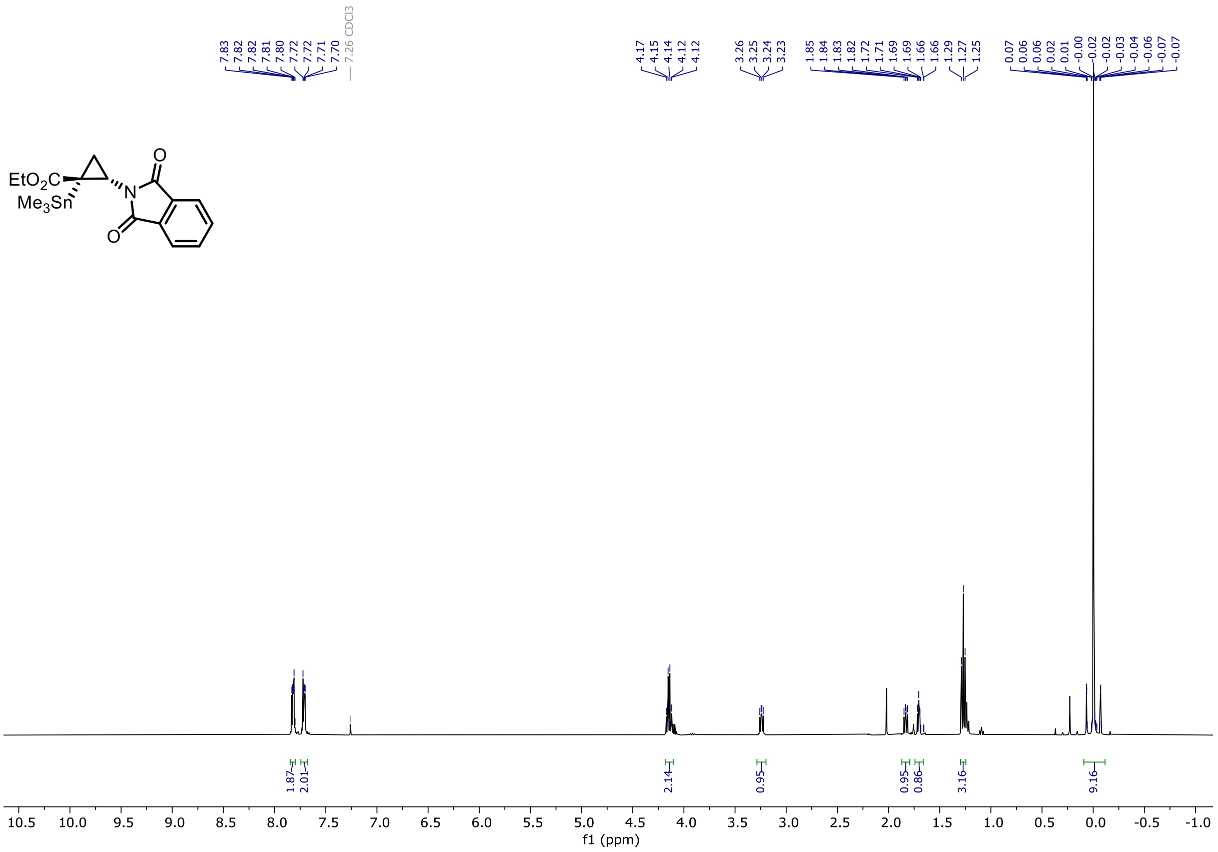

Compound 3h: ^{13}C NMR (101 MHz, $CDCl_3$) (containing ≤ 5% of the *trans*-isomer):

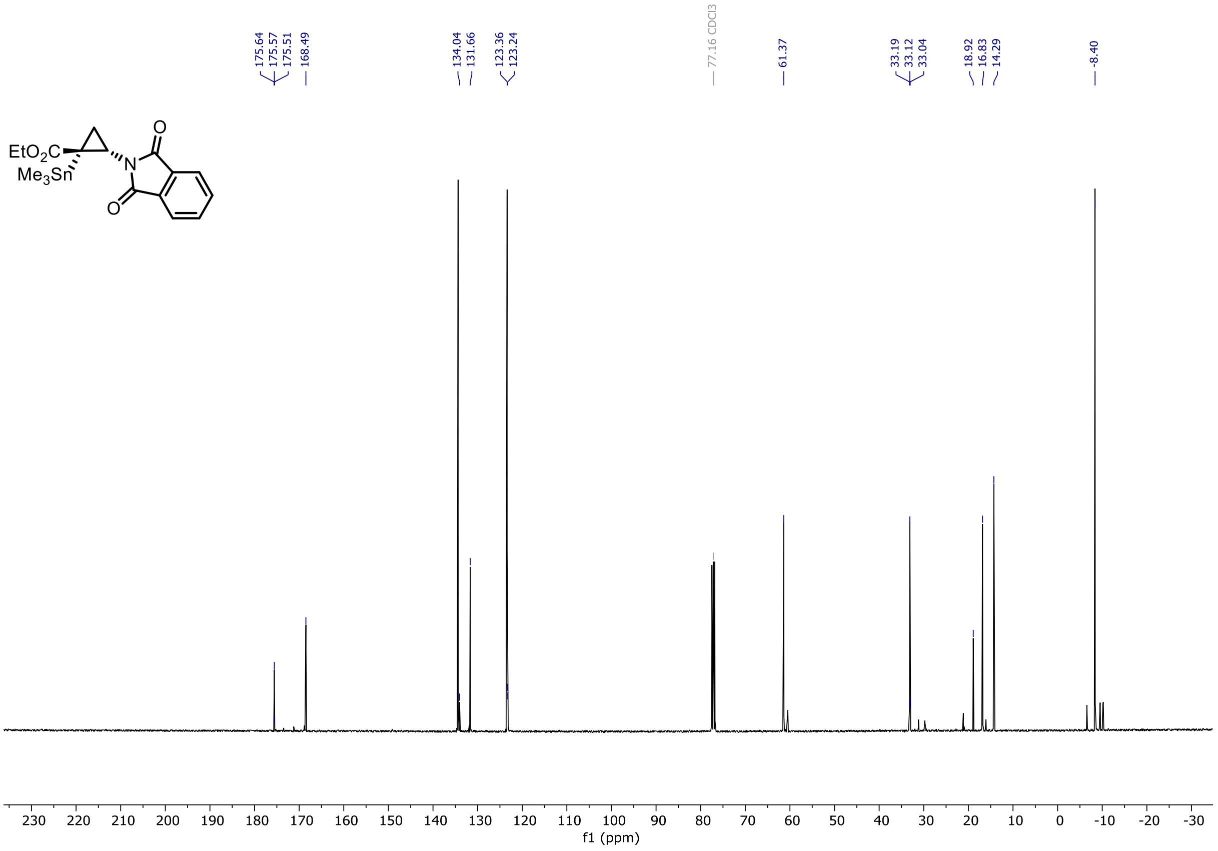

Compound 3h: ^{119}Sn NMR (149 MHz, $CDCl_3$) (containing ≤ 5% of the *trans*-isomer):

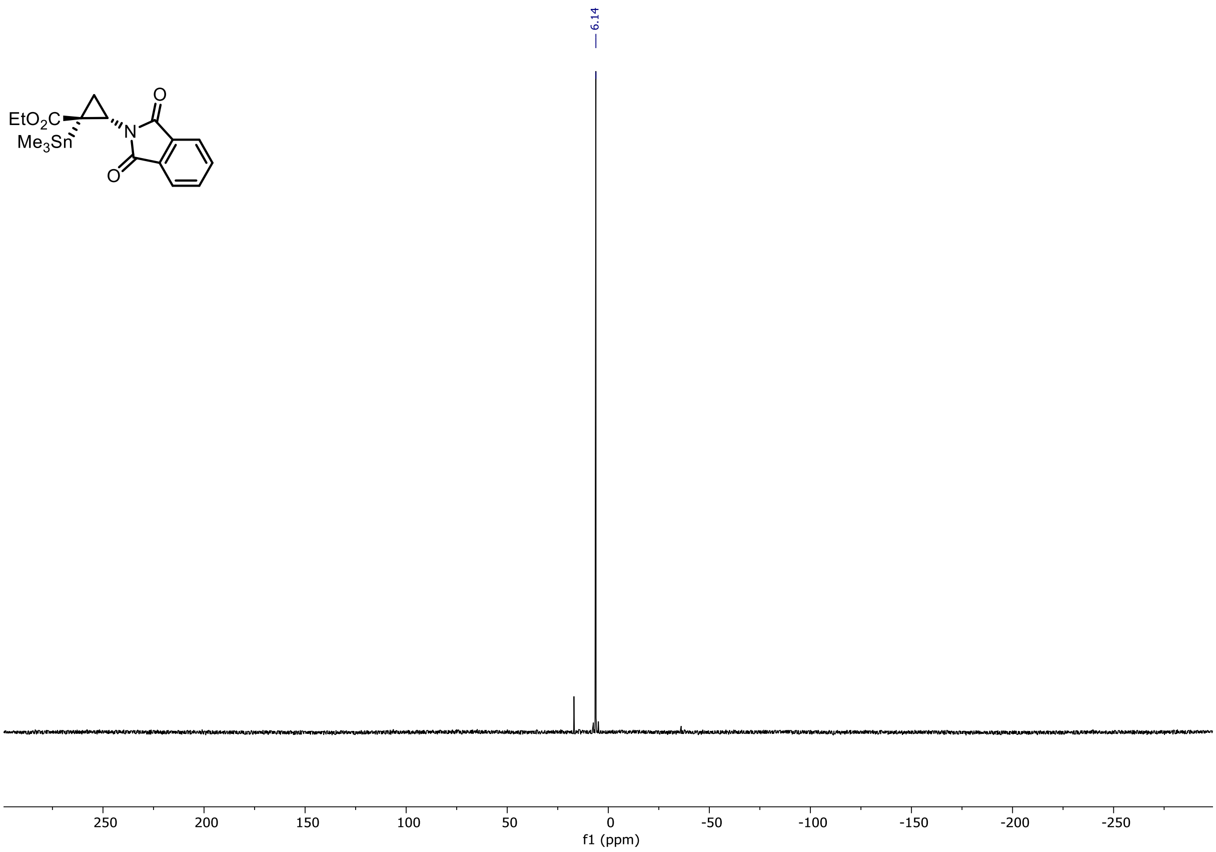

Compound 3i: ^{1}H NMR (400 MHz, $CDCl_3$) (*cis/trans* mixture):

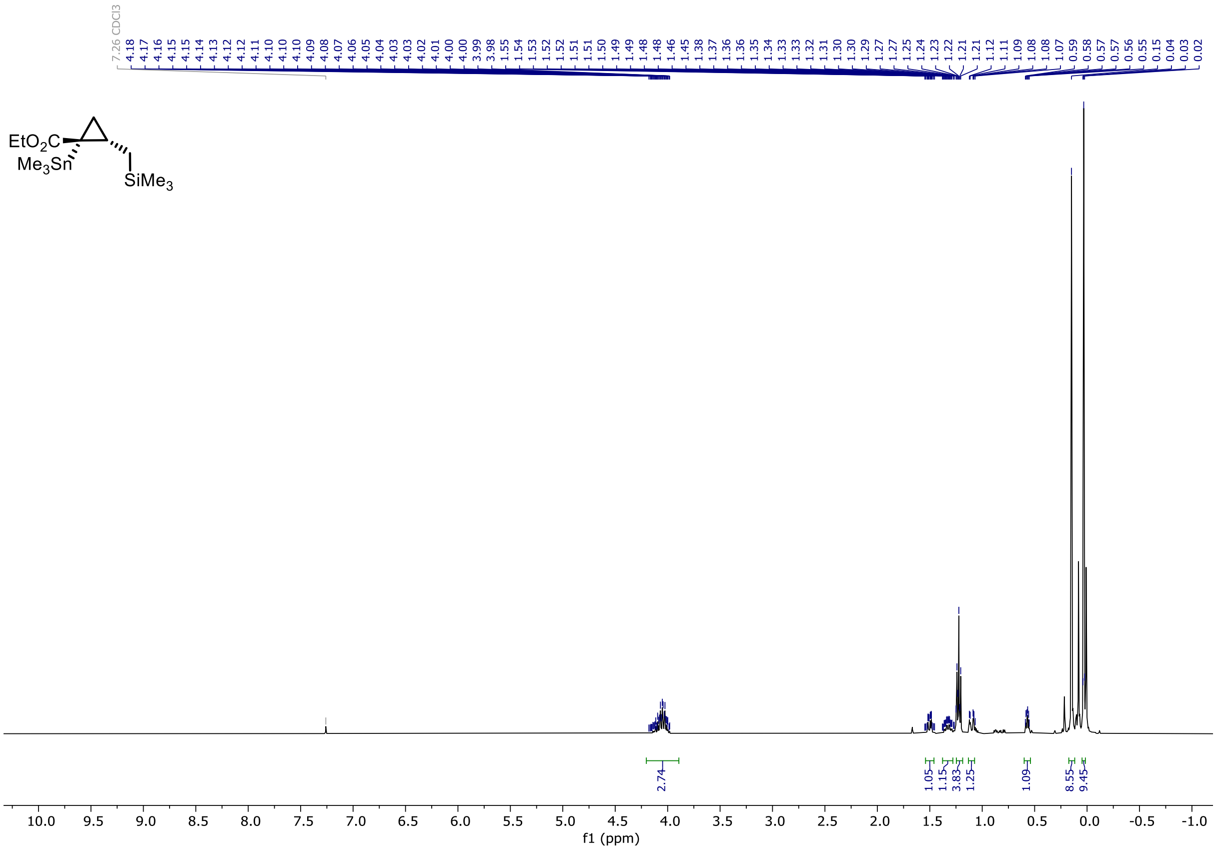

Compound 3i: ^{13}C NMR (101 MHz, $CDCl_3$) (*cis/trans* mixture):

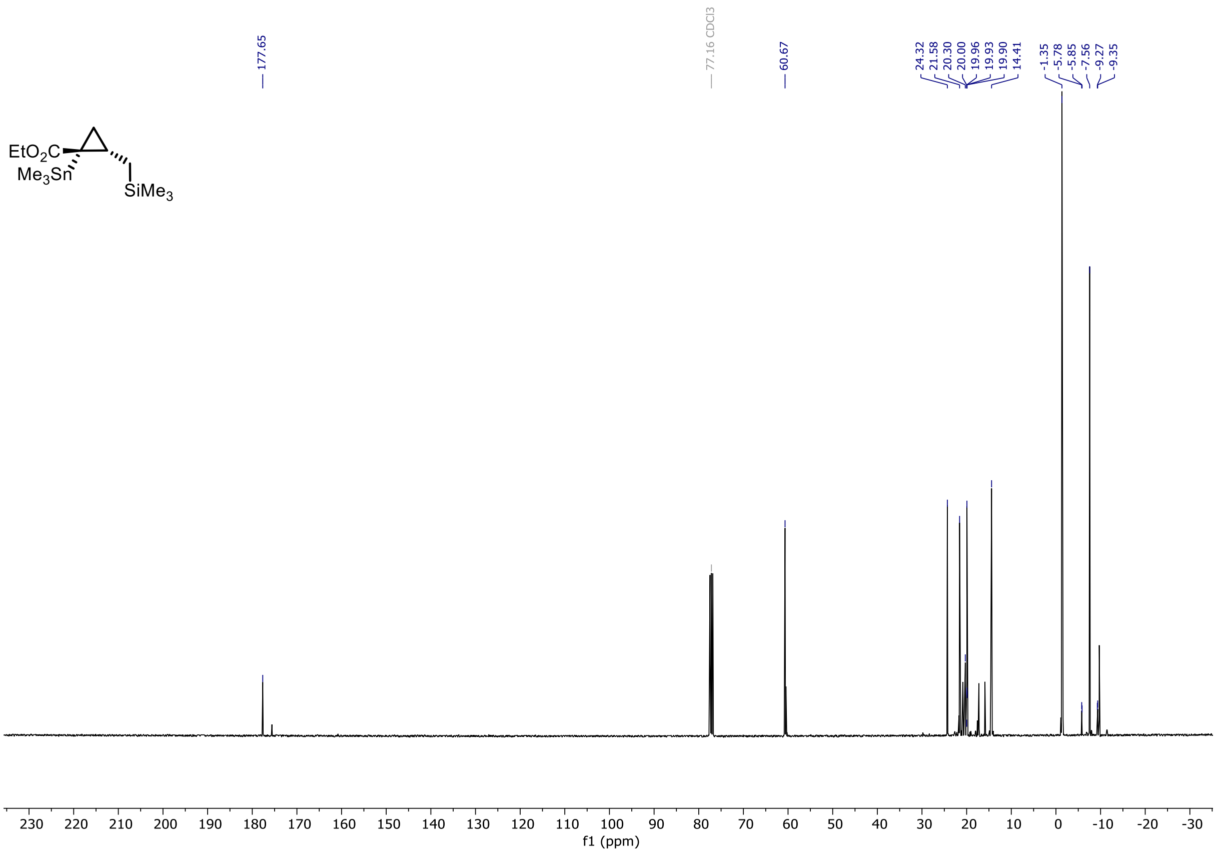

Compound 3i: ^{119}Sn NMR (149 MHz, $CDCl_3$) (*cis/trans* mixture):

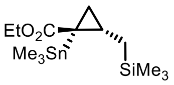

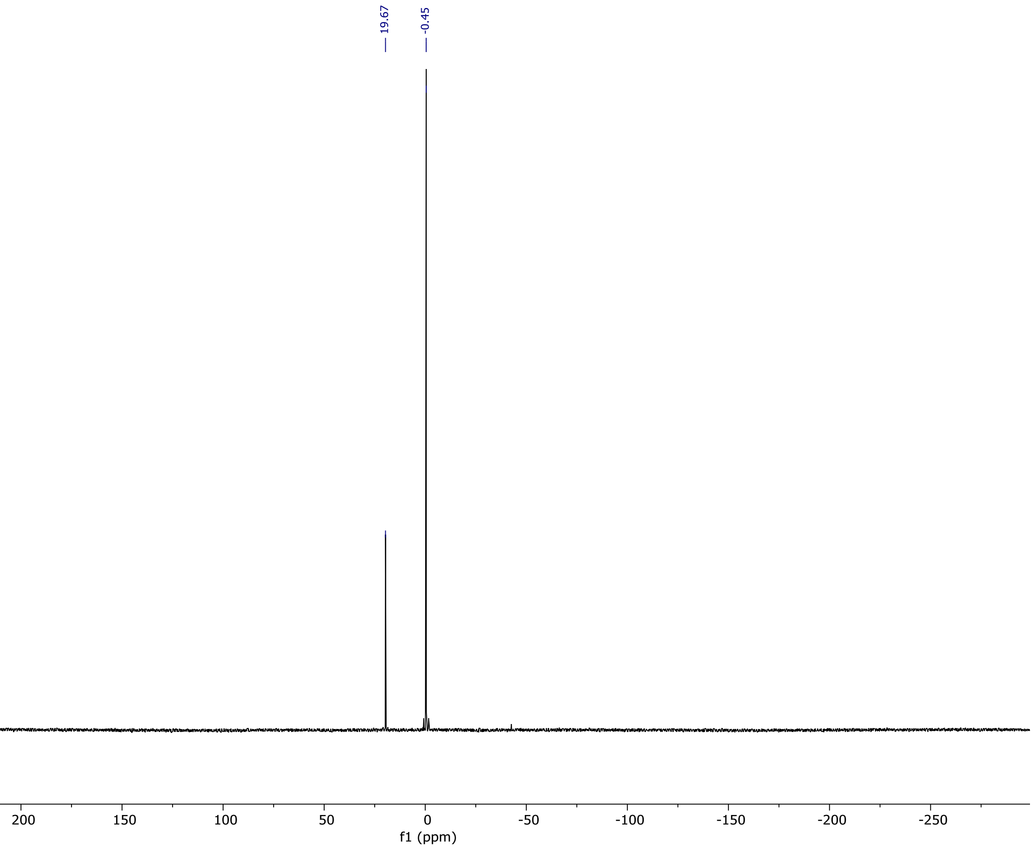

Compound 10: ^{1}H NMR (600 MHz, $CDCl_3$):

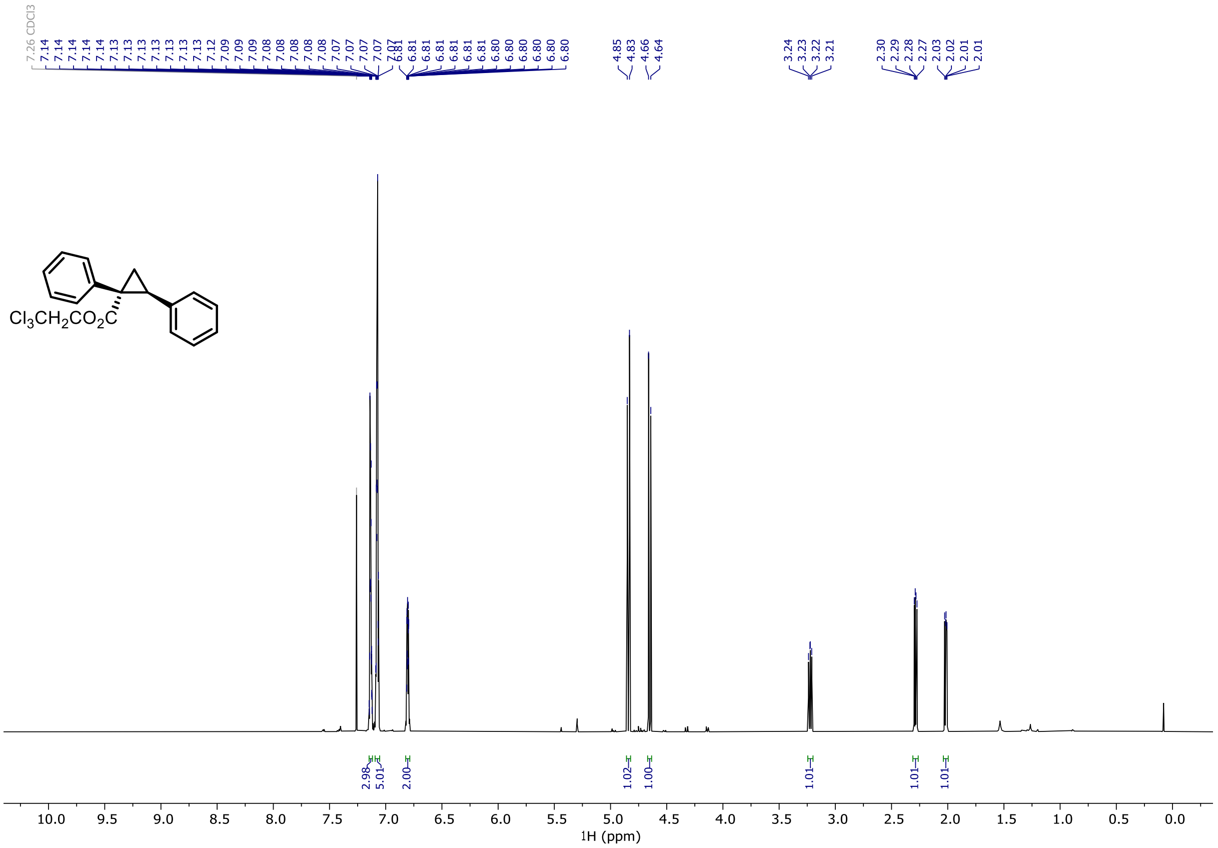

Compound 10: ^{13}C NMR (151 MHz, $CDCl_3$):

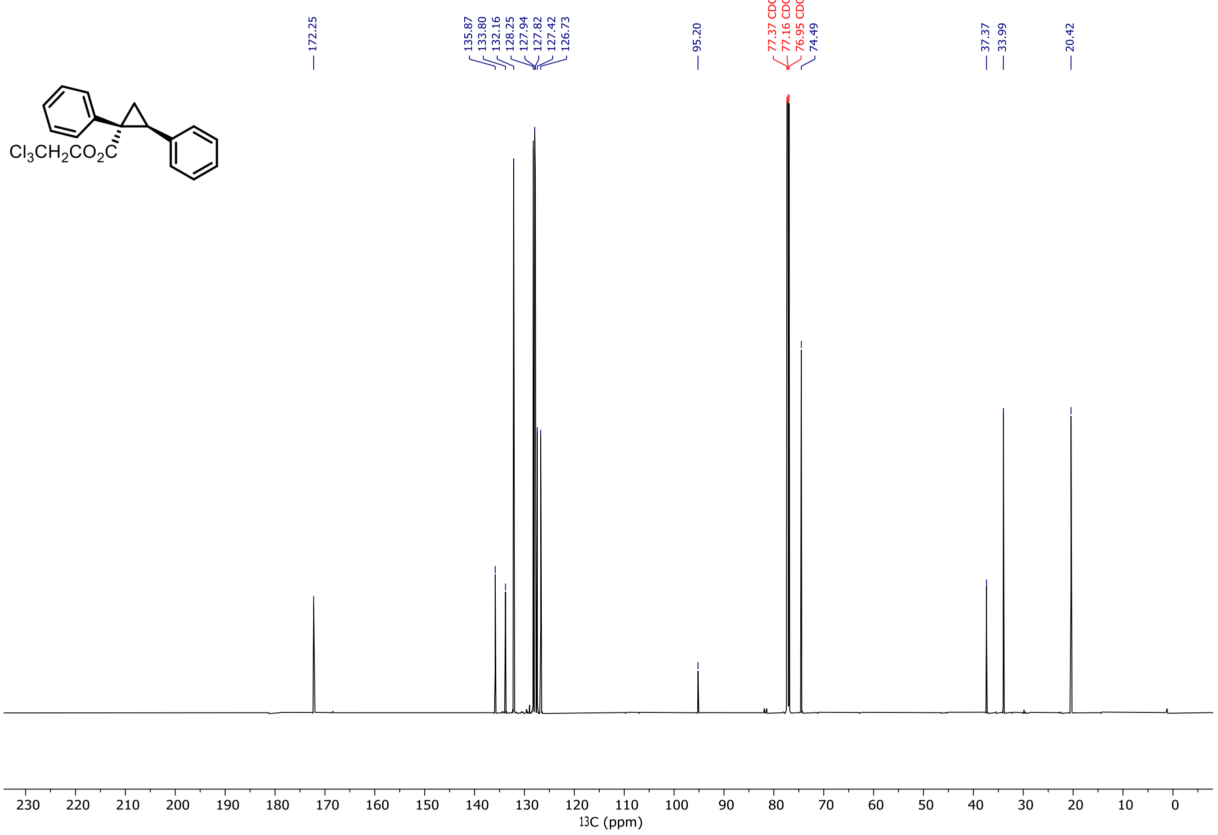

Compound 12: ^{1}H NMR (600 MHz, $CDCl_3$):

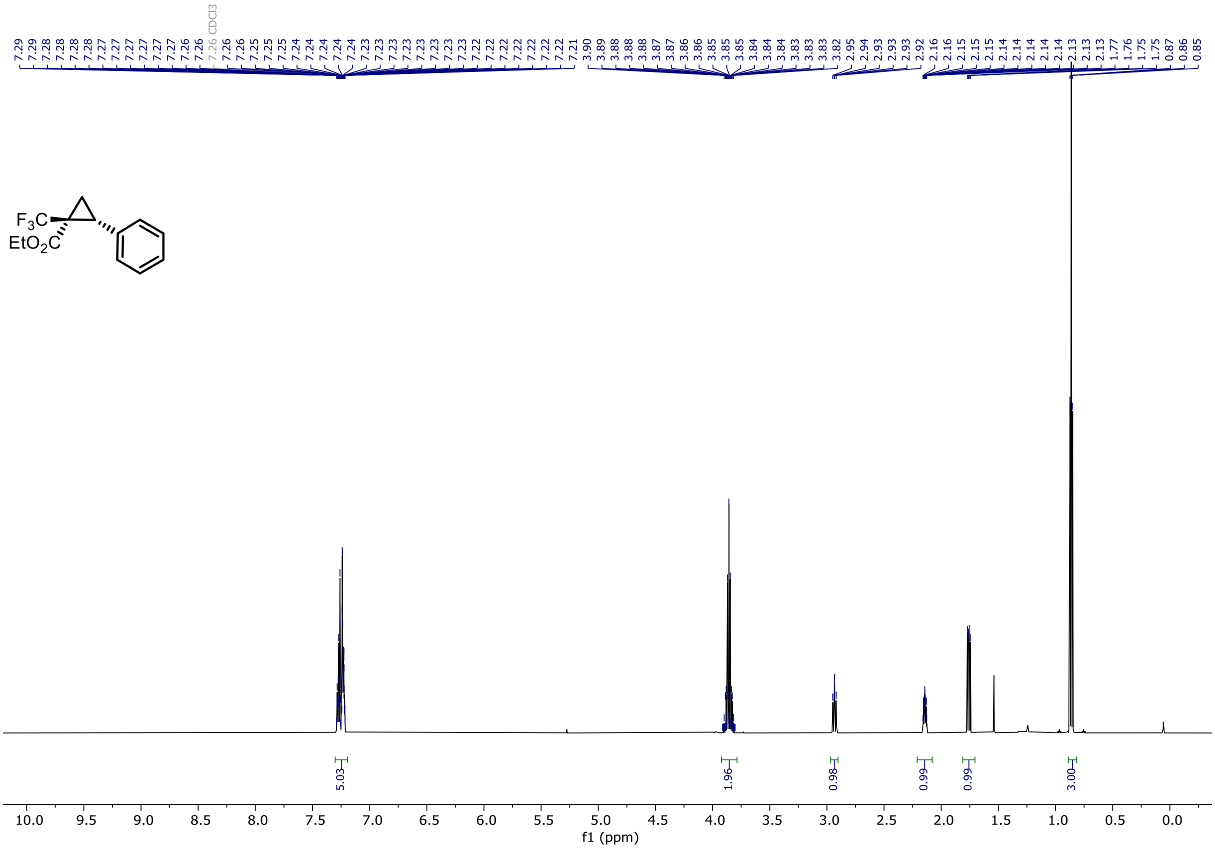

Compound 12: ^{13}C NMR (151 MHz, $CDCl_3$):

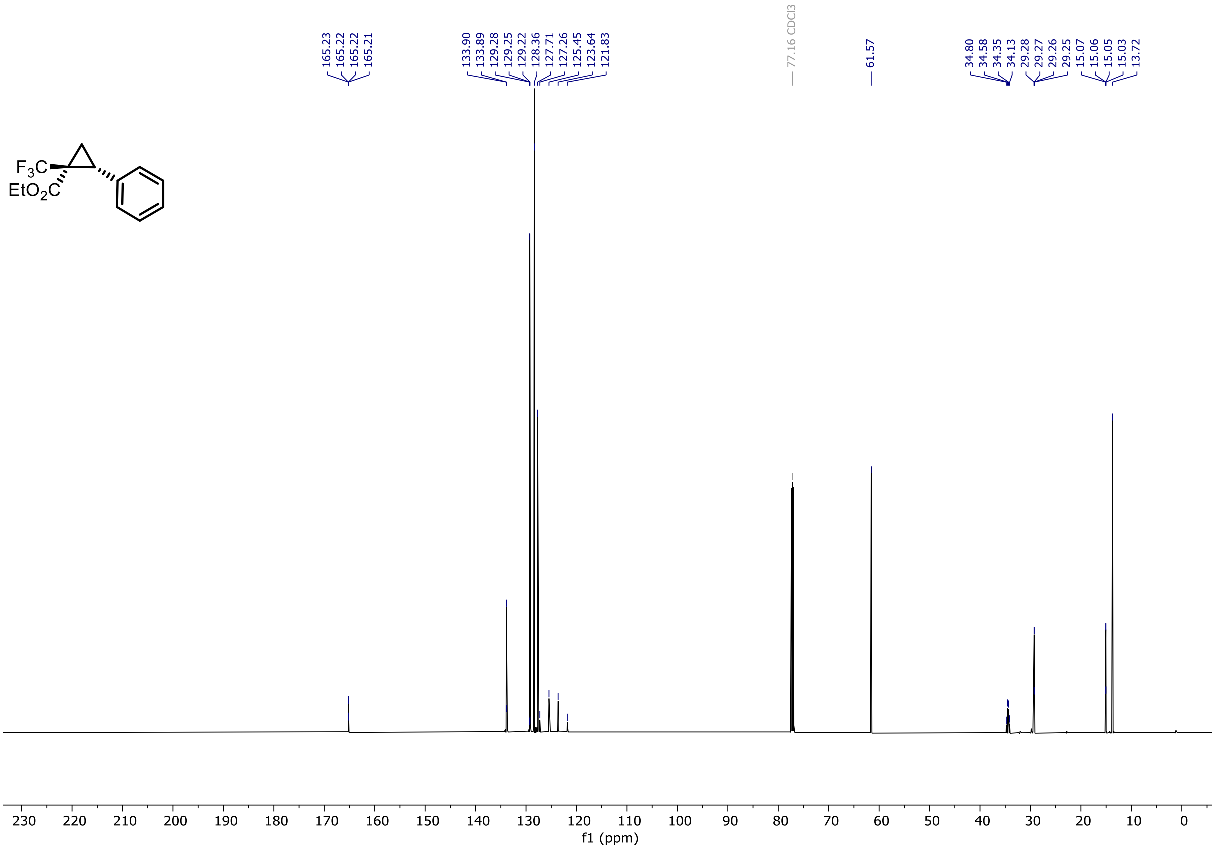

Compound 12: ^{19}F NMR (565 MHz, $CDCl_3$):

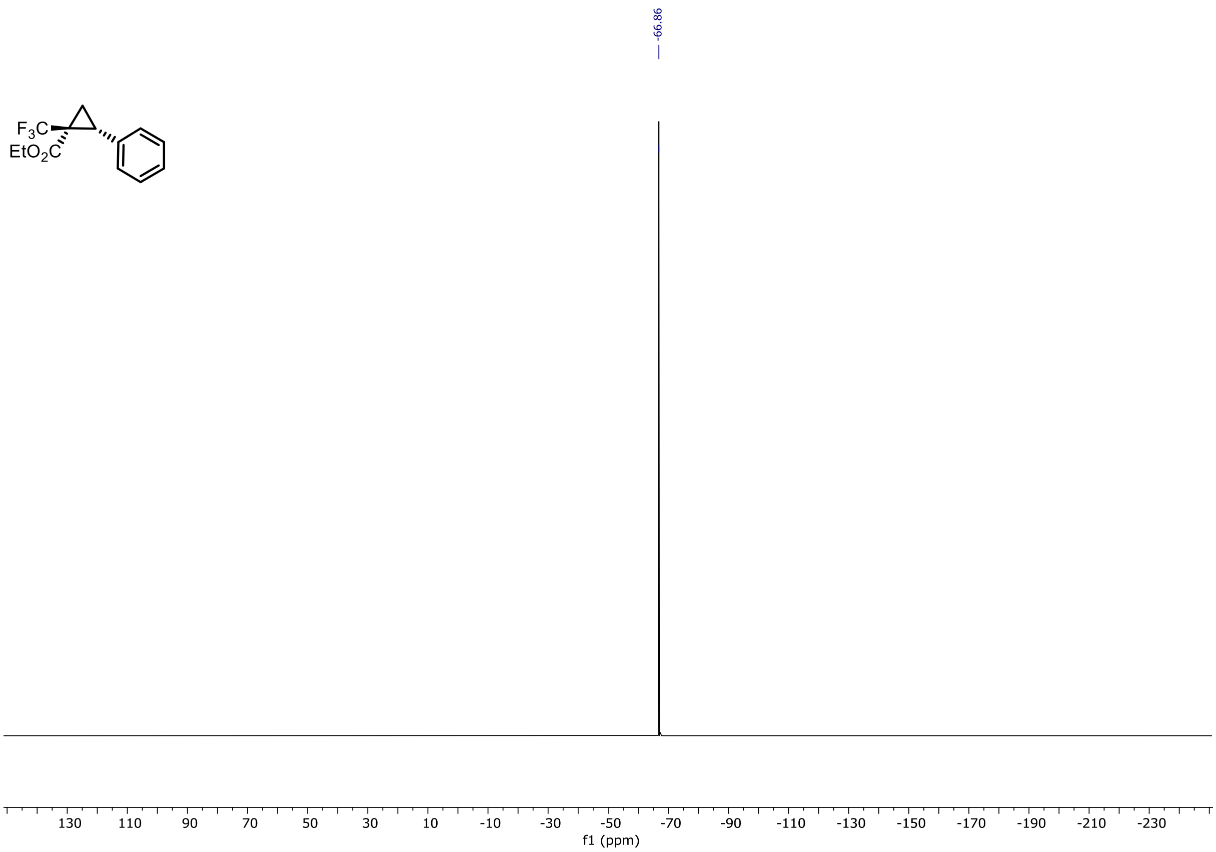

Compound 12: ^{1}H-^{1}H NOESY ($CDCl_3$):

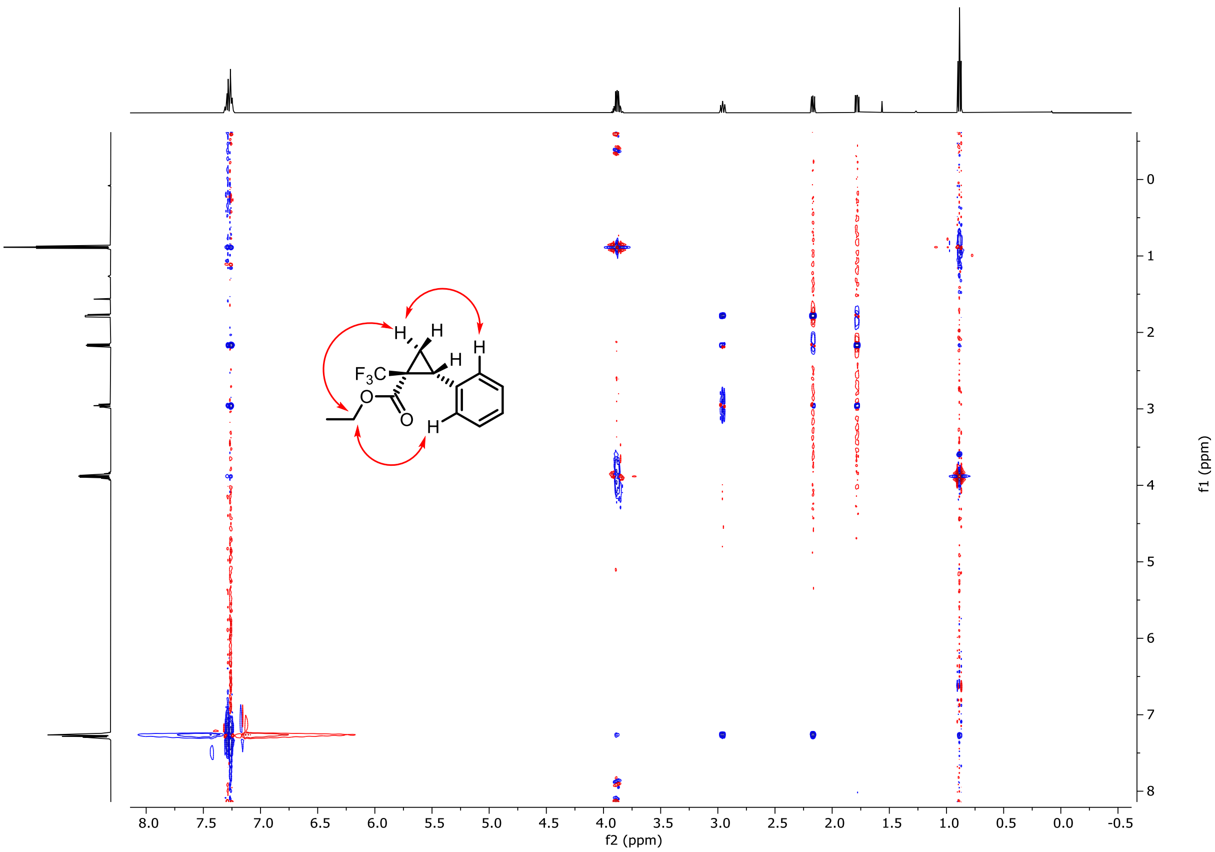

Compound 12: ^{1}H-^{19}F HOESY ($CDCl_3$):

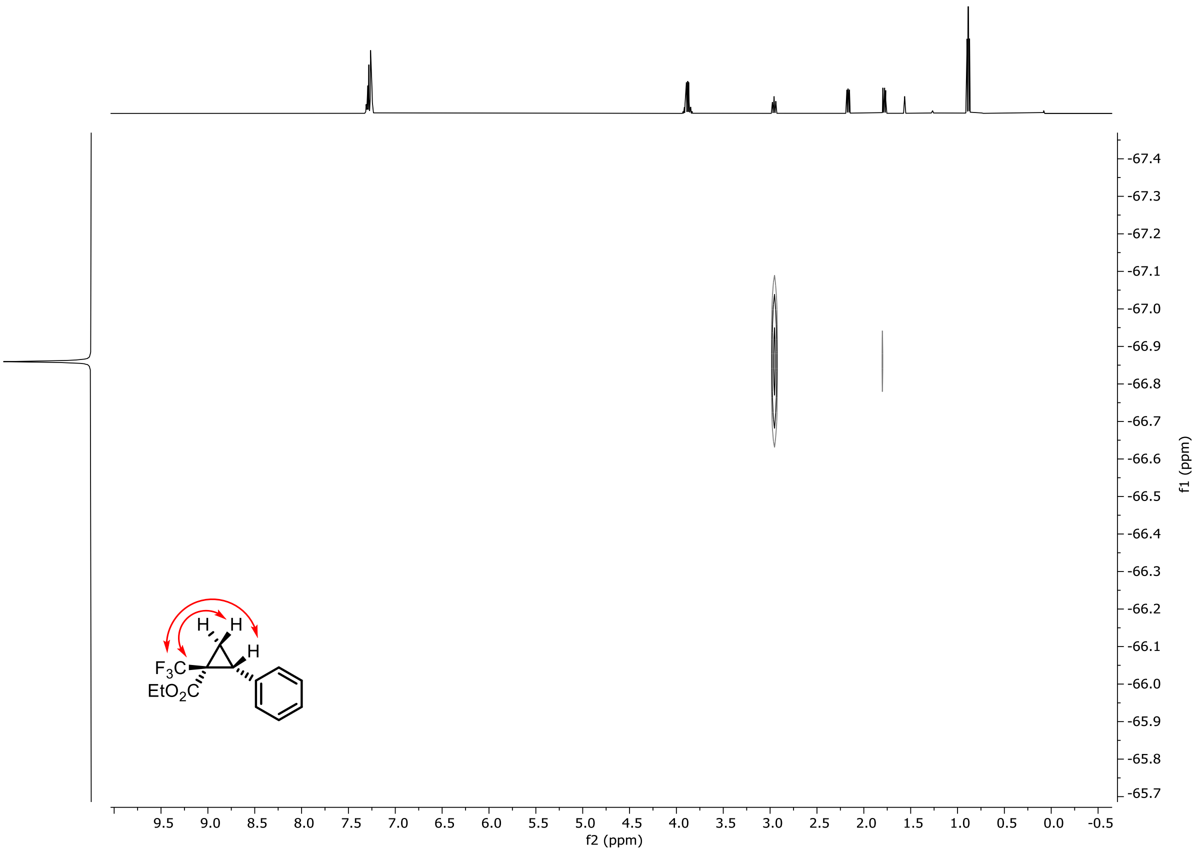

References

1. IUPAC Red Book, Nomenclature of Inorganic Chemistry, N. G. Connelly, T. Damhus, Senior ed., RSC Publishing, 2005; https://iupac.org/what-we-do/books/redbook/
2. Gade, L. H., Koordinationschemie, Wiley-VCH Weinheim, 1998
3. Taber, D. F.; Malcolm, S. C.; Bieger, K.; Lahuerta, P.; Sanaú, M.; Stiriba, S.-E.; Pérez-Prieto, J.; Monge, M. A., Synthesis, Structure, and Reactivity of the First Enantiomerically Pure Ortho-Metalated Rhodium(II) Dimer. *J. Am. Chem. Soc.* **1999,** *121* (4), 860-861.
4. Pracht, P.; Bohle, F.; Grimme, S., Automated exploration of the low-energy chemical space with fast quantum chemical methods. *Physical Chemistry Chemical Physics* **2020,** *22* (14), 7169-7192.
5. Grimme, S.; Ehrlich, S.; Goerigk, L., Effect of the Damping Function in Dispersion Corrected Density Functional Theory. *J. Comput. Chem.* **2011,** *32*, 1456.
6. Spicher, S.; Grimme, S., Robust Atomistic Modeling of Materials, Organometallic, and Biochemical Systems. *Angew. Chem. Int. Ed.* **2020,** *59* (36), 15665-15673.
7. Bannwarth, C.; Ehlert, S.; Grimme, S., GFN2-xTB – An Accurate and Broadly Parametrized Self-Consistent Tight-Binding Quantum Chemical Method with Multipole Electrostatics and Density-Dependent Dispersion Corrections. *J. Chem. Theory Comput.* **2019,** *15*, 1652.
8. Neese, F., Software update: The ORCA program system—Version 5.0. *WIREs Computational Molecular Science* **2022,** *12* (5), e1606.
9. Becke, A. D., A new mixing of Hartree–Fock and local density-functional theories. *The Journal of Chemical Physics* **1993,** *98* (2), 1372-1377.
10. Lee, C.; Yang, W.; Parr, R. G., Development of the Colle-Salvetti Correlation-Energy Formula into a Functional of the Electron Density. *Phys. Rev. B: Condens. Matter Mater. Phys.* **1988,** *37*, 785-789.
11. Vosko, S. H.; Wilk, L.; Nusair, M., Accurate spin-dependent electron liquid correlation energies for local spin density calculations: a critical analysis. *Canadian Journal of Physics* **1980,** *58* (8), 1200-1211.
12. Stephens, P. J.; Devlin, F. J.; Chabalowski, C. F.; Frisch, M. J., Ab Initio Calculation of Vibrational Absorption and Circular Dichroism Spectra Using Density Functional Force Fields. *J. Phys. Chem.* **1994,** *98*, 11623-11627.
13. Grimme, S.; Antony, J.; Ehrlich, S.; Krieg, H., A Consistent and Accurate Ab Initio Parametrization of Density Functional Dispersion Correction (DFT-D) for the 94 Elements H-Pu. *J. Chem. Phys.* **2010,** *132*, 154104.
14. Weigend, F.; Ahlrichs, R., Balanced Basis Sets of Split Valence, Triple Zeta Valence and Quadruple Zeta Valence Quality for H to Rn: Design and Assessment of Accuracy. *Phys. Chem. Chem. Phys.* **2005,** *7*, 3297.
15. Barone, V.; Cossi, M., Quantum Calculation of Molecular Energies and Energy Gradients in Solution by a Conductor Solvent Model. *J. Phys. Chem. A* **1998,** *102*, 1995-2001.
16. Cossi, M.; Rega, N.; Scalmani, G.; Barone, V., Energies, structures, and electronic properties of molecules in solution with the C-PCM solvation model. *Journal of Computational Chemistry* **2003,** *24* (6), 669-681.
17. Eichkorn, K.; Treutler, O.; Öhm, H.; Häser, M.; Ahlrichs, R., Auxiliary basis sets to approximate Coulomb potentials. *Chem. Phys. Lett.* **1995,** *240* (4), 283-290.
18. Neese, F., An improvement of the resolution of the identity approximation for the formation of the Coulomb matrix. *Journal of Computational Chemistry* **2003,** *24* (14), 1740-1747.
19. Weigend, F., Accurate Coulomb-fitting basis sets for H to Rn. *Physical Chemistry Chemical Physics* **2006,** *8* (9), 1057-1065.
20. Chai, J.-D.; Head-Gordon, M., Long-range corrected hybrid density functionals with damped atom–atom dispersion corrections. *Physical Chemistry Chemical Physics* **2008,** *10* (44), 6615-6620.

21. Falivene, L.; Cao, Z.; Petta, A.; Serra, L.; Poater, A.; Oliva, R.; Scarano, V.; Cavallo, L., Towards the online computer-aided design of catalytic pockets. *Nat Chem* **2019,** *11* (10), 872-879.
22. Fulmer, G. R.; Miller, A. J. M.; Sherden, N. H.; Gottlieb, H. E.; Nudelman, A.; Stoltz, B. M.; Bercaw, J. E.; Goldberg, K. I., NMR Chemical Shifts of Trace Impurities: Common Laboratory Solvents, Organics, and Gases in Deuterated Solvents Relevant to the Organometallic Chemist. *Organometallics* **2010,** *29* (9), 2176-2179.
23. Peeters, M.; Baldinelli, L.; Leutzsch, M.; Caló, F.; Auer, A. A.; Bistoni, G.; Fürstner, A., In Situ Observation of Elusive Dirhodium Carbenes and Studies on the Innate Role of Carboxamidate Ligands in Dirhodium Paddlewheel Complexes: A Combined Experimental and Computational Approach. *J. Am. Chem. Soc.* **2024,** *146* (38), 26466-26477.
24. Larsen, M.; Jørgensen, M., Selective Halogen−Lithium Exchange Reaction of Bromine-Substituted 25,26,27,28-Tetrapropoxycalix[4]arene. *The Journal of Organic Chemistry* **1996,** *61* (19), 6651-6655.
25. Caló, F. P.; Fürstner, A., A Heteroleptic Dirhodium Catalyst for Asymmetric Cyclopropanation with α-Stannyl α-Diazoacetate. "Stereoretentive" Stille Coupling with Formation of Chiral Quarternary Carbon Centers. *Angew. Chem. Int. Ed.* **2020,** *59* (33), 13900-13907.
26. Tortoreto, C.; Rackl, D.; Davies, H. M. L., Metal-Free C–H Functionalization of Alkanes by Aryldiazoacetates. *Org. Lett.* **2017,** *19* (4), 770-773.
27. Shi, G.; Xu, Y., Ethyl 3-trifluoro-2-diazo-propionate as a potentially useful CF3-containing building block: preparation and [Rh(OAc)2]2-catalysed reaction with nitriles. *J. Chem. Soc., Chem. Commun.* **1989,** (10), 607-608.
28. Espino, C. G.; Fiori, K. W.; Kim, M.; Du Bois, J., Expanding the Scope of C−H Amination through Catalyst Design. *J. Am. Chem. Soc.* **2004,** *126* (47), 15378-15379.
29. Caló, F. P.; Zimmer, A.; Bistoni, G.; Fürstner, A., From Serendipity to Rational Design: Heteroleptic Dirhodium Amidate Complexes for Diastereodivergent Asymmetric Cyclopropanation. *J. Am. Chem. Soc.* **2022,** *144* (16), 7465-7478.
30. Singha, S.; Buchsteiner, M.; Bistoni, G.; Goddard, R.; Fürstner, A., A New Ligand Design Based on London Dispersion Empowers Chiral Bismuth–Rhodium Paddlewheel Catalysts. *J. Am. Chem. Soc.* **2021,** *143* (15), 5666-5673.
